# The Effective Synthesis of New Benzoquinoline Derivatives as Small Molecules with Anticancer Activity

**DOI:** 10.3390/ph17010052

**Published:** 2023-12-28

**Authors:** Gheorghita Zbancioc, Ionel I. Mangalagiu, Costel Moldoveanu

**Affiliations:** 1 Chemistry Department, Alexandru Ioan Cuza University of Iasi, 11 Carol 1st Bvd, 700506 Iasi, Romania; 2 Institute of Interdisciplinary Research-CERNESIM Centre, Alexandru Ioan Cuza University of Iasi, 11 Carol I, 700506 Iasi, Romania

**Keywords:** anticancer, benzo[*c*]quinoline derivatives, quaternary salts, cycloadducts, microwave, ultrasound

## Abstract

In this study, some novel benzo[*c*]quinoline derivatives were synthesized, their structural characteristics were described, and their in vitro anticancer efficacy was investigated. The synthesis involves an initial quaternization of the nitrogen atom from benzo[*c*]quinoline and then a [3+2] dipolar cycloaddition reaction of the in situ formed ylide. The effectiveness of synthesis using traditional thermal heating (TH) compared to microwave (MW) and ultrasound (US) irradiation was investigated in detail. The setup of a reaction under MW or US irradiation offers a number of additional benefits: higher yields, a reduction in the amount of solvent used compared to TH, a reduction in the reaction time from hours to minutes, and a reduction in the amount of energy consumed. The structure of all the obtained compounds was proved by several spectral techniques (FTIR, HRMS, and NMR). All benzo[*c*]quinoline derivatives (quaternary salts and cycloadducts) along with ten other benzo[*f*]quinoline derivatives (quaternary salts and cycloadducts), previously obtained, were tested in an in vitro single-dose anticancer experiment. The results demonstrated that the cycloadducts **5a–c** and **6a–c** exhibit stronger anticancer activity than quaternary salts **3a–c**. The most active compound is compound **5a**, with anticancer activity on most of the cell lines studied, while the second most active compound is **6c**, showing significant lethality for the SR leukemia cell line (17%). Structure-activity relationship (SAR) correlations are also included in the study.

## 1. Introduction

The polynuclear azaheterocycle benzo[*c*]quinoline has potential applications in optoelectronics, agriculture, and other fields [1,2]. Recent studies have shown that incorporating the benzoquinoline skeleton into the design of some compounds can be advantageous, leading to new derivatives with biological properties such as antimicrobial [3,4] and anticancer activity [5,6,7]. This is true even though the native heterocycle is known to be an environmental pollutant [8,9] and has been detected in coal tar [10,11], petroleum distillate [12], and urban air particles with genetic effects [12]. Furthermore, a number of derivatives have been created and synthesized based on the fluorescence characteristics of benzoquinoline [13], and it has been demonstrated that some of these compounds have the potential to be used in organic light-emitting diodes (OLEDs) [14,15,16].

Cancer is a deadly illness, and some kinds are marked by quick development and widespread bodily obliteration [17]. The World Health Organization (WHO) estimated more than 19 million new cancer cases and nearly 10 million cancer deaths in 2020, which makes cancer the second largest cause of death worldwide [18]. Treatment for diverse cancer types can be challenging and involves surgery, radiation, chemotherapy, hormone therapy, immunotherapy, stem cell transplant, as well as a combination of some of these methods [19,20,21].

One cancer treatment that can lengthen life expectancy is chemotherapy. Life expectancy was 46.8 years when the first substance with anticancer action hit the market in 1949; it rose to 71.4 years in 2015 when about 160 anticancer medications were approved for clinical use [22,23]. The market’s current medications, however, have a poor efficacy rate and numerous disadvantages [24,25,26]. The need for new anticancer medications in therapy is therefore critical.

Recent years have seen a rise in the use of microwave and ultrasonic-assisted reactions in synthetic organic chemistry [27,28,29,30], providing a quick and practical alternative in a wide range of syntheses [31,32,33,34,35,36]. Both microwave and ultrasound irradiation provide several significant advantages over traditional thermal heating (TH), including greater yields, high purity of the compounds, increased selectivity, decreased reaction times, cheaper costs, and improved ease of handling and processing. Reactions under MW and US irradiation could be viewed as environmentally beneficial [37,38] in light of all these benefits.

Given the aforementioned factors, our research focuses on creating new anticancer substances using various azaheterocycle scaffolds that may be used in chemotherapy to treat cancer [39,40]. In this context, we tried to evaluate the biological activity of the previously obtained benzo[*f*]quinoline derivatives [13] and replace the benzo[*f*]quinoline core with benzo[*c*]quinoline in order to obtain new hybrid conjugated molecules with potential anticancer activity. Furthermore, we decided to research the synthesis of benzo[*c*]quinoline derivatives using traditional TH, MW, and US irradiation. We were equally interested in creating brand-new, environmentally friendly processes to prepare these compounds using MW and US technologies.

## 2. Results and Discussion

### 2.1. Design and Chemistry

**Scheme 1:**
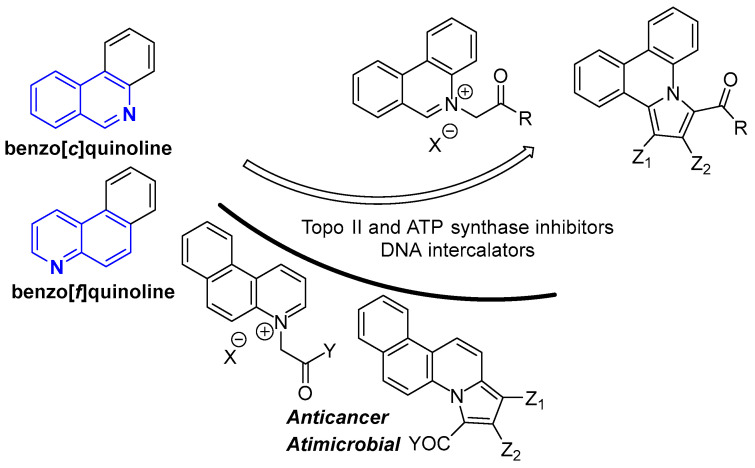
Design of the class of benzo[*c*]quinolines derivatives with anticancer and antimicrobial activity.

Benzo[*c*]quinoline is an azaheterocycle structurally similar to benzo[*f*]quinoline, Scheme 1. In previous research work [3,4], we found that benzo[*f*]quinoline derivatives with polycyclic skeleton possess various biological activities, including anticancer and antimicrobial activity. Moreover, as to the mechanism of action, we found that these compounds are possible Topo II and ATP synthase inhibitors [3,4]. Some other research groups proved that pyrrolophenanthridines derivatives (analogous to pyrrolophenanthridines **5** and **6**) are potential Topo II inhibitors [41]. Benzo[*c*]quinoline derivatives can intercalate to the DNA double helix, forming stable sandwich-like structures. Their ability to act as DNA double helix intercalators is the basis of their anticancer activity [42,43]. On the other hand, natural compounds with a benzo[*c*]quinoline skeleton, such as alkaloids sanguinarine and chelerythrine and their synthetic analogs, are well known for their anticancer activity [44,45,46]. Having in view the above considerations, it appears logical for us to presume that polycyclic benzo[*c*]quinoline derivatives will have anticancer activity with two possible mechanisms of action as polycyclic benzo[*f*]quinoline derivatives.

**Scheme 2:**
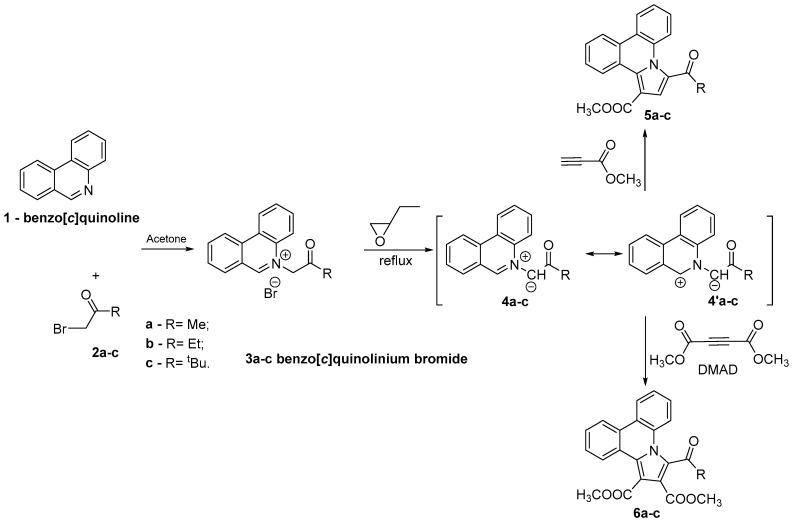
Reaction pathway to obtain benzo[*c*]quinolinium derivatives **3a–c**, **5a–c**, and **6a–c**.

The general approach taken for the synthesis of the novel polycyclic benzo[*c*]quinoline derivatives is described in Scheme 2 and follows the general strategy previously used in the synthesis of benzo[*f*]quinoline derivatives [13]. In the first step, the benzo[*c*]quinoline **1** is *N*-alkylated with bromoketones **2a–c** to produce benzo[*c*]quinolinium bromides **3a–c**. In the second step, the benzo[*c*]quinolinium ylides **4a–c** (synthesized in situ from the corresponding salts **3a–c** with 1,2-butylene oxide acting as a catalyst) are then subjected to a Huisgen [3+2] dipolar cycloaddition to the alkyne dipolarophiles (methyl propiolate or dimethyl acetylenedicarboxylate—DMAD), resulting in cycloadducts **5a–c** and **6a–c** as the final products.

The benzo[*c*]quinolinium salts and azatetracyclic derivatives were produced in yields ranging from 46 to 72%, as shown in Table 1. The main drawbacks of the synthesis performed under standard conditions are the lengthy reaction time (36 h in the case of benzo[*c*]quinolinium salts and 48 h in the case of cycloadducts) and significant energy usage.

As a more energy-efficient alternative, we have synthesized azatetracyclic derivatives using MW and US radiation. A monomode reactor (Monowave 300; Anton Paar, Graz, Austria) was utilized for MW irradiation reactions. This reactor permits stirring of the reaction mixture (0 to 1200 rpm) and has temperature control up to 300 °C. The reactions occur at a maximum pressure of 30 bars in a closed vessel. The most suitable reaction parameters in our case were determined to be 90 °C in the case of benzo[*c*]quinolinium salts and 130 °C in the case of azatetracyclic derivatives, with the reactions finishing after 10–15 min of irradiation. We employed a Bandelin reactor (Sonopuls GM 3200, Berlin, Germany) for the US irradiation processes, with a nominal output of 200 W. We were able to control the pulse sequence, amplitude (mean percent of the nominal power), and irradiation period thanks to the equipment we employed. All of these factors were anticipated to affect the reaction. After 20 to 30 min of irradiation, the Huisgen [3+2] dipolar cycloaddition was accomplished utilizing 80% of the instrument’s nominal power.

When compared to the optimum reaction conditions employed under TH conditions, the best results obtained with the optimized reaction conditions under MW and US irradiation are reported in Table 1.

As shown in Table 1, the use of MW and US irradiation induced a remarkable acceleration for the Huisgen [3+2] dipolar cycloaddition reaction, the reaction times decreasing from 48 h to 10–15 min (under MW irradiation) or 20–30 min (under US irradiation), while in the case of the *N*-alkylation reaction the decrease was from 36 h to 10 min (under MW irradiation) or 20 min (under US irradiation). The acceleration of these reactions under unconventional heating may be attributed to the fact that both MW and US irradiation allow for far higher temperatures to be reached than would be feasible with thermal heating (temperatures higher than the boiling point of the solvent). It is also important to note that the yields were higher (by 15–30%), and the solvent amounts utilized in the former were at least three times lower under MW and US irradiation than they were under conventional TH (see Experimental. As a result, it is possible to consider these reactions to be environmentally friendly when exposed to MW and US radiation.

We assume that the MW heating strategy was more effective in *N*-alkylation and [3+2] dipolar cycloaddition reactions for two reasons: the mode of action under MW irradiation and the structure of the intermediate. This assumption is based on our prior findings [31]. According to the MW theory [28,31,33], the dielectric heating effect of MW is mostly dependent on the molecules’ dipole moments: the stronger the dipole moment, the greater the influence of the MW energy. Since benzo[*c*]quinolinium salts and corresponding ylides have outstanding dipoles, their MW heating efficiency is significantly higher than that of TH.

In the case of US irradiation, we assume that the efficiency in these reactions was caused by the cavitation phenomena, with energy being conveyed to the substrates more effectively than in reactions carried out under standard TH conditions. Additionally, when bubbles collapsed, mechanical tension was created that could be transferred to a target single bond, a phenomenon unique to ultrasonic action [29].

**Scheme 3:**
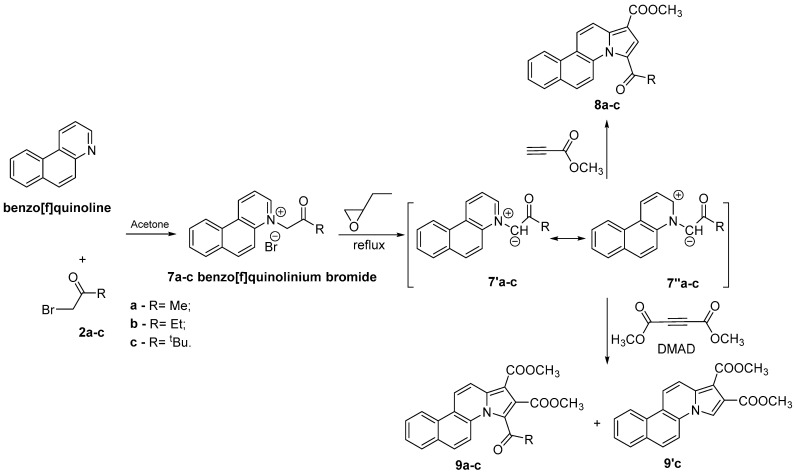
Reaction pathway to obtain benzo[*f*]quinolinium derivatives **7a–c**, **8a–c**, **9a–c,** and **9′c**.

The benzo[*f*]quinolinium salts **7a–c** and their corresponding cycloadducts **8a–c**, **9a–c**, and **9′c** (see Scheme 3) were obtained and reported in a previous study [13], and here we investigated their anticancer activity.

The compound’s identity was proved through elemental and spectral analysis (FT-IR, HRMS, ^1^H-NMR, ^13^C-NMR, 2D-COSY, HMQC, and HMBC), which can be found in the supporting information.

### 2.2. Anticancer Activity

The National Cancer Institute (NCI), USA, used its screening program for anticancer drugs to assess the produced compounds’ anticancer efficacy. As it offers a thorough evaluation of the compounds’ activity against several types of cancer cells, this program, also known as the NCI 60 cell line screen, is a useful tool for drug discovery and development. The in vitro anticancer assay uses the NCI 60 cell line screening, which consists of 60 distinct human tumor cell lines that represent a variety of malignancies, including leukemia, melanoma, and tumors of the lung, colon, brain, ovary, breast, prostate, and kidney. Following the NCI protocol, which is presented in the supporting information, the screening was completed [47,48,49,50,51,52,53].

All chosen compounds were tested against 60 cell lines in the screening process’s initial step using a single dose of 10 μM [47]. Using the COMPARE application, it is possible to examine the mean graph that represents the results of this single-dose screen [50]. The percentage growth inhibition (PGI) is used to express the results and denote growth both in comparison to the drug-free control and the initial cell count. With the help of this technique, mortality (values less than 0) and growth inhibition (values ranging from 0 to 100) can be detected. For instance, a number of 30 would mean that 70% of growth would be inhibited, whereas a value of −30 would mean that 30% of the organism would be killed.

Nine synthesized compounds were tested in a primary single-dose anticancer assay (at a concentration of 10^−5^ M), consisting of three benzo[*c*]quinolinium salts (**3a–c**) and six pyrrolobenzo[*c*]quinoline cycloadducts (**5a–c** and **6a–c**). The obtained results for all tested compounds can be found in Table 2.

**Figure 1:**
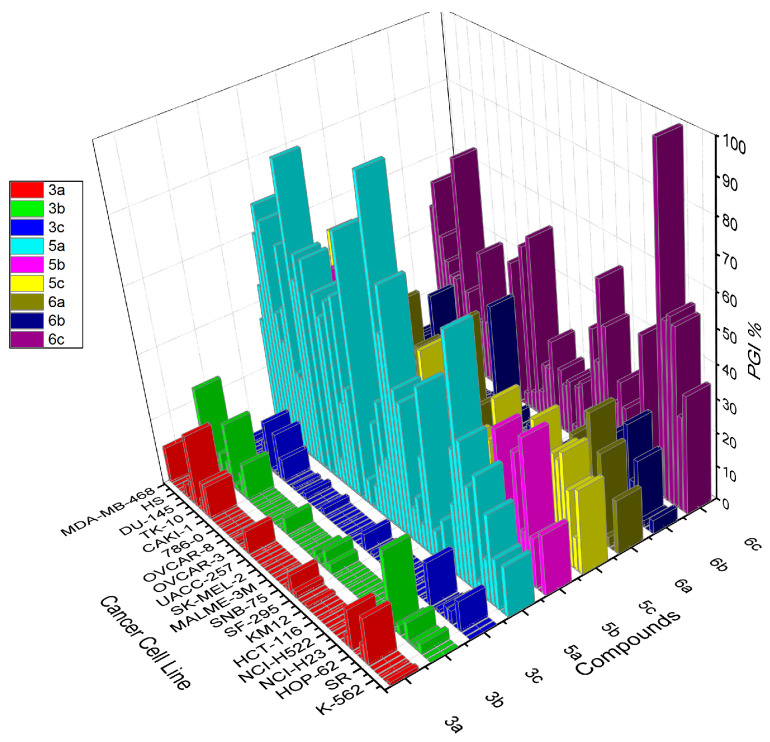
Anticancer activity of the benzo[*c*]quinolinium salts **3a–c** and pyrrolobenzo[*c*]quinoline cycloadducts **5a–c** and **6a–c** against 60 NCI human cancer cell lines, expressed as the percentage growth inhibition.

The results from Table 2 are presented in Figure 1 and in Figures S1–S9 from the supporting information.

Based on the results presented in Table 2 and Figure 1, it can be seen that in the series of benzo[*c*]quinoline derivatives, the most active compound is **5a**, with anticancer activity on most of the cell lines studied, showing significant lethality on CNS cancer on the SNB-75 cell line (12%) and PGI values higher than 60% for 10 cell lines.

The second most active compound is **6c**, which shows significant lethality for the SR leukemia cell line (17%) and PGI values above 50% for eight cell lines.

All cycloadducts show almost non-selective activity on leukemia (except for the HL-60 (TB) cell line). In the case of the HL-60 (TB) cell line, only cycloadducts **6c** (PGI 28%) and **5c** (PGI 10%) show some activity.

For prostate cancer, all cycloadducts have selective activity on the PC-3 cell line (with PGI values between 65 and 28%); for the DU-145 cell line, only compound **5a** shows some activity (PGI 33%).

In the case of leukemia, the most active compound is **6c** (with 100% PGI and 17% lethality) on the SR cell line. In Non-Small Cell Lung Cancer, the most active compound is **5a** (with 72% PGI) on the HOP-92 cell line. In colon cancer, the most active compounds are **6c** (with 54% PGI) on the HCT-15 cell line and compound **5a** (with 50% PGI) on the HCT-116 cell line. In the case of CNS cancer, the most active compound is **5a** (with 100% PGI and 12% lethality) on the SNB-75 cell line. In melanoma, the most active compound is **5a** (with 79% PGI) on the SK-MEL-5 cell line. In ovarian cancer, the most active compound is **5a** (with 59% PGI) on the IGROV1 cell line. In renal cancer, the most active compound is **5a** (with 89% PGI) on the RXF 393 cell line. In prostate cancer, the most active compound is **6c** (with 65% PGI) on the PC-3 cell line. In breast cancer, the most active compound is **5a** (with 71% PGI) on the HS 578T cell line, (with 67% PGI) on the MDA-MB-231/ATCC cell line and (with 62% PGI) on the BT-549 cell line.

The results in Table 2 show that cycloadducts **5a–c** and **6a–c** exhibit stronger anticancer activity than quaternary salts **3a–c**. Considering the structure-activity relationship (SAR), a number of preliminary conclusions can be drawn on the activity of the tested compounds.

Some of the compounds show non-selective activity on several types of cancer cell lines, while others have a more selective activity only on some cancer cell lines.

In the case of cycloadducts, those derived from methyl propiolate **5a–c** are less sterically hindered (they present a single ester group on 4th position and an acyl group on 2nd position of the pyrrolic ring) while cycloadducts **6a–c**, derived from DMAD are more sterically hindered (they present two ester group on 3rd and 4th position and an acyl group on 2nd position of the pyrrolic ring).

In the case of cycloadducts **5a–c**, the most active is compound **5a**, the least sterically hindered (with the smallest acyl group—acetyl), while in the case of cycloadducts **6a–c,** the most active is compound **6c**, the most sterically hindered (with the largest acyl group—pivaloyl).

In the series of benzo[*c*]quinoline derivatives, the higher activity of cycloadducts (compounds with tetracyclic structure) than quaternary salts (compounds with tricyclic structure) suggests that structures with 4-fused cycles are preferred to those with 3-fused cycles for showing anticancer activity.

**Figure 2:**
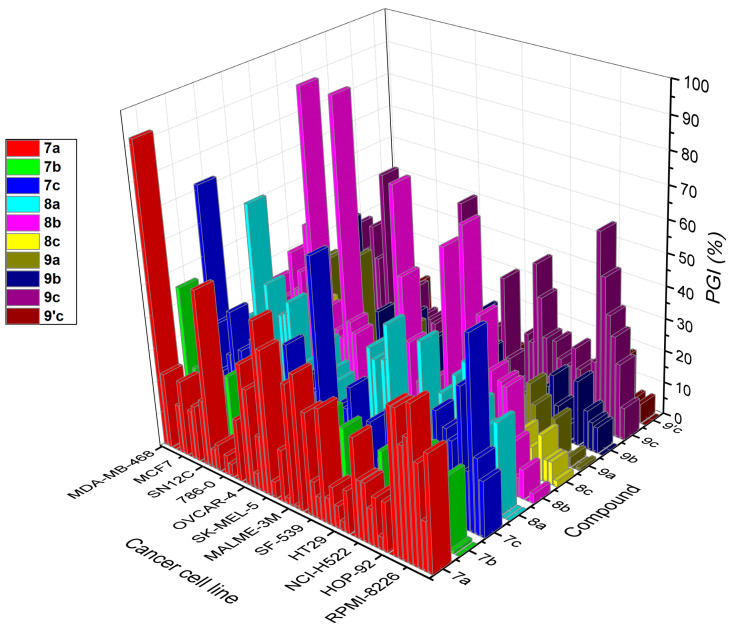
Anticancer activity of the benzo[*f*]quinolinium salts **7a–c** and pyrrolobenzo[*f*]quinoline cycloadducts **8a–c**, **9a–c**, and **9′c** against 60 NCI human cancer cell lines, expressed as the percentage growth inhibition.

In the same manner, the previously obtained benzo[*f*]quinoline derivatives **7a–c**, **8a–c**, **9a–c**, and **9′c** [13] were screened on the same 60 cancer cell lines in order to determine their anticancer activity. The obtained results are presented in Table 3 and Figure 2.

The results from Table 3 are presented in Figure 2.

Based on the results presented in Table 3 and Figure 2, it can be seen that the most active compound in the benzo[*f*]quinoline derivatives series is **8b**, with anticancer activity on most of the cell lines studied, showing 99% PGI on ovarian cancer on the OVCAR-4 cell line, and renal cancer on the ACHN cell line also has a 99% PGI value, and PGI values higher than 60% for three other cell lines.

In the benzo[*f*]quinolinium salts series, salt **7a** presents 92% PGI on breast cancer on the MDA-MB-468 cell line, while salt **7c** presents 72% PGI on the same cancer cell line and 63% PGI on the SK-MEL-5 melanoma cell line.

For prostate cancer, all compounds present some low or moderate activity on the PC-3 cell line (with PGI values between 3 and 57%), while for the DU-145 cell line, only compounds **7a** and **7b** show some low activity (PGI 5 to 7%).

In the case of leukemia, the most active compound is **9c** (with 61% PGI) on the SR cell line. In Non-Small Cell Lung Cancer, the most active compound is **8b** (with 74% PGI) on the NCI-460 cell line. In colon cancer, the most active compound is **8b** (with 66% PGI) on the HCT-116 cell line. In the case of CNS cancer, the most active compound is **8b** (with 79% PGI) on the U251 cell line. In melanoma, the most active compound is **7c** (with 63% PGI) on the SK-MEL-5 cell line. In ovarian cancer, the most active compound is **8b** (with 99% PGI) on the OVCAR-4 cell line. In renal cancer, the most active compound is **8b** (with 99% PGI) on the ACHN cell line. In prostate cancer, the most active compound is **9c** (with 57% PGI) on the PC-3 cell line. In breast cancer, the most active compounds are **7a** (with 92% PGI) and **7c** (with 72% PGI) on the MDA-MB-468 cell line and **8a** (with 65% PGI) on the MCF7 cell line.

**Scheme 4:**
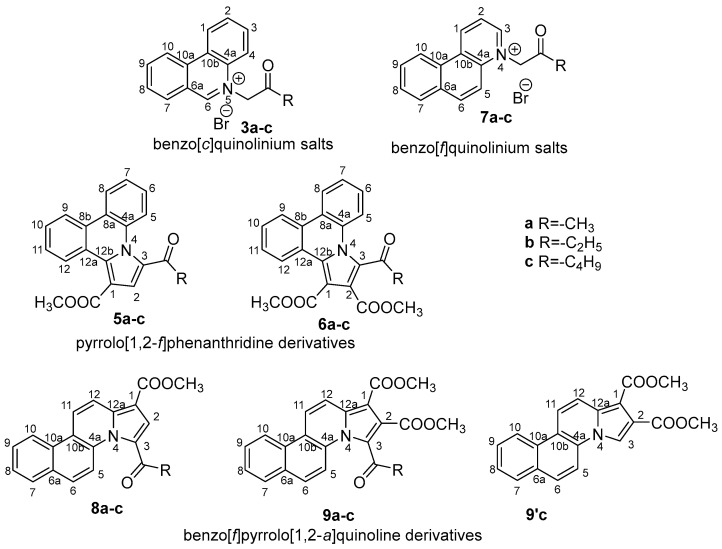
Ring numbering of the quinolinium derivatives.

In the benzo[*f*]quinoline derivatives series, the least active compound is **9′c** with a maximum of 18% PGI on some cancer cell lines. The lack of biological activity for the **9′c** compound could be explained by the absence of the keto group from the 3rd position of the benzo[*f*]pyrrolo [1,2-a]quinoline core (see Scheme 4).

Comparative study of the biological activity of benzo[*c*]quinolinium versus benzo[*f*]quinolinium derivatives shows that in the case of salts, benzo[*f*]quinolinium salts are much more active than benzo[*c*]quinolinium salts. Since both types of salts have the same solubility, the difference in biological activity must be attributed to structural differences. The transfer of the nitrogen atom from the middle ring (in the case of benzo[*c*]quinolinium salts) to the marginal ring (in the case of benzo[*f*]quinolinium salts) is shown to favor anticancer activity.

In leukemia, the compounds with the best anticancer activity are pyrrolo[1,2-f]phenanthridine derivatives. The best activity is shown by compound **6c**, which shows lethality in the SR leukemia cell line. Surprisingly, in the case of benzo[*f*]pyrrolo[1,2-*a*]quinolinyl derivatives, the same compound, type **9c**, derived from pivaloyl salt and DMAD, is the most active with maximum activity on the same cell line. This means that anticancer activity on this cell line requires the presence of both ester groups on the 1st and 2nd position as well as the pivaloyl group from the 3rd position.

Cycloadducts show specific anticancer activity on certain types of cancer cell lines. Different cancer cell line compounds with different structures show the best anticancer activity (on some cell lines, pyrrolo[1,2-*f*]phenanthridine cycloadducts are more active, while on other cell lines, benzo[*f*]pyrrolo[1,2-*a*]quinolinyl cycloadducts are more active). This non-selectivity in terms of the type of cancer cells on which a given compound is active explains the need to test as many compounds as possible on as many cancer cell lines as possible. For the cycloadducts that we tested, compounds **5a** and **6c** proved to be the most active on the studied cancer cell lines.

## 3. Materials and Methods

Materials and methods, general synthetic procedures, spectral characterization, and cell proliferation assay are widely presented in the supporting information.

## 4. Conclusions

In conclusion, we described the synthesis of a nine-novel benzo[*c*]quinoline derivatives and their anticancer efficacy. The pyrrolobenzo[*c*]quinoline cycloadducts were produced as a result of a two-step synthesis that involved a quaternization reaction and a [3+2] dipolar cycloaddition.

The simple and effective chemical method makes it possible to synthesize benzo[*c*]quinoline derivatives, which are otherwise difficult to obtain. A comparison of the effectiveness of synthesis using conventional thermal heating (TH), microwave heating (MW), and ultrasonic heating (US) has been carried out. It must be noted that the reaction setup under MW or US irradiation offers several additional benefits, including improved yields, a decrease in the amount of the utilized solvent compared to TH, a reduction in the reaction time from hours to minutes, and a reduction in the amount of energy consumed. Given the benefits listed above, it is possible to classify the obtaining reactions of ylides and their reaction with different dipolarophiles under MW or US irradiation as ecologically friendly.

The NCI used a single-dose experiment (10^−5^ M) to examine nine benzo[*c*]quinoline and ten benzo[*f*]quinoline compounds, including quaternary salts and cycloadducts, for potential anticancer characteristics. The cycloadduct **5a** was the most active compound, showing anticancer activity against the majority of the cell lines tested (with an excellent PGI in the area of 50–100%) and very good lethality against CNS cancer on the SNB-75 cell line (12%). Also, very good anticancer activity is shown by the compound **6c**, which shows significant lethality for the SR leukemia cell line (17%) and PGI values above 50% for eight cell lines.

According to the preliminary SAR correlations, cycloadducts **5a–c** and **6a–c** exhibit stronger anticancer activity than quaternary salts **3a–c.** The benzo[*f*]quinolinium salts **7a–c** are more active than benzo[*c*]quinolinium salts **5a–c**. The absence of the keto group from the 3rd position of the benzo[*f*]pyrrolo[1,2-*a*]quinoline core involves the loss of the biological activity of the **9′c** compound on almost all tested cancer cell lines. Anticancer activity on the SR leukemic cell line requires the presence of two ester groups in the 1st and 2nd positions and a pivaloyl group in the 3rd position, as evidenced by the increased biological activity of both cycloadducts **6c** and **9c** on this cell line. According to the increased anticancer activity of cycloadducts (compounds with a tetracyclic structure) compared to quaternary salts (compounds with a tricyclic structure), 4-fused cycles are preferred to 3-fused cycles. The cycloadducts **5a** and **6c** have promising anticancer activity and are potential candidates for further drug development. However, further studies remain to be performed in the cycloadducts class.

## Figures and Tables

**Table 1:** The synthesis of benzo[*c*]quinolinium derivatives under MW and US irradiation and conventional TH.

Compd	Microwaves (MW)		Ultrasounds (US)		Conventional TH	
	Reaction Time,	Yield, %	Reaction Time,	Yield, %	Reaction Time,	Yield, %
**3a**	10 min	91	20 min	87	36 h	67
**3b**	10 min	93	20 min	88	36 h	70
**3c**	10 min	94	20 min	90	36 h	72
**5a**	10 min	78	20 min	73	48 h	57
**5b**	10 min	80	20 min	75	48 h	59
**5c**	10 min	82	20 min	79	48 h	61
**6a**	15 min	73	30 min	68	48 h	51
**6b**	15 min	75	30 min	68	48 h	49
**6c**	15 min	76	30 min	71	48 h	46

**Table 2:** Single-dose (10^−5^ M) assay results of the benzo[*c*]quinolinium salts **3a–c** and pyrrolobenzo[*c*]quinoline cycloadducts **5a–c** and **6a–c** against 60 NCI human cancer cell lines, expressed as the percentage growth inhibition (PGI%).

Cell Type	Compound/Growth Inhibition Percent (PGI%) ^a^								
	3a	3b	3c	5a	5b	5c	6a	6b	6c
*Leukemia*									
CCRF–CEM	1	0	0	15	18	25	16	4	35
HL–60 (TB)	0	0	0	0	0	10	0	0	28
K–562	0	3	9	15	15	23	3	2	53
MOLT–4	0	1	0	27	43	33	27	19	55
RPMI–8226	0	1	4	13	15	30	13	12	53
SR	14	6	4	36	37	31	37	28	**100**(17) ^b^
*Non–small Cell Lung Cancer*									
A549/ATCC	5	0	0	4	0	0	0	0	0
EKVX	0	3	1	31	0	0	1	0	4
HOP–62	0	0	0	43	0	0	0	0	0
HOP–92	12	23	13	**72**	40	37	27	21	45
NCI–H226	3	0	0	39	10	0	19	5	15
NCI–H23	0	0	0	22	8	8	7	7	18
NCI–H322M	0	0	0	0	0	0	2	0	0
NCI–460	0	0	0	0	0	0	0	0	20
NCI–H522	0	0	0	14	0	0	0	0	27
*Colon Cancer*									
COLO 205	0	0	0	0	0	0	0	0	20
HCC–2998	0	0	0	9	0	0	0	0	15
HCT–116	0	0	0	50	17	12	12	7	41
HCT–15	0	0	0	29	28	35	15	11	54
HT29	0	0	0	8	7	1	5	0	29
KM12	1	0	0	15	13	21	4	0	38
SW–620	0	0	0	11	0	0	0	0	0
*CNS Cancer*									
SF–268	0	0	0	41	0	0	0	0	11
SF–295	2	0	1	44	8	7	9	3	19
SF–539	6	3	0	**72**	0	4	5	0	17
SNB–19	2	6	5	58	0	0	0	0	4
SNB–75	0	0	0	**100**(12) ^b^	0	16	0	0	17
U251	0	2	0	20	1	3	0	0	15
*Melanoma*									
LOX IMVI	0	3	0	36	15	9	9	7	19
MALME–3M	0	0	0	16	0	0	0	0	0
M14	0	0	0	28	8	4	5	2	26
MDA–MB–435	0	0	0	8	0	0	0	0	17
SK–MEL–2	0	0	0	1	0	7	0	0	17
SK–MEL–28	0	0	0	8	0	1	0	0	8
SK–MEL–5	0	0	0	**79**	11	12	12	4	54
UACC–257	0	0	0	0	0	0	0	0	6
UACC–62	7	5	2	33	38	34	37	37	50
*Ovarian Cancer*									
IGROV1	0	0	0	59	7	14	4	9	4
OVCAR–3	0	0	0	27	0	0	0	0	0
OVCAR–4	1	0	0	56	13	15	18	5	43
OVCAR–5	0	0	0	8	5	2	6	2	3
OVCAR–8	0	0	2	50	3	2	0	0	10
NCI/ADR–RES	0	0	0	56	12	11	8	0	17
SK–OV–3	0	0	0	1	0	0	0	0	0
*Renal Cancer*									
786–0	0	0	1	**64**	0	8	0	0	14
A498	0	0	0	0	0	0	0	0	0
ACHN	0	0	0	**64**	5	11	5	1	19
CAKI–1	10	11	7	56	30	40	21	20	40
RXF 393	10	7	14	**89**	10	9	8	3	18
SN12C	4	0	1	25	6	5	6	2	13
TK–10	0	2	1	26	0	0	0	0	0
UO–31	22	20	15	**63**	18	16	11	13	25
*Prostate Cancer*									
PC–3	7	7	8	44	49	41	31	28	**65**
DU–145	0	0	0	33	0	3	0	0	5
*Breast Cancer*									
MCF7	4	8	2	41	16	14	11	6	27
MDA–MB–231/ATCC	0	0	0	**67**	22	21	16	13	27
HS 578T	0	0	0	**71**	8	1	4	0	31
BT–549	0	0	0	**62**	0	0	0	0	38
T–47D	0	0	0	46	33	53	14	11	54
MDA–MB–468	11	24	2	35	10	12	7	5	46

^a^ Values are reported as one-dose assay, percentage growth inhibition (PGI), is growth relative to the no-drug control, and relative to the time zero number of cells; ^b^ cytotoxic effect; lethality percent is represented in brackets; the most active compounds are highlighted in bold.

**Table 3:** Single-dose (10^−5^ M) assay results of the benzo[*f*]quinolinium salts **7a–c** and pyrrolobenzo[*f*]quinoline cycloadducts **8a–c**, **9a–c**, and **9′c** against 60 NCI human cancer cell lines, expressed as the percentage growth inhibition (PGI%).

Cell Type	Compound/Growth Inhibition Percent (PGI%) ^a^									
	7a	7b	7c	8a	8b	8c	9a	9b	9c	9′c
*Leukemia*										
CCRF–CEM	35	0	17	0	3	2	1	0	10	0
HL–60 (TB)	15	1	23	0	0	7	0	8	23	6
K–562	31	22	58	28	9	7	3	9	31	4
MOLT–4	47	4	18	8	6	14	0	12	37	6
RPMI–8226	37	0	42	0	16	0	0	0	48	0
SR	34	1	9	24	32	9	13	20	**61**	18
*Non–small Cell Lung Cancer*										
A549/ATCC	44	23	25	31	33	0	0	0	21	0
EKVX	44	26	28	19	17	13	5	5	11	0
HOP–62	0	2	3	42	35	0	0	2	10	0
HOP–92	15	0	32	0	12	6	15	10	17	0
NCI–H226	4	0	3	37	50	17	21	19	23	0
NCI–H23	15	4	18	21	35	15	17	15	17	0
NCI–H322M	11	9	7	2	7	0	4	3	6	0
NCI–460	18	2	16	30	**74**	0	0	0	18	0
NCI–H522	18	7	30	9	0	4	4	5	23	0
*Colon Cancer*										
COLO 205	30	21	27	15	20	0	0	0	11	0
HCC–2998	0	0	6	8	1	0	0	0	23	0
HCT–116	13	0	8	43	**66**	2	11	0	35	2
HCT–15	3	0	0	10	9	9	6	3	45	5
HT29	7	0	26	0	27	1	0	0	20	4
KM12	12	4	12	6	16	6	0	0	22	0
SW–620	8	0	10	6	16	0	0	0	0	0
*CNS Cancer*										
SF–268	34	0	0	10	11	0	7	7	3	0
SF–295	7	23	20	10	23	5	3	6	4	0
SF–539	11	1	9	44	12	8	10	19	12	8
SNB–19	31	15	14	33	34	0	43	31	37	0
SNB–75	0	2	0	13	53	0	16	5	0	0
U251	41	25	28	32	**79**	0	16	0	12	0
*Melanoma*										
LOX IMVI	1	5	21	35	23	12	11	8	19	0
MALME–3M	37	1	22	11	29	8	7	10	16	7
M14	2	0	13	0	0	0	4	13	19	0
MDA–MB–435	16	0	0	1	7	0	0	2	7	2
SK–MEL–2	0	0	0	0	0	0	0	0	0	0
SK–MEL–28	5	0	7	5	1	0	2	3	10	0
SK–MEL–5	44	15	**63**	10	7	7	6	2	56	2
UACC–257	52	7	22	0	1	0	0	0	10	0
UACC–62	17	7	21	20	11	21	24	16	41	8
*Ovarian Cancer*										
IGROV1	5	0	5	23	30	6	0	0	1	0
OVCAR–3	30	0	24	3	33	0	17	0	0	0
OVCAR–4	37	0	18	30	**99**	6	0	12	12	0
OVCAR–5	0	0	4	5	27	0	15	0	7	0
OVCAR–8	19	15	33	21	29	0	10	21	14	0
NCI/ADR–RES	0	1	0	19	24	0	10	9	15	0
SK–OV–3	4	0	3	22	0	0	0	0	3	0
*Renal Cancer*										
786–0	6	7	5	0	34	0	11	0	15	2
A498	0	0	0	0	0	0	0	0	0	0
ACHN	8	0	0	39	**99**	2	4	4	23	0
CAKI–1	6	2	5	35	58	17	7	8	21	0
RXF 393	54	0	1	34	18	0	40	0	20	0
SN12C	20	23	30	25	43	4	15	18	12	0
TK–10	0	0	4	0	37	0	0	0	1	0
UO–31	17	16	21	42	48	28	15	13	11	18
*Prostate Cancer*										
PC–3	15	8	25	12	8	15	3	9	**57**	3
DU–145	0	0	0	5	7	0	0	0	0	0
*Breast Cancer*										
MCF7	23	9	36	**65**	20	33	14	11	29	10
MDA–MB–231/ATCC	15	0	22	24	31	20	36	9	39	0
HS 578T	0	9	11	18	37	6	36	44	16	0
BT–549	0	21	16	3	0	0	5	0	0	0
T–47D	23	5	30	15	25	12	7	11	29	0
MDA–MB–468	**92**	45	**72**	26	17	7	21	8	38	3

^a^ Values are reported as one-dose assay, percentage growth inhibition (PGI), is growth relative to the no-drug control, and relative to the time zero number of cells, the most active compounds are highlighted in bold.

## Data Availability

Data is contained within the article and supplementary material.

## Supplementary Materials

The following supporting information can be downloaded at: https://www.mdpi.com/article/10.3390/ph17010052/s1, Material and methods, general synthetic procedures, cell proliferation assay, spectral characterization, details of the NMR (^1^H NMR, ^13^C NMR), IR and HR-MS spectra of the synthesized compounds, along with a graphical representation of the anticancer activity of the tested compounds against NCI human cancer types can be found in the Supporting Information.
