# Supplementary material for: The Effective Synthesis of New Benzoquinoline Derivatives as Small Molecules with Anticancer Activity
Source: Pharmaceuticals (Basel). 2023 Dec 28;17(1):52. doi: 10.3390/ph17010052 (PMC10820420; doi:10.3390/ph17010052)
Supplement: Supplementary file 1 [file pharmaceuticals-17-00052-s001.zip › pharmaceuticals-2734821-supplementary.pdf]

Supporting Information for:

An Effective Synthesis of New Benzoquinoline Derivatives as Small Molecules with Anticancer Activity

Gheorghita Zbancioc [1,*], Ionel I. Mangalagiu [1,2] and Costel Moldoveanu [1,*]

[1]Chemistry Department, Alexandru Ioan Cuza University of Iasi, 11 Carol 1st Bvd, Iasi -700506, Romania

[2]Institute of Interdisciplinary Research- CERNESIM Centre, Alexandru Ioan Cuza University of Iasi, 11 Carol I, Iasi, 700506, Romania

*Corresponding authors

E-mail: gheorghita.zbancioc@uaic.ro (GZ) and costel.moldoveanu@uaic.ro (CM)

Contents

1. Materials and Methods 2

1.1. General Procedure for the Synthesis of Benzo[*c*]quinolinium Salts **3a–c** 2

1.2. General Procedure for Synthesis of Azatetracyclic Derivatives, **5a-c** and**6a-c** 3

2. Cell Proliferation Assay 6

3. NMR Spectra of the Obtained Compounds. 7

4. IR Spectra of the Obtained Compounds. .... **Error! Bookmark not defined.**

5. HR-MS Spectra of the Obtained Compounds 26

6. Graphical Representation for Anticancer Activity of the Obtained Compounds against Different Types of Cancer Cell Line 31

1. Materials and Methods

With the exception of bromoacetone, which was created by the reaction of acetone with bromine in the presence of acetic acid as a catalyst, all the chemicals and solvents were acquired from commercial sources and utilized without further purification. Melting points were uncorrected measurements made using an Electrothermal MEL-TEMP (Barnstead International, Dubuque, Iowa, USA) instrument in open capillary tubes. Commercial silica gel plates 60 F254 (Merck Darmstadt, Germany) were used for analytical thin-layer chromatography, and UV light was used to visualize the results. The NMR spectra were acquired using an Avance III 500 MHz spectrometer from Bruker Vienna, Austria, which operates at 500 MHz for ^{1}H and 125 MHz for ^{13}C.Chemical shift multiplicities were identified using the following abbreviations: s = singlet, d = doublet, dd = doublet of doublet, t = triplet, td = triplet of doublet, and m = multiplet. Chemical shifts were recorded using the delta (δ), part per million (ppm), and coupling constants (*J*) in Hz units. An Agilent Cary 630 FTIR (Agilent Technologies, Mulgrave, Australia) spectrophotometer was used to record IR data on powder in the diamond crystal ATR mode. Using a Bandelin Ultrasound reactor (Sonopuls GM 3200, Berlin, Germany) with a nominal power of 200 W and a frequency of 20 kHz, ultrasound-assisted reactions were performed. The ultrasonic converter was securely fastened to the booster horn SH 213 G. The booster horn was securely fastened using the titanium flat probe tip TT13 (diameter: 12.7 mm; length: 7 mm). The used solvent was submerged in the titanium probe tip. We were able to regulate the pulse sequence, amplitude (mean percent of the nominal power), and irradiation time using the employed reactor. All of these factors are anticipated to affect the response. 60% of the instrument's normal power and a 5s pulse on / 5s pulse off timing were used to perform the reactions. We employed a monomode reactor, the Monowave 300 (Anton Paar), for the microwave irradiation. A built-in IR sensor regulates the reactor's temperature, which can reach up to 300 °C. With the 'Ruby Thermometer,' it is also possible to measure internal temperature and IR simultaneously. Additional features of the Monowave 300 include its 850 W microwave power, 30 bar and 300 °C operating limits, Borosilicat reaction vial, 0.5–20 mL operating range, hydraulic pressure control, integrated magnetic stirrer agitation, and compressed air cooling. On a HESI-Orbitrap Exploris 120 Mass Spectrometer (Thermo Fisher, Walthan, MA, USA), HR-MS experiments were captured in positive mode. On a FlashSmart Elemental Analyzer CHNS/O (Thermo Fisher, Walthan, MA, USA), the elemental analysis was carried out.

1.1. General Procedure for the Synthesis of Benzo[*c*]quinolinium Salts **3a–c**

TH

To a solution of benzo[*c*]quinoline (0.896g, 5 mmol) in 20 mL of acetone, dropwise addition of halogenated derivatives **2a–c** (0.46 mL, 5.5 mmol of **2a** or 0,56 mL, 5.5 mmol of **2b** or 0.74 mL, 5.5 mmol of **2c**) with enhanced reactivity was performed. The resulting mixture was heated at reflux for 16 hours, followed by 20 hours of stirring at room temperature. After being filtered out, the quaternary salts **3a–c** (Scheme S1) that had precipitated were thrice washed with 5 mL of acetone before being vacuum-dried. No more purification was necessary.

MW

The mixture of reagents was placed in the reaction vessel (borosilicate), dissolved in 10 mL acetone, and subjected to microwave irradiation for 10 to 15 minutes (see Table 1). The best outcomes with MW irradiation came from a "temperature control" technique. A constant temperature (in this example 90 °C) is maintained while altering the power and pressure using the "temperature control" method. Three steps make up this process. By using the most power possible, the temperature is raised in the first step as soon as possible (in less than 1 minute). With the help of the magnetron power, the reaction mixture is maintained at a consistent temperature in the second stage. By ceasing the radiation and blowing the reaction vial with compressed air, the reaction tube is cooled to 55 °C in the final stage. The reaction vial is taken out of the reactor when the heating cycle is finished and processed according to instructions for TH.

US

The mixture of reagents was placed in the reaction vessel, dissolved in 10 mL acetone, and subjected to US irradiation (from 2 hours; see Table 1). After the irradiation cycle was finished, the reaction tube was taken out of the reactor and processed according to the above instructions for the TH condition.

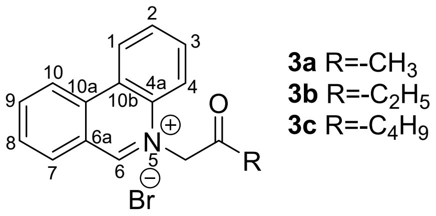

Scheme S1. General chemical structure of benzo[*c*]quinolinium salts **3a–c**.

5-(2-Oxopropyl)phenanthridin-5-ium bromide (**3a**). (1.059 g, 67% (under classical heating), 1.439 g, 91% (under microwave) and 1.375 g, 87% (under ultrasounds)) as a brown crystals, m.p.188–189 °C); **IR** (cm^{-1}): 3015, 3002 (C-H arom.), 2955 (C-H aliph.), 1731 (C=O, keto), 1627, 1602, 1476, 1448, 1349 (aromatic and heteroaromatic ring), 1168, 1036, 758, 743 (C–H and C=C bending); **^{1}H NMR** (500 MHz, DMSO-d6): δ 10.29 (1H, s, H-6), 9.20 (2H, m, overlapped peaks, H-7, H-4), 8.59 (1H, d, *J* = 8.0 Hz, H-1), 8.45 (2H, m, overlapped peaks, H-3, H-10), 8.10 (3H, m, overlapped peaks, H-2, H-8, H-9), 6.40 (2H, s, CH_2), 2.52 (3H, s, CH_3);**^{13}C NMR** (125 MHz, DMSO-d6): δ 199.8 (CO keto group), 156.6 (C-6), 138.7 (C-3), 134.7 (C-4a), 133.8 (C-10a), 133.1 (C-1), 132.1 (C-2), 130.7 (C-8), 130.4 (C-9), 125.4 (C-10b), 124.9 (C-7), 123.4 (C-4), 123.1 (C-6a), 120.1 (C-10), 65.5 (CH_2), 27.7 (CH_3). MS (ESI, acetonitrile (ACN), without hydrating water) Exact Mass for [M^+] ($C_{16}H_{14}NO$): 236.1075; found: 236.1068. Anal. calc. for $C_{16}H_{14}BrNO$ (316.20): C 60.78, H 4.46, N 4.43; found: C 60.73, H 4.42, N 4.39.

5-(2-Oxobutyl)phenanthridin-5-ium bromide (**3b**).(1.156 g, 70% (under classical heating), 1.536 g, 93% (under microwave) and 1.453 g, 88% (under ultrasounds)) as a yellow crystals, m.p. 213–214 °C); **IR** (cm^{-1}): 3011 (C-H arom.), 2970 (C-H aliph.), 1723 (C=O, keto), 1628, 1507, 1448, 1362 (aromatic and heteroaromatic ring), 1151, 1103, 978, 758, 721 (C–H and C=C bending); **^{1}H NMR** (500 MHz, DMSO-d6): δ 10.28 (1H, s, H-6), 9.20 (2H, m, overlapped peaks, H-7, H-4), 8.59 (1H, d, *J* = 8.0 Hz, H-1), 8.45 (2H, m, overlapped peaks, H-3, H-10), 8.11 (3H, m, overlapped peaks, H-2, H-8, H-9), 6.39 (2H, s, CH_2), 2.95 (2H, q, *J* = 7.5 Hz, CH_2 from ethyl group), 1.06 (3H, t, *J* = 7.5 Hz, CH_3 from ethyl group);**^{13}C NMR** (125 MHz, DMSO-d6): δ 202.4 (CO keto group), 156.6 (C-6), 138.7 (C-3), 134.6 (C-4a), 133.7 (C-10a), 133.1 (C-1), 132.2 (C-2), 130.7 (C-8), 130.4 (C-9), 125.4 (C-10b), 124.9 (C-7), 123.4 (C-4), 123.2 (C-6a), 120.1 (C-10), 64.9 (CH_2), 33.0 (CH_2 from ethyl group), 7.0 (CH_3 from ethyl group). MS (ESI, ACN, without hydrating water) Exact Mass for [M^+] ($C_{17}H_{16}NO$): 250.1232; found: 250.1222. Anal. calc. for $C_{17}H_{16}BrNO$ (330.23): C 61.83, H 4.88, N 4.24; found: C 61.77, H 4.83, N 4.20.

5-(3,3-Dimethyl-2-oxobutyl)phenanthridin-5-ium bromide (**3c**). (1.290 g, 72% (under classical heating), 1.684 g, 94% (under microwave) and 1.612 g, 90% (under ultrasounds)) as a yellow crystals, m.p. 236–237 °C); **IR** (cm^{-1}): 3056, 3019 (C-H arom.), 2920 (C-H aliph.), 1718 (C=O, keto), 1628, 1448, 1367, 1343, 1265 (aromatic and heteroaromatic ring), 1074, 1054, 1010, 784, 756, 713 (C–H and C=C bending); **^{1}H NMR** (500 MHz, MeOD-d4): δ 10.10 (1H, s, H-6), 9.08 (2H, m, overlapped peaks, H-7, H-4), 8.56 (1H, d, *J* = 8.0 Hz, H-1), 8.40 (1H, td, *J* = 7.5, 1.0 Hz, H-3) 8.10 (4H, m, overlapped peaks, H-2, H-8, H-9, H-10), 6.54 (2H, s, CH_2), 1.46 (9H, s, 3xCH_3 from *tert*-butyl group);**^{13}C NMR** (125 MHz, MeOD-d4): δ 207.4 (CO keto group), 158.0 (C-6), 140.1 (C-3), 134.8 (C-4a), 135.3 (C-10a), 134.2 (C-1), 133.5 (C-2), 131.8 (C-8), 131.7 (C-9), 127.4 (C-10b), 126.1 (C-7), 125.1 (C-6a), 124.4 (C-4), 120.1 (C-10), 63.6 (CH_2), 26.5 (3xCH_3 from *tert*-butyl group). MS (ESI, ACN, without hydrating water) Exact Mass for [M^+] ($C_{19}H_{20}NO$): 278.1545; found: 278.1535. Anal. calc. for $C_{19}H_{20}BrNO$ (358.28): C 63.70, H 5.63, N 3.91; found: C 63.65, H 5.58, N 3.86.

1.2. General Procedure for Synthesis of Azatetracyclic Derivatives, **5a-c** and**6a-c**

TH

40 mL of 1,2-butylene oxide were used to suspend a combination of the benzo[*c*]quinolinium salt **3a-c** (0.791 g, 2.5 mmol of **3a** or 0,826 g, 2.5 mmol of **3b** or 0.896 g, 2.5 mmol of **3c**) and either methyl propiolate (0.31 mL, 3.5 mmol) or dimethyl acetylenedicarboxylate (0.43 mL, 3.5 mmol). For 48 hours, the refluxing and stirring was maintained. Following completion of the reaction (TLC), the resulting solution was cooled to room temperature and evaporated under reduced pressure to produce the crude product. By purifying the crude product using column chromatography on silica gel (eluted with 99.5/0.5 CH_2Cl_2/CH_3OH), the required cycloadducts **5a-c** and **6a-c** (Scheme S2) were obtained.

MW

The reaction vessel was made of borosilicate, and microwave irradiation was applied for 10 to 15 minutes (see Table 1) to the mixture of the reagents benzo[*c*]quinolinium salt **3a-c** (0.791 g, 2.5 mmol of **3a** or 0,826 g, 2.5 mmol of **3b** or 0.896 g, 2.5 mmol of **3c**) and methyl propiolate (0.31 mL, 3.5 mmol) or dimethyl acetylenedicarboxylate (0.43 mL, 3.5 mmol). The best outcomes with MW irradiation came from a "temperature control" technique. By adjusting the power and pressure while maintaining a constant temperature (in this case, 130 °C), the "temperature control" method is effective. The reaction vial is taken out of the reactor when the heating cycle is finished and processed according to instructions for TH.

US

The reaction vessel containing the mixture of the reagents benzo[*c*]quinolinium salt **3a-c** (0.791 g, 2.5 mmol of **3a** or 0,826 g, 2.5 mmol of **3b** or 0.896 g, 2.5 mmol of **3c**) and either methyl propiolate (0.31 mL, 3.5 mmol) or dimethyl acetylenedicarboxylate (0.43 mL, 3.5 mmol) was placed under US irradiation, and the mixture was subjected to radiation for 20 to 30 minutes (see Table 1). Following the completion of the irradiation cycle, the reaction tube was taken out of the reactor and processed as described above for the TH condition.

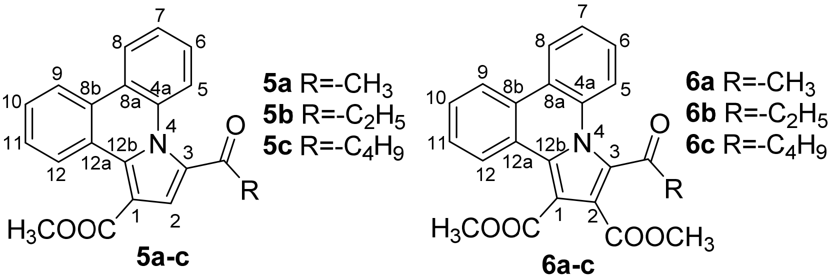

Scheme S2. General chemical structure of pyrrolobenzo[*c*]quinoline derivatives **5a–c** and **6a-c**.

Methyl 3-acetylpyrrolo[1,2-f]phenanthridine-1-carboxylate (**5a**). (0.452 g, 57% (under classical heating), 0.619 g, 78% (under microwave) and 0.579 g, 73% (under ultrasounds)) as a beige crystals, m.p.176–177 °C); **IR** (cm^{-1}): 3071 (C-H arom.), 2950, 2927 (C-H aliph.), 1718 (C=O, ester), 1654 (C=O, keto), 1524, 1438, 1367, 1319 (aromatic and heteroaromatic ring), 1271, 1228, 1177, 1121, 1090, 1074, 1054 (C–O–C, ester), 931, 741, 713 (C–H and C=C bending); **^{1}H NMR** (500 MHz, $CDCl_3$): δ 9.55 (1H, d, *J* = 7.5 Hz, H-5) 8.27 (2H, m, overlapped peaks, H-8, H-12), 7.96 (1H, s, H-2), 7.10 (1H, d, *J* = 8.5 Hz, H-9), 7.58 (2H, m, overlapped peaks, H-6, H-7), 7.42 (2H, m, overlapped peaks, H-10, H-11), 3.95 (3H, s, CH_3 of methoxycarbonyl group from 1st position), 2.67 (3H, s, CH_3 of acetyl group from 3rd position);**^{13}C NMR** (125 MHz, $CDCl_3$): δ 187.5 (CO keto group from 3rd position), 165.0 (CO keto ester from 1st position), 139.3 (C-12b), 131.8 (C-8b), 129.6 (C-6), 129.5 (C-3), 129.4 (C-2), 128.7 (C-4a), 128.3 (C-5), 128.1 (C-7), 127.7 (C-10), 125.7 (C-11), 124.1 (C-8a), 123.5 (C-12), 123.1 (C-12a), 122.1 (C-8), 122.0 (C-9), 110.4 (C-1), 52.0 (CH_3 of methoxycarbonyl group from 1st position), 28.2 (CH_3 of acetyl group from 3rd position).MS (ESI, MeOH-ACN, without hydrating water) Exact Mass for [M+Na] $C_{20}H_{15}NO_3Na$: 340.0950; found: 340.0944. Anal. calc. for $C_{20}H_{15}NO_3$ (317.34): C 75.70, H 4.76, N 4.41; found: C 75.64, H 4.73, N 4.36.

Methyl 3-propionylpyrrolo[1,2-f]phenanthridine-1-carboxylate (**5b**). (0.489 g, 59% (under classical heating),0.663 g, 80% (under microwave)and 0.621 g, 75% (under ultrasounds)) as a beige crystals, m.p.132–133 °C); **IR** (cm^{-1}): 3080, 3071 (C-H arom.), 2985, 2946 (C-H aliph.), 1718 (C=O, ester), 1656 (C=O, keto), 1528, 1438, 1373, 1312 (aromatic and heteroaromatic ring), 1224, 1168, 1123, 1097 (C–O–C, ester), 916, 739, 715 (C–H and C=C bending);**^{1}H NMR** (500 MHz, $CDCl_3$): δ 9.43 (1H, dd, *J* = 7.0, 2.0 Hz, H-5) 8.04 (2H, m, overlapped peaks, H-8, H-12), 7.81 (1H, s, H-2), 7.46 (1H, dd, *J* = 7.5, 1.0 Hz, H-9), 7.41 (2H, m, overlapped peaks, H-6, H-7), 7.25 (2H, m, overlapped peaks, H-10, H-11), 3.89 (3H, s, CH_3 of methoxycarbonyl group from 1st position), 2.94 (2H, q,*J* = 7.5 Hz, CH_2 of propionyl group from 3rd position), 1.29 (3H, t, *J* = 7.5 Hz, CH_3 of propionyl group from 3rd position);**^{13}C NMR** (125 MHz, $CDCl_3$): δ 191.1 (CO keto group from 3rd position), 164.7 (CO keto ester from 1st position), 138.7 (C-12b), 131.4 (C-8b), 129.1 (C-6), 128.9 (C-3), 128.3 (C-4a), 128.2 (C-2), 127.9 (C-5), 127.7 (C-7), 127.3 (C-10), 125.3 (C-11), 123.8 (C-8a), 123.1 (C-12), 122.7 (C-12a), 121.7 (C-8), 121.5 (C-9),

110.0 (C-1), 51.7 (CH_3 of methoxycarbonyl group from 1st position), 33.3 (CH_2 of propionyl group from 3rd position), 9.3 (CH_3 of propionyl group from 3rd position). MS (ESI, MeOH-ACN, without hydrating water) Exact Mass for [M+Na] $C_{21}H_{17}NO_3Na$: 354.1106; found: 354.1100. Anal. calc. for $C_{21}H_{17}NO_3$ (331.37): C 76.12, H 5.17, N 4.23; found: C 76.08, H 5.14, N 4.18.

Methyl 3-pivaloylpyrrolo[1,2-f]phenanthridine-1-carboxylate (**5c**).(0.548 g, 61% (under classical heating), 0.737 g, 82% (under microwave) and 0.710 g, 79% (under ultrasounds)) as a beige crystals, m.p.139–140 °C); **IR** (cm^{-1}): 3030 (C-H arom.), 2974, 2952 (C-H aliph.), 1710 (C=O, ester), 1647 (C=O, keto), 1528, 1438, 1317(aromatic and heteroaromatic ring), 1216, 1157, 1120, 1097 (C–O–C, ester), 918, 747, 719 (C–H and C=C bending);**^{1}H NMR** (500 MHz, $CDCl_3$): δ 9.51 (1H, dd, *J* = 8.0, 1.0 Hz, H-5) 8.07 (2H, m, overlapped peaks, H-8, H-12), 7.75 (1H, s, H-2), 7.45 (1H, td, *J* = 8.0, 1.0 Hz, H-7), 7.40 (1H, td, *J* = 8.0, 1.0 Hz, H-6), 7.26 (3H, m, overlapped peaks, H-9, H-10, H-11), 3.94 (3H, s, CH_3 of methoxycarbonyl group from 1st position), 1.50 (9H, s, 3xCH_3 of pivaloyl group from 3rd position);**^{13}C NMR** (125 MHz, $CDCl_3$): δ 200.4 (CO keto group from 3rd position), 165.0 (CO keto ester from 1st position), 137.0 (C-12b), 131.6 (C-8b), 128.9 (C-6), 127.8 (C-3), 127.7 (C-7), 127.7 (C-5), 127.6 (C-10), 127.6 (C-4a), 125.0 (C-11), 124.1 (C-2), 124.1 (C-8a), 123.6 (C-12), 122.7 (C-12a), 121.7 (C-8), 120.6 (C-9), 109.6 (C-1), 51.7 (CH_3 of methoxycarbonyl group from 1st position), 44.3 (C of pivaloyl group from 3rd position), 28.7 (3xCH_3 of pivaloyl group from 3rd position). MS (ESI, MeOH-ACN, without hydrating water) Exact Mass for [M+Na] $C_{23}H_{21}NO_3Na$: 382.1419; found: 382.1411. Anal. calc. for $C_{23}H_{21}NO_3$ (359.43): C 76.86, H 5.89, N 3.90; found: C 76.80, H 5.86, N 3.86.

Dimethyl 3-acetylpyrrolo[1,2-f]phenanthridine-1,2-dicarboxylate (**6a**). (0.479 g, 51% (under classical heating), 0.685 g, 73% (under microwave) and 0.638 g, 68% (under ultrasounds)) as abeige crystals, m.p. 171–172 °C); **IR** (cm^{-1}): 3002 (C-H arom.), 2994, 2950 (C-H aliph.), 1735,1712 (C=O, ester), 1695 (C=O, keto), 1485, 1457, 1446, 1377, 1356 (aromatic and heteroaromatic ring), 1261, 1215, 1172, 1162, 1138, 1066 (C–O–C, ester), 972, 846, 754, 739 (C–H and C=C bending);**^{1}H NMR** (500 MHz, $CDCl_3$): δ 8.27 (2H, m, overlapped peaks, H-5, H-12), 8.20 (1H, d, *J* = 7.0 Hz, H-8), 8.46 (5H, m, overlapped peaks, H-6, H-7, H-9, H-10, H-11), 4.02 (3H, s, CH_3 of methoxycarbonyl group from 1st position), 3.91 (3H, s, CH_3 of methoxycarbonyl group from 2nd position), 2.65 (3H, s, CH_3 of acetyl group from 3rd position);**^{13}C NMR** (125 MHz, $CDCl_3$): δ 195.6 (CO keto group from 3rd position), 166.9 (CO keto ester from 2nd position), 164.3 (CO keto ester from 1st position), 131.5 (C-12b), 130.8 (C-8b), 129.8 (C-2), 128.8 (C-6), 128.7 (C-7), 128.6 (C-10), 126.7 (C-4a), 126.1 (C-11), 124.8 (C-8), 124.4 (C-12), 124.0 (C-3), 123.3 (C-8a), 122.6 (C-5), 120.3 (C-12a), 118.8 (C-9), 111.0 (C-1), 52.9 (CH_3 of methoxycarbonyl group from 1st position), 52.6 (CH_3 of methoxycarbonyl group from 2nd position), 32.0 (CH_3 of acetyl group from 3rd position). MS (ESI, MeOH-ACN, without hydrating water) Exact Mass for [M+Na] $C_{22}H_{17}NO_5Na$: 398.1004; found: 398.0997. Anal. calc. for $C_{22}H_{17}NO_5$ (375.38): C 70.39, H 4.56, N 3.73; found: C 70.34, H 4.51, N 3.69.

Dimethyl 3-propionylpyrrolo[1,2-f]phenanthridine-1,2-dicarboxylate (**6b**). (0.477 g, 49% (under classical heating), 0.730 g, 75% (under microwave) and 0.6662 g, 68% (under ultrasounds)) as abeige crystals, m.p.176–177 °C); **IR** (cm^{-1}): 3093, 3078 (C-H arom.), 2987, 2953 (C-H aliph.), 1710 (C=O, ester), 1654 (C=O, keto), 1520, 1483, 1440, 1407, 1360 (aromatic and heteroaromatic ring), 1244, 1220, 1127, 1114, 1097 (C–O–C, ester), 933, 754, 741, 713 (C–H and C=C bending);**^{1}H NMR** (500 MHz, $CDCl_3$): δ 8.19 (1H, d, *J* = 5.0 Hz, H-12), 8.12 (2H, m, overlapped peaks, H-5, H-8), 7.44 (2H, m, overlapped peaks, H-6, H-7), 7.35 (3H, m, overlapped peaks, H-9, H-10, H-11), 4.02 (3H, s, CH_3 of methoxycarbonyl group from 1st position), 3.88 (3H, s, CH_3 of methoxycarbonyl group from 2nd position), 2.88 (2H, q, *J* = 7.0 Hz, CH_2 of propionyl group from 3rd position), 1.29 (3H, t, *J* = 7.0 Hz, CH_3 of propionyl group from 3rd position);**^{13}C NMR** (125 MHz, $CDCl_3$): δ 200.2 (CO keto group from 3rd position), 167.3 (CO keto ester from 2nd position), 164.0 (CO keto ester from 1st position), 131.5 (C-12b), 131.1 (C-8b), 128.7 (C-6), 128.6 (C-2), 128.6 (C-7), 128.4 (C-10), 126.3 (C-4a), 126.0 (C-11), 124.3 (C-12), 124.3 (C-8), 124.0 (C-3), 123.2 (C-8a), 122.5 (C-5), 118.6 (C-12a), 118.0 (C-9), 111.1 (C-1), 53.0 (CH_3 of methoxycarbonyl group from 1st position), 52.4 (CH_3 of methoxycarbonyl group from 2nd position), 38.3 (CH_2 of propionyl group from 3rd position), 8.2 (CH_3 of propionyl group from 3rd position). MS (ESI, MeOH-ACN, without hydrating water) Exact Mass for [M+Na] $C_{23}H_{19}NO_5Na$: 412.1161; found: 412.1154. Anal. calc. for $C_{23}H_{19}NO_5$ (389.41): C 70.94, H 4.92, N 3.60; found: C 70.88, H 4.88, N 3.56.

Dimethyl 3-pivaloylpyrrolo[1,2-f]phenanthridine-1,2-dicarboxylate (**6c**). (0.480 g, 46% (under classical heating), 0.793 g, 76% (under microwave) and 0.741 g, 71% (under ultrasounds)) as abeige crystals, m.p. 128–129 °C); **IR** (cm^{-1}): 3067, 3035 (C-H arom.), 2987, 2955 (C-H aliph.), 1723, 1707 (C=O, ester), 1692 (C=O, keto), 1496, 1455, 1438, 1414, 1360 (aromatic and heteroaromatic ring), 1280, 1263, 1218, 1129, 1110, 1021 (C–O–C, ester), 933, 760, 747, 717 (C–H and C=C bending);**^{1}H**

NMR (500 MHz, $CDCl_3$): δ 8.20 (1H, d, *J* = 8.0 Hz, H-12), 8.15 (1H, dd, *J* = 7.0, 3.0 Hz, H-5), 8.06 (1H, dd, *J* = 7.0, 3.0 Hz, H-8), 7.61 (1H, d, *J* = 7.5 Hz, H-9), 7.45 (2H, m, overlapped peaks, H-6, H-7), 7.36 (2H, m, overlapped peaks, H-10, H-11), 4.04 (3H, s, CH_3 of methoxycarbonyl group from 1st position), 3.83 (3H, s, CH_3 of methoxycarbonyl group from 2nd position), 1.08 (9H, s, 3xCH_3 of pivaloyl group from 3rd position);^{13}**C NMR** (125 MHz, $CDCl_3$): δ 208.5 (CO keto group from 3rd position), 167.7 (CO keto ester from 2nd position), 163.4 (CO keto ester from 1st position), 132.7 (C-12b), 129.5 (C-8b), 128.9 (C-6), 128.5 (C-10), 128.2 (C-7), 127.4 (C-2), 126.4 (C-11), 126.1 (C-4a), 124.2 (C-12), 124.2 (C-3), 124.0 (C-8), 123.1 (C-8a), 122.6 (C-5), 118.6 (C-9), 116.9 (C-12a), 111.7 (C-1), 53.0 (CH_3 of methoxycarbonyl group from 1st position), 52.0 (CH_3 of methoxycarbonyl group from 2nd position), 47.5 (C of pivaloyl group from 3rd position), 27.6 (3xCH_3 of pivaloyl group from 3rd position). MS (ESI, MeOH-ACN, without hydrating water) Exact Mass for [M+Na] $C_{25}H_{23}NO_5Na$: 440.1474; found: 440.1465. Anal. calc. for $C_{25}H_{23}NO_5$ (417.46): C 71.93, H 5.55, N 3.36; found: C 71.87, H 5.51, N 3.31.

2. Cell Proliferation Assay

The National Cancer Institute (NCI, Bethesda, MD, USA) carried out the in vitro biological studies as part of the Developmental Therapeutics Program (DTP).

The Developmental Therapeutics Program (DTP) at NCI has successfully guided applications of late-stage preclinical therapeutics through the pivotal stages of development for more than 50 years. As a result, this initiative was successful in finding and developing more than 70% of the anticancer medications used in modern therapy [51].

This screen works with 60 different human tumor cell lines that represent leukemia, melanoma, and malignancies of the ovary, breast, prostate, kidney, colon, and brain [52]. Prioritizing synthetic chemicals or natural product samples that selectively inhibit the growth or kill certain tumor cell lines is the goal for further assessment. This screen is distinctive in that the biological response pattern generated by a specific compound's complex dosage response in 60-cell lines can be used by the COMPARE program in pattern recognition algorithms [40].

The evaluation of all substances against the 60 cell lines at a single dose of 10^{-5} M constitutes the first screening stage. The single-dose screen's result is shown as a mean graph, and the COMPARE application can be used to analyze it.

The Standard NCI/DTP Methodology of the In Vitro Cancer Screen

In an RPMI (Roswell Park Memorial Institute) 1640 medium made up of 5% fetal bovine serum and 2 × 10^{-3} M L-glutamine, all 60 human tumor cell lines of malignancy were cultured. For a standard screening test, 100 µL of cancer cells are injected into 96-well microtiter plates (at plating densities ranging from 5000 to 40,000 cells/well depending on the rate of cell division of certain cell lines). Following cell inoculation, the microtiter plates are incubated under particular conditions for 24 hours before the addition of experimental drugs: 37°C, 5% CO2, 95% air, and 100% relative humidity. Two plates for each cell line are fixed in situ with TCA after 24 hours to establish measurements of each cell population at the time the testing substance (Tz) was added. Before usage, the testing chemicals are frozen after being solubilized in DMSO (dimethyl sulfoxide) at 400 times the intended final maximum test concentration. After the testing ingredient has been added, a portion of the frozen concentrate is defrosted, diluted by two, and then mixed with a medium containing 50 µg/mL gentamicin. The required final drug concentration (10^{-5} M) is achieved by adding 100 µL of the testing compound to the microtiter wells that already contain 100 µL of the medium. The microtiter plates are incubated for a further 48 hours under the previously described conditions after adding the testing chemical. The addition of cold TCA completes the test in the case of adherent cells. By adding 50 µL of cold, 50% (w/v) TCA (final concentration, 10% TCA), the cancer cells are fixed in place. This is followed by a 60-minute incubation period at 4 °C. The plates are air dried after being rinsed five times with tap water and the supernatant is discarded. After the plates have dried, 100 µL of a fluorescent dye solution (sulforhodamine B) is added to each well. The plates are then incubated at 25 °C for 10 min. The plates are washed five times with a 1% acetic acid solution to remove the unbound fluorescent dye, and then they are dried in the air. A 10 × 10^{-3} Mtrizma base is used to solubilize the bound fluorescent dye, and an automated plate reader is used to detect the absorbance at 515 nm. The procedure is the same as for suspension cells, with the exception that settled cells are fixed in the bottom of the wells by adding 50 µL of TCA 80% solution (final concentration, 16% TCA) to the test.

Calculating the percentage growth inhibition (PGI) using the seven absorbance readings is as follows: $[(Ti - Tz)/(C - Tz)] \times 100$ (for concentrations for which Ti>/= Tz);

$[(Ti - Tz)/Tz] \times 100$ (for concentrations for which Ti<Tz).

Where Ti = test growth in the presence of the drug at the concentration level, C = control growth, and Tz = time zero.

Three dose-response parameters are derived for each tested chemical.

3. NMR Spectra of the Obtained Compounds

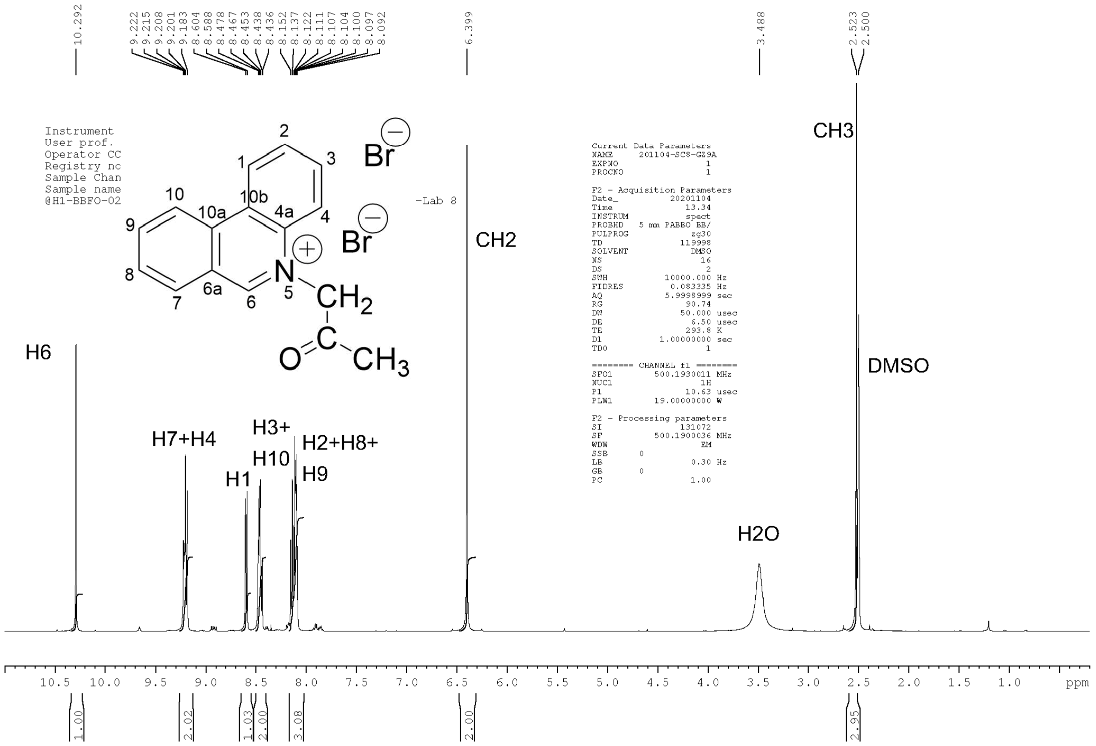

S1a Fig. ^{1}H NMR spectrum of the compound **3a** in DMSO at 500 MHz.

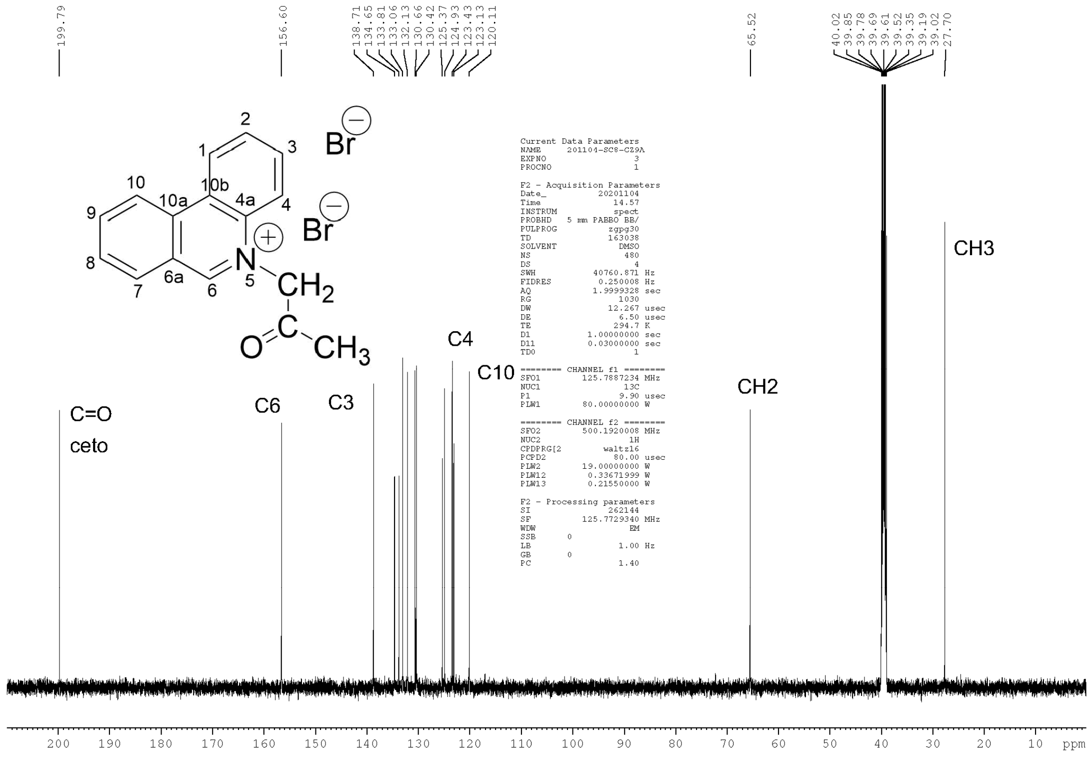

S1b Fig. ^{13}C NMR spectrum of the compound **3a** in DMSO at 125 MHz.

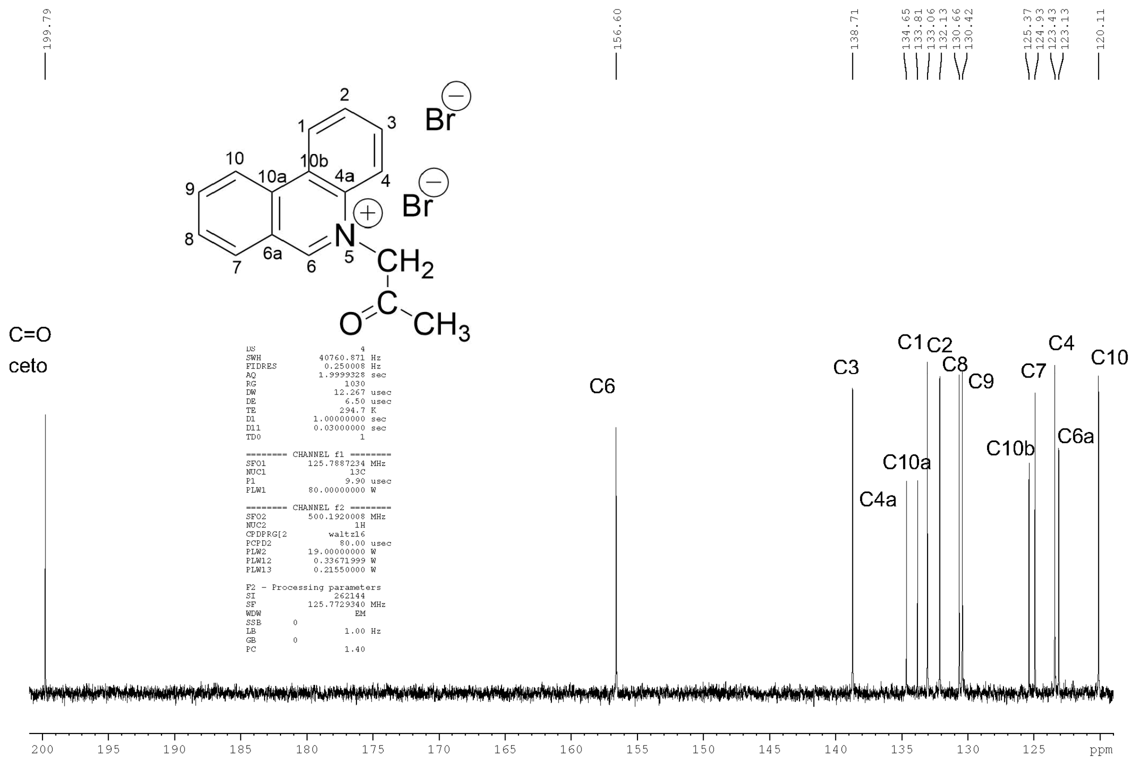

S1c Fig. Detail in the aromatic area of the ^{13}C NMR spectrum of the compound **3a** in DMSO at 125 MHz.

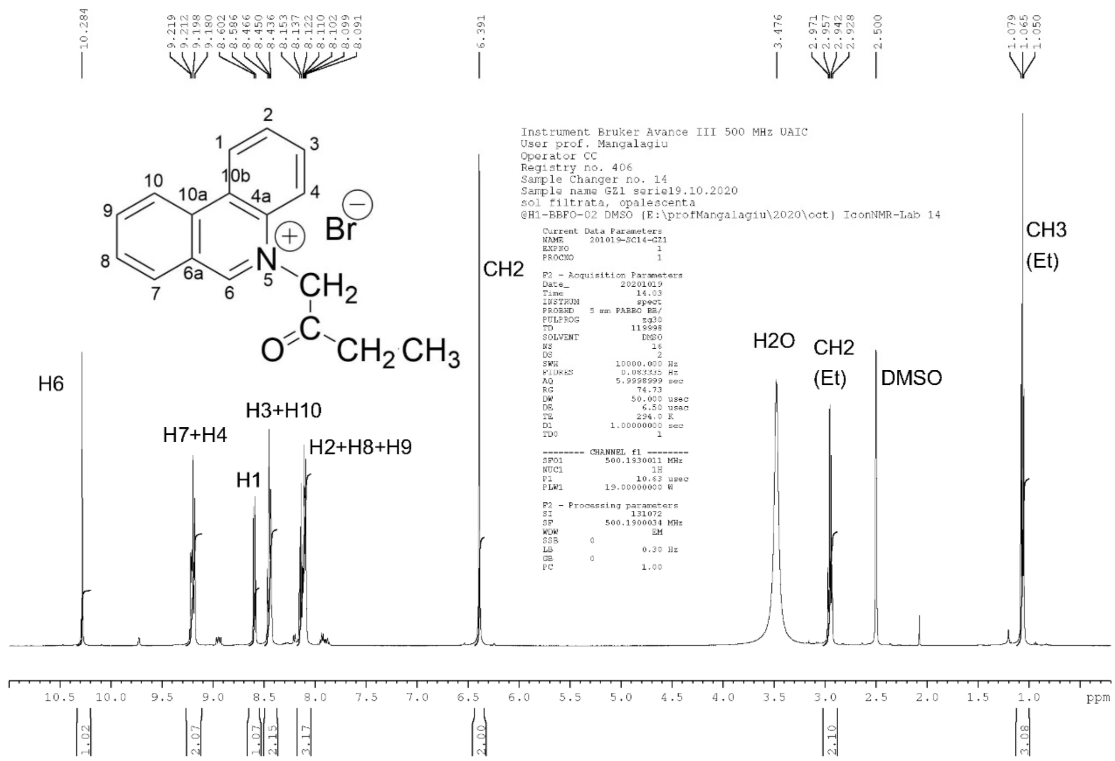

S2a Fig. ^{1}H NMR spectrum of the compound **3b** in DMSO at 500 MHz.

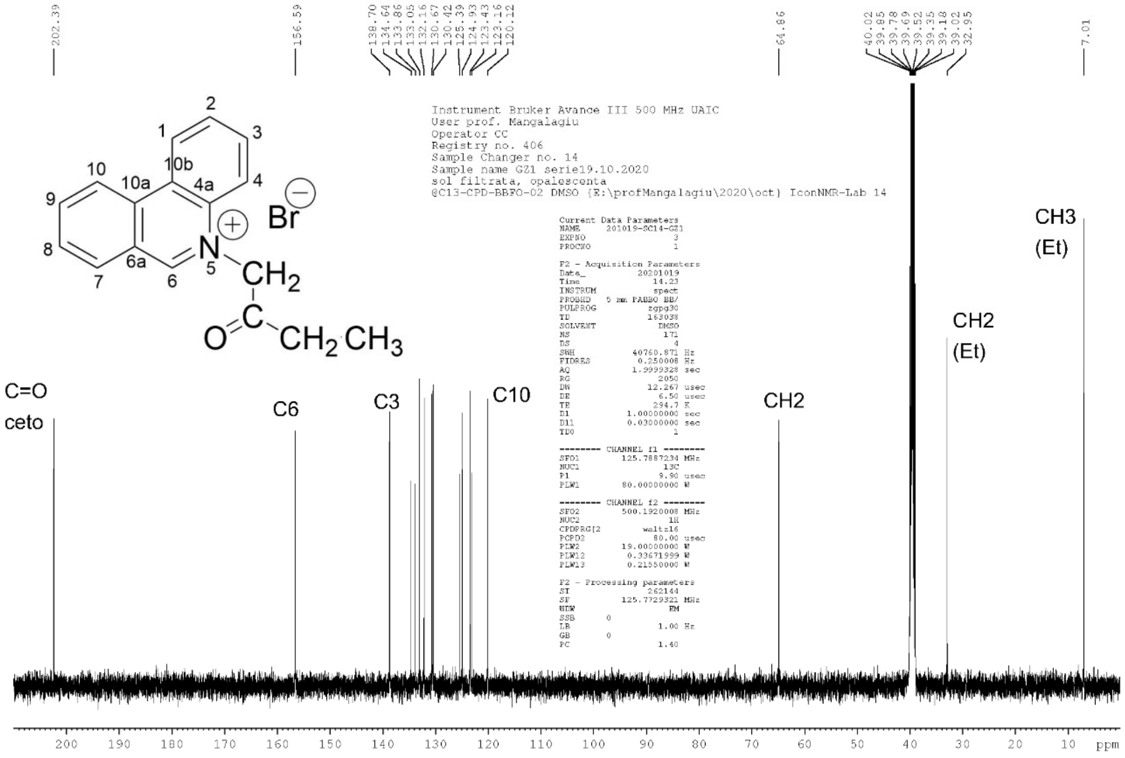

S2b Fig. ^{13}C NMR spectrum of the compound **3b** in DMSO at 125 MHz.

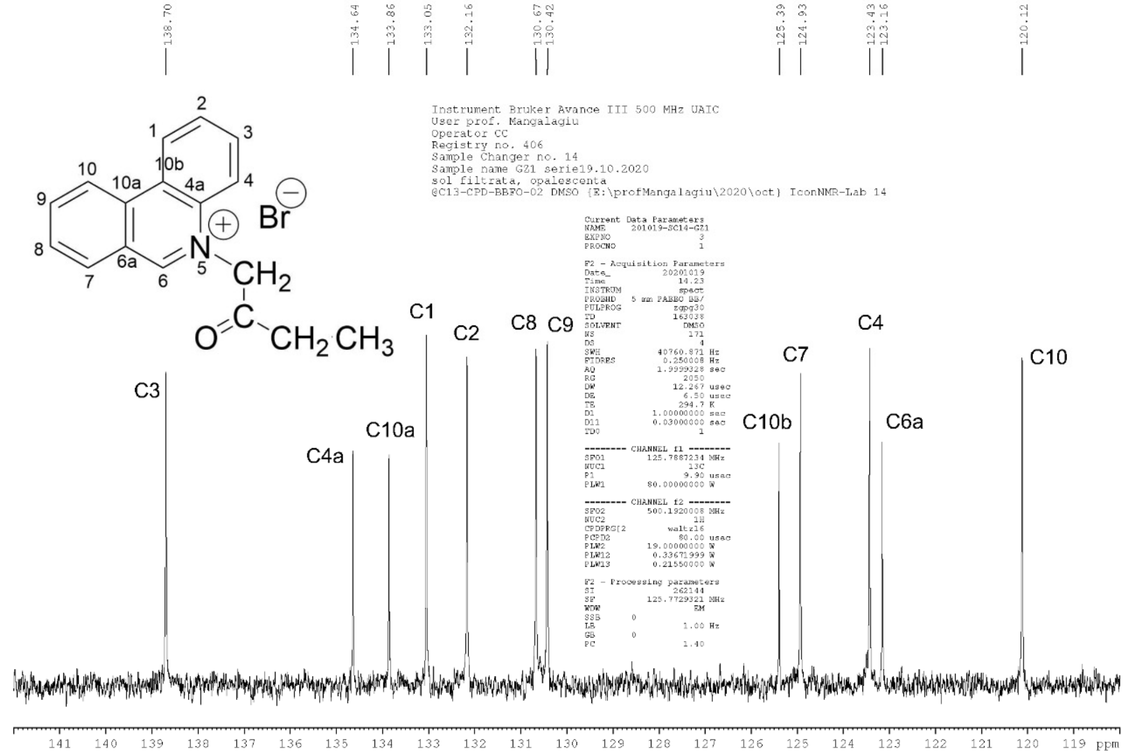

S2c Fig. Detail in the aromatic area of the ^{13}C NMR spectrum of the compound **3b** in DMSO at 125 MHz.

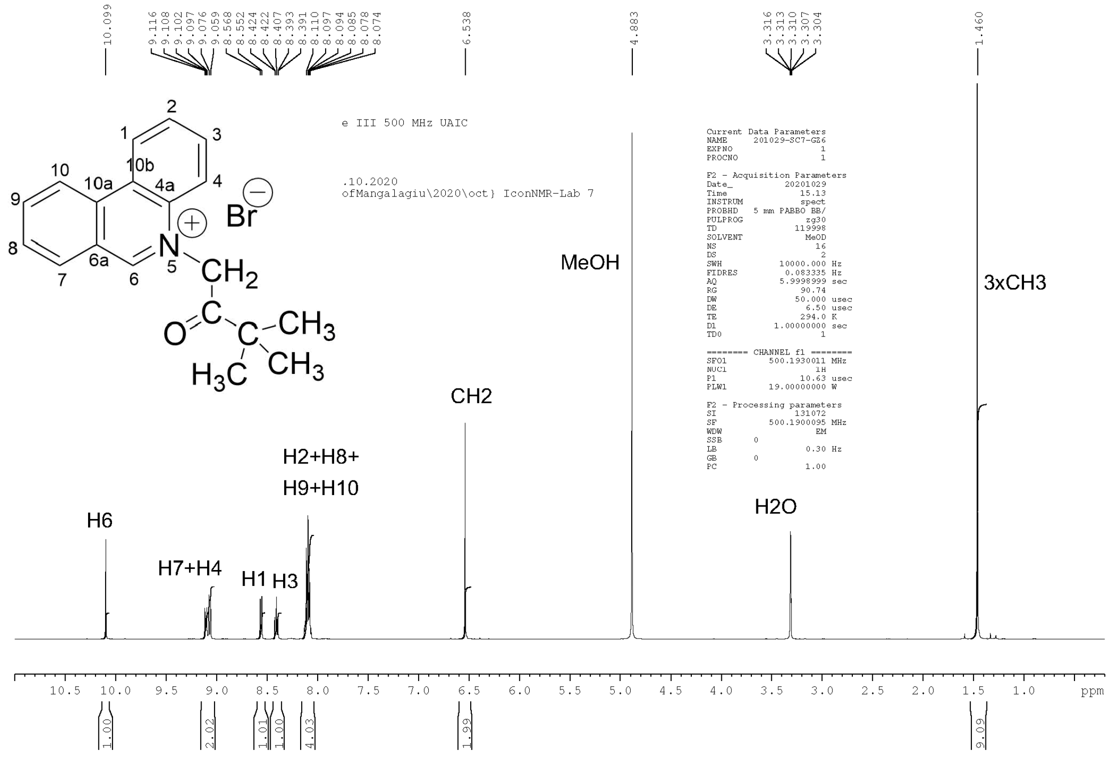

S3a Fig. ^{1}H NMR spectrum of the compound **3c** in MeOD at 500 MHz.

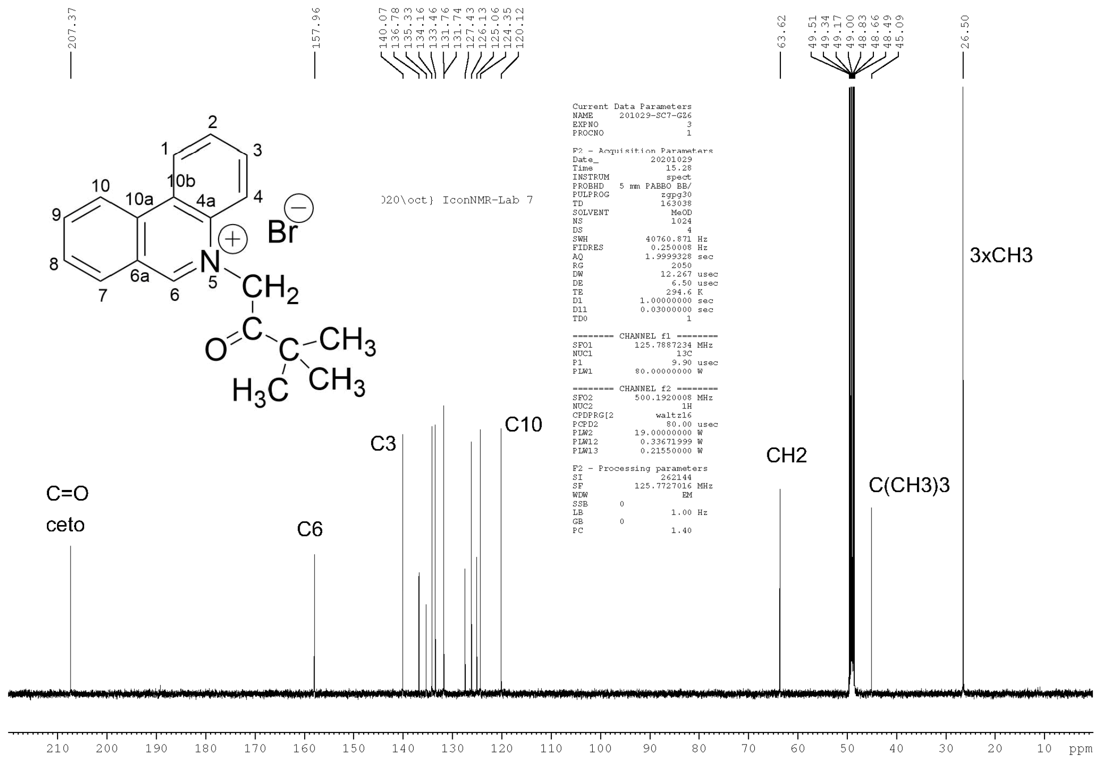

S3b Fig. ^{13}C NMR spectrum of the compound **3c** in MeOD at 125 MHz.

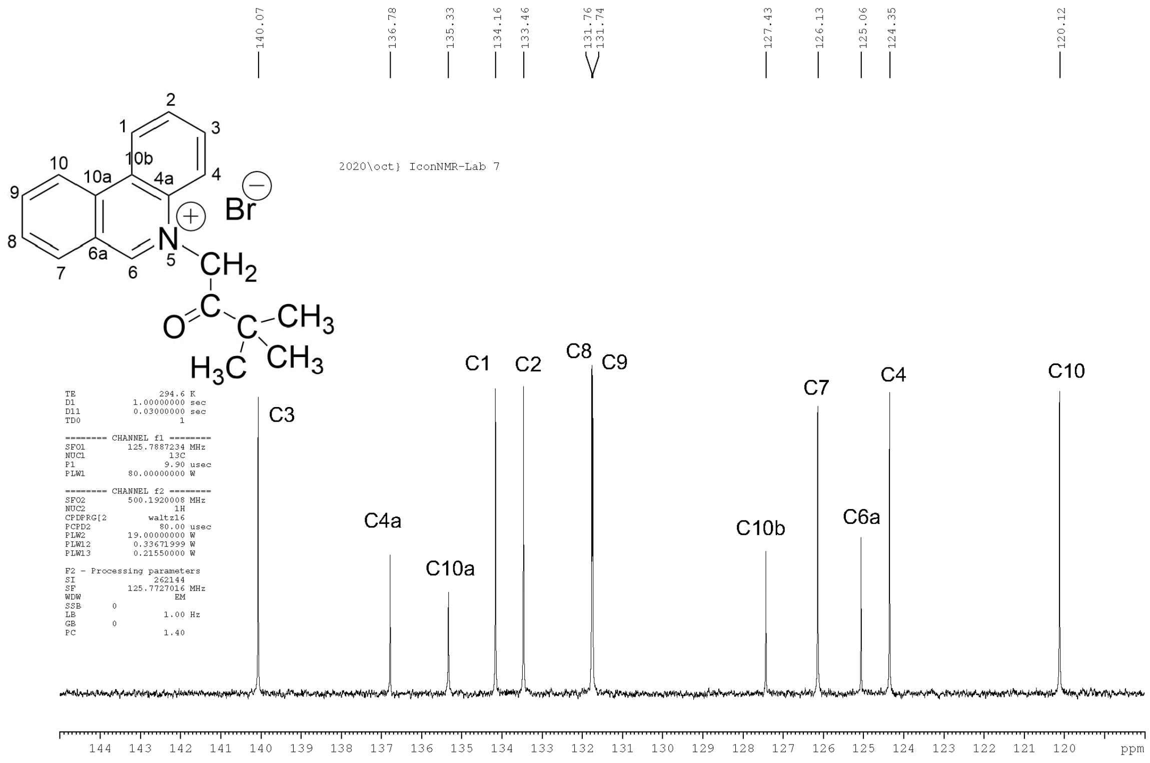

S3c Fig. Detail in the aromatic area of the ^{13}C NMR spectrum of the compound **3c** in MeOD at 125 MHz.

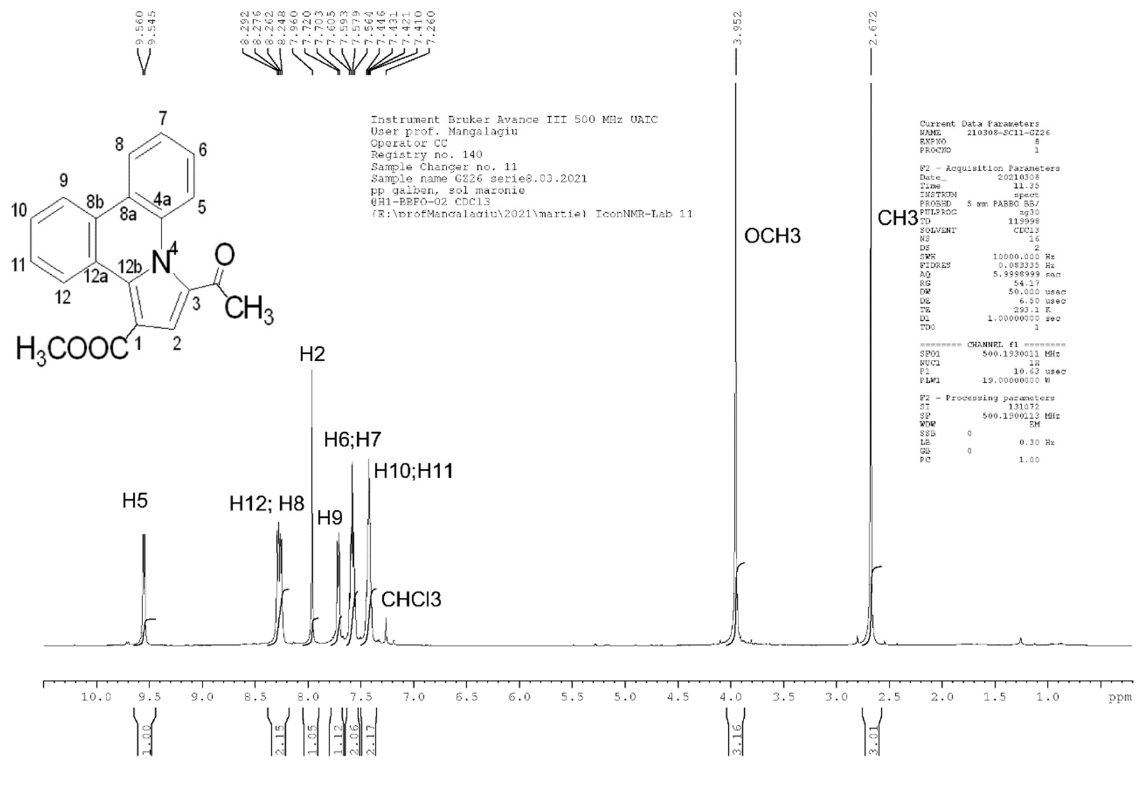

S4a Fig. ^{1}H NMR spectrum of the compound **5a** in $CDCl_3$ at 500 MHz.

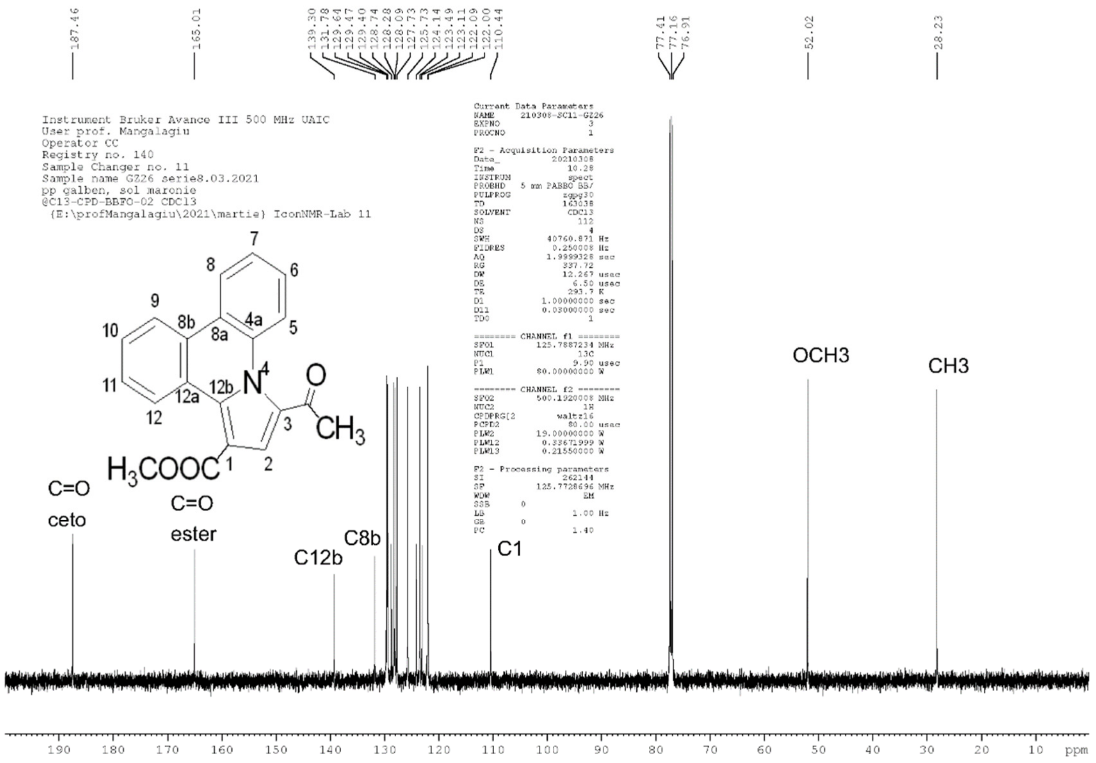

S4b Fig. ^{13}C NMR spectrum of the compound **5a** in $CDCl_3$ at 125 MHz.

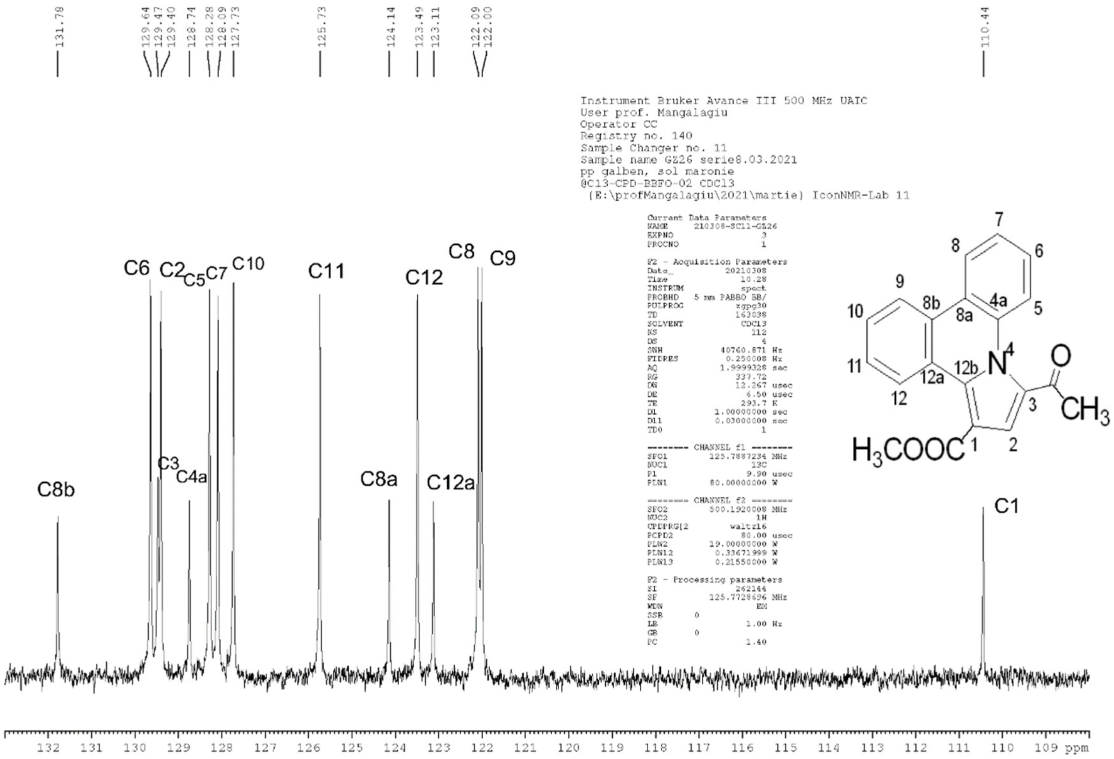

S4c Fig. Detail in the aromatic area of the ^{13}C NMR spectrum of the compound **5a** in $CDCl_3$ at 125 MHz.

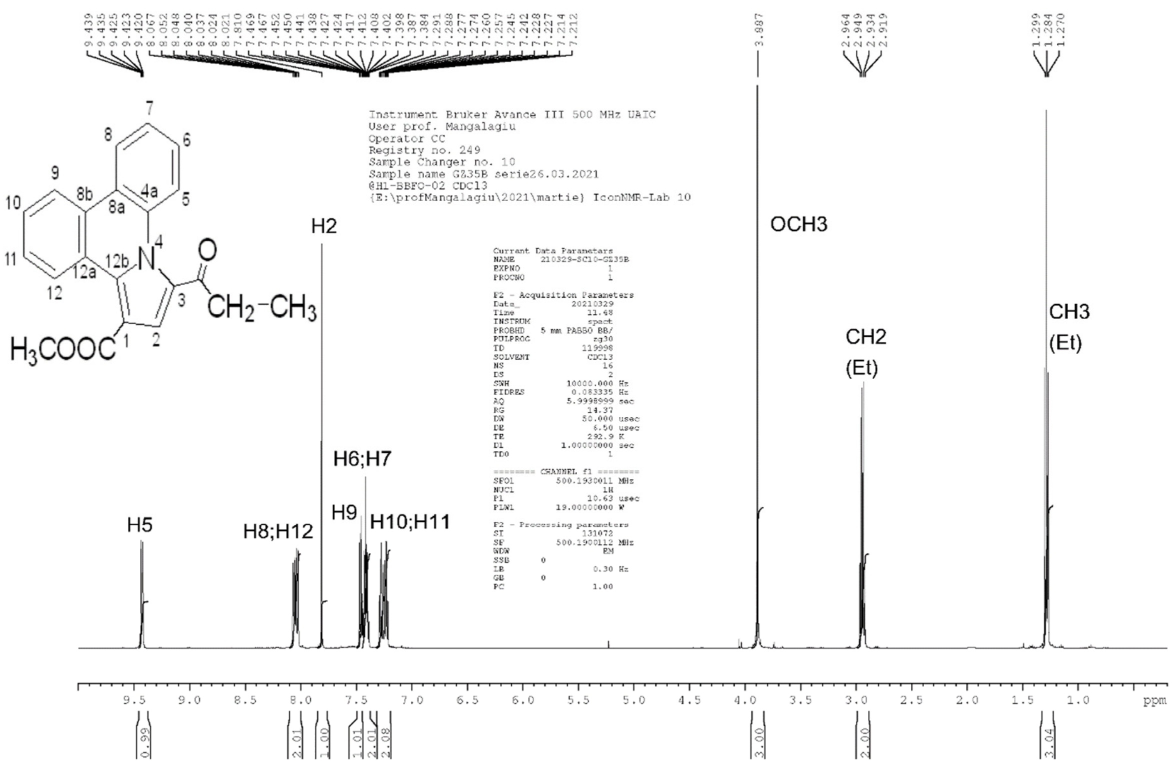

S5a Fig. ^{1}H NMR spectrum of the compound **5b** in $CDCl_3$ at 500 MHz.

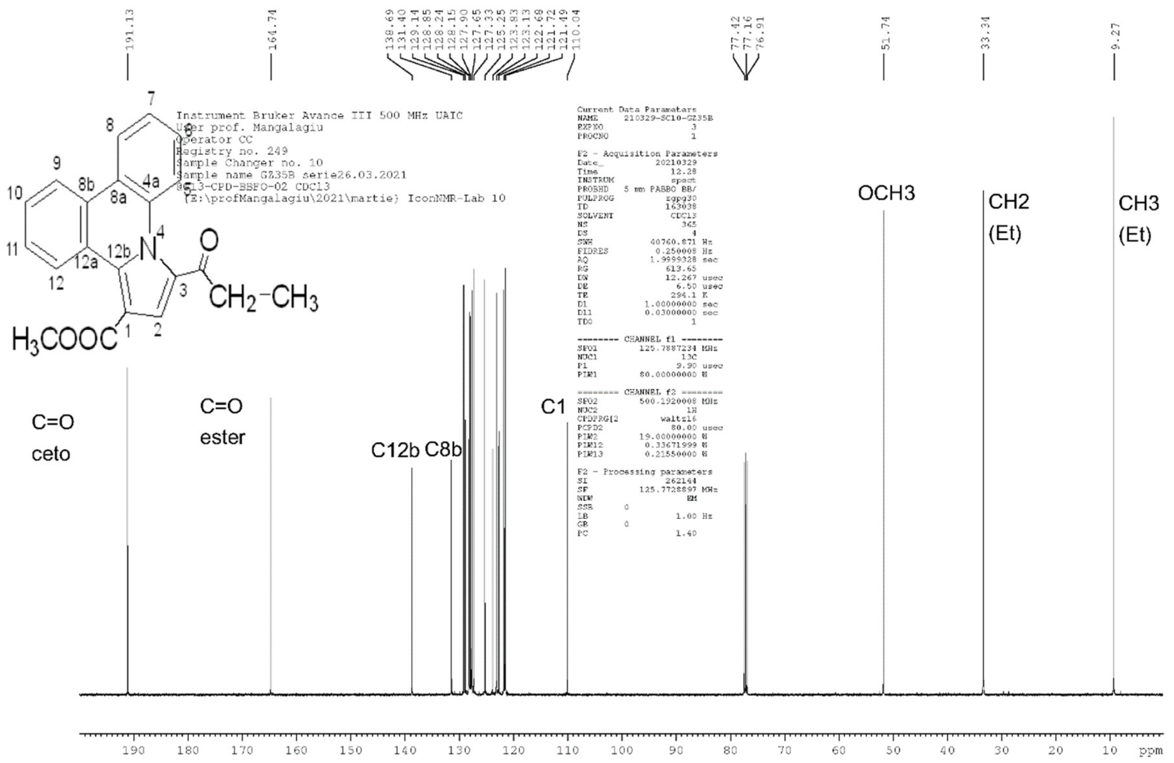

S5b Fig. ^{13}C NMR spectrum of the compound **5b** in $CDCl_3$ at 125 MHz.

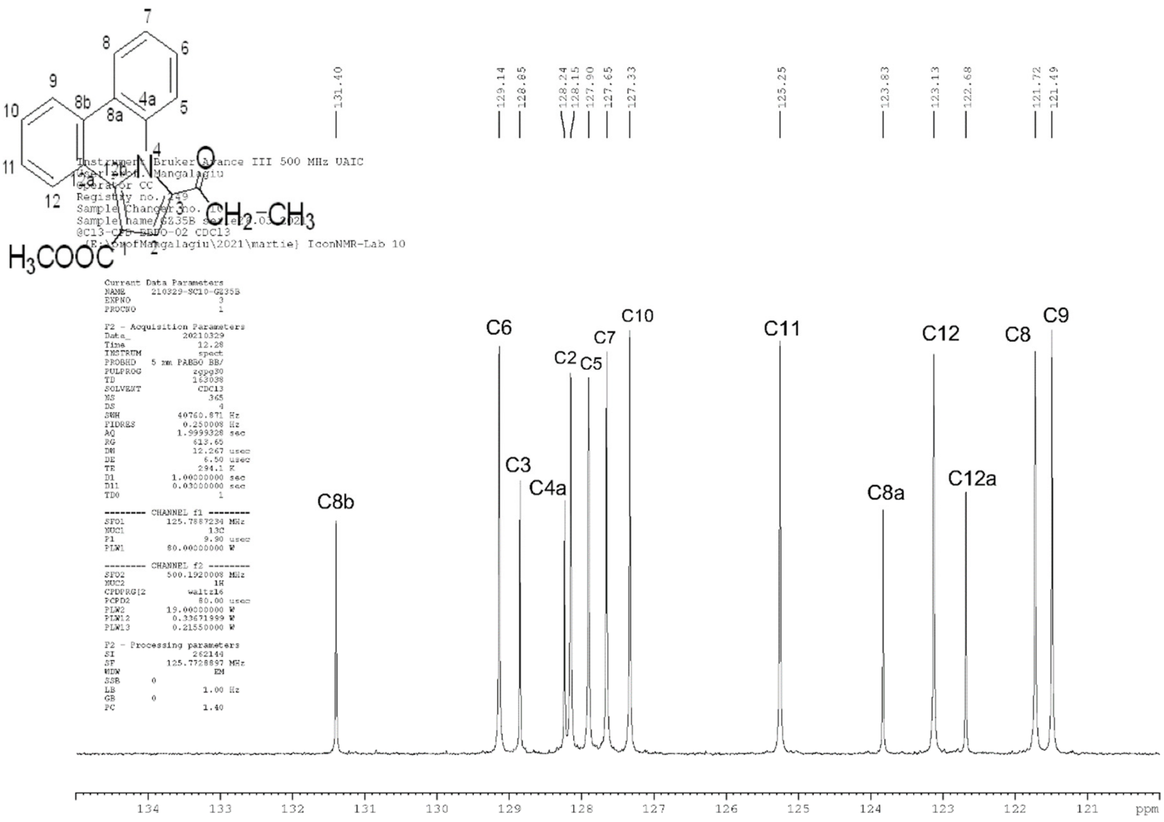

S5c Fig. Detail in the aromatic area of the ^{13}C NMR spectrum of the compound **5b** in $CDCl_3$ at 125 MHz.

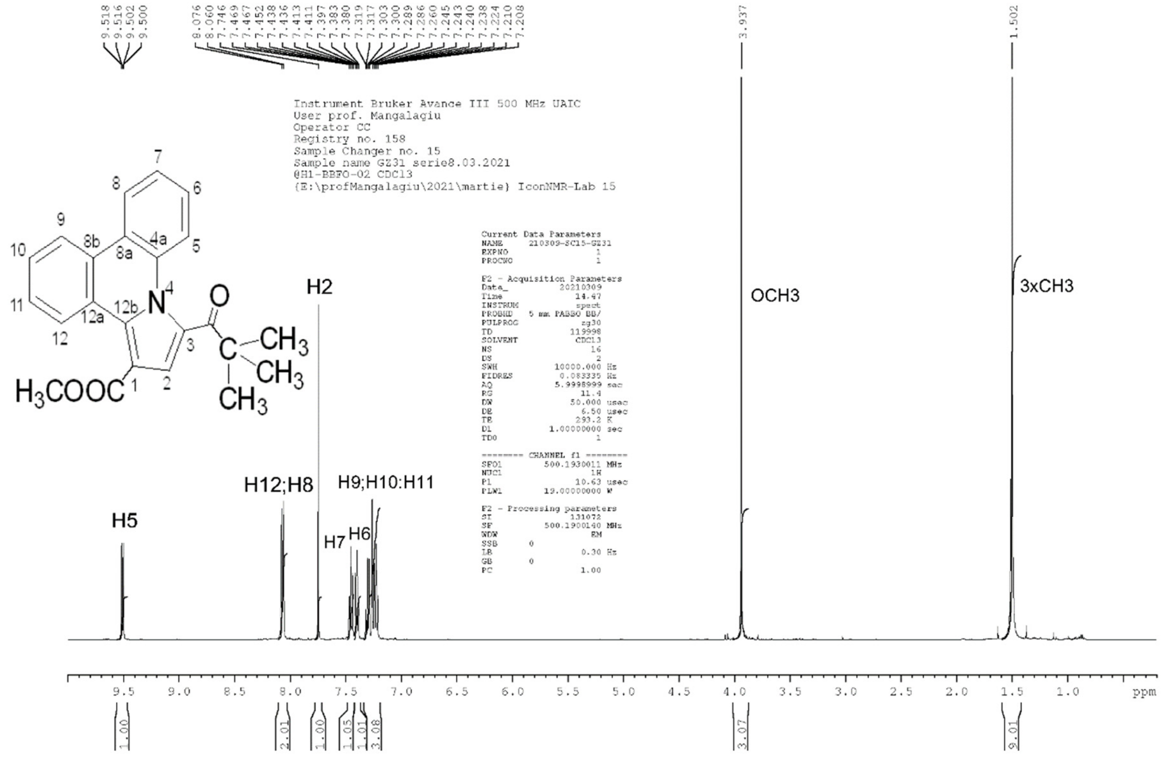

S6a Fig. ^{1}H NMR spectrum of the compound **5c** in $CDCl_3$ at 500 MHz.

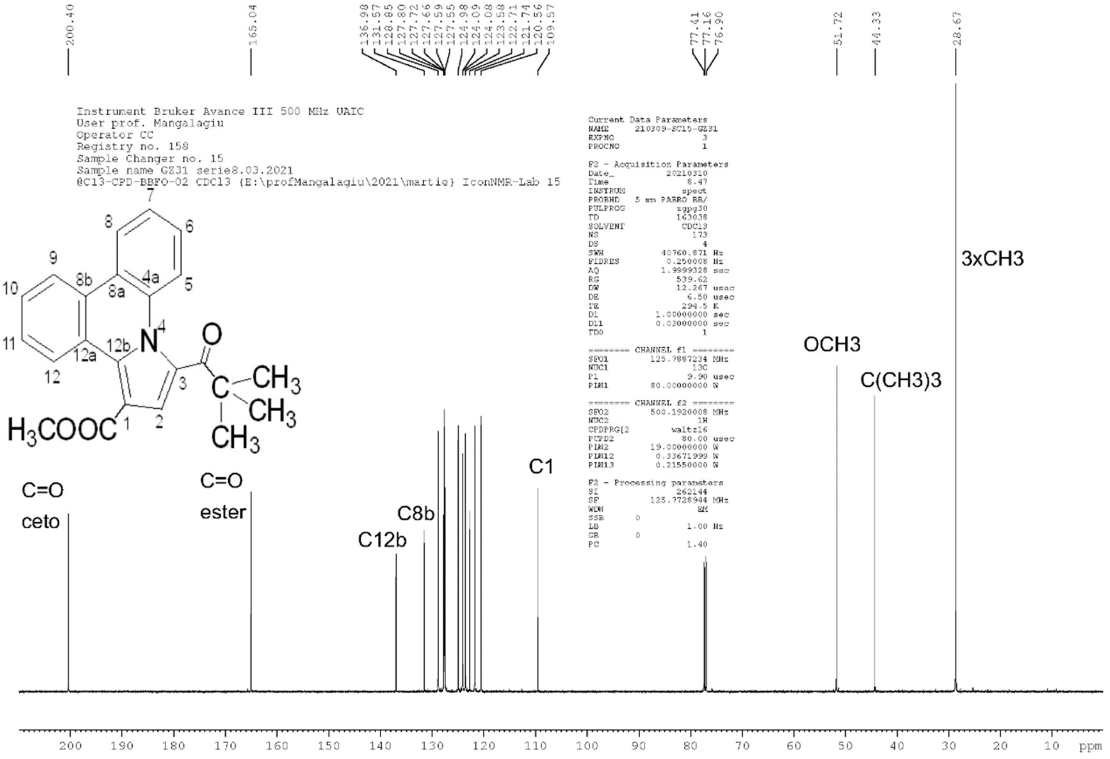

S6b Fig. ^{13}C NMR spectrum of the compound **5c** in $CDCl_3$ at 125 MHz.

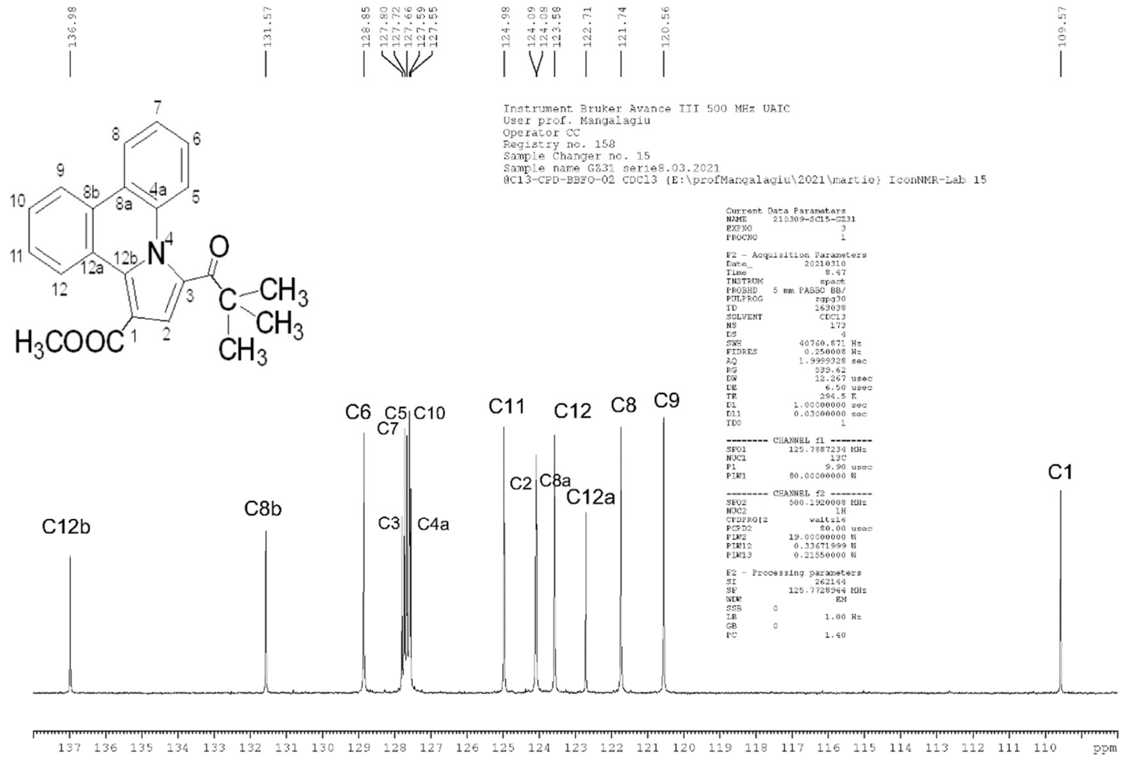

S6c Fig. Detail in the aromatic area of the ^{13}C NMR spectrum of the compound **5c** in $CDCl_3$ at 125 MHz.

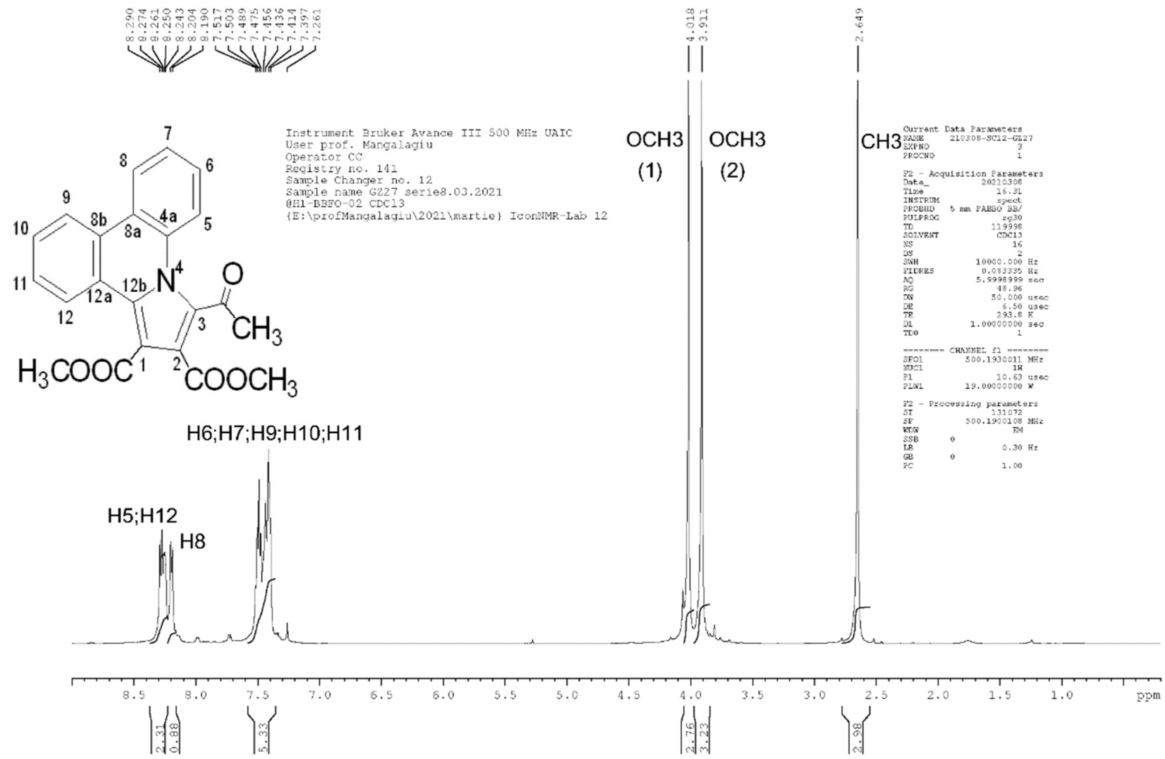

S7a Fig. ^{1}H NMR spectrum of the compound **6a** in $CDCl_3$ at 500 MHz.

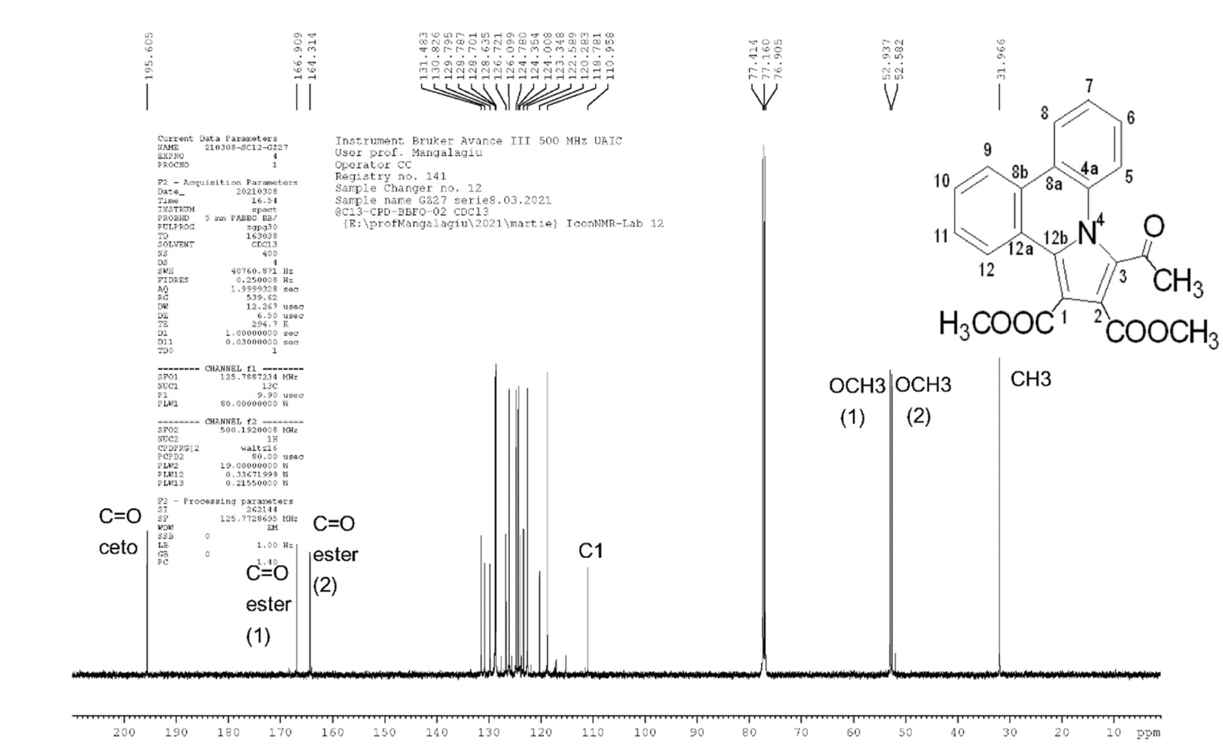

S7b Fig. ^{13}C NMR spectrum of the compound **6a** in $CDCl_3$ at 125 MHz.

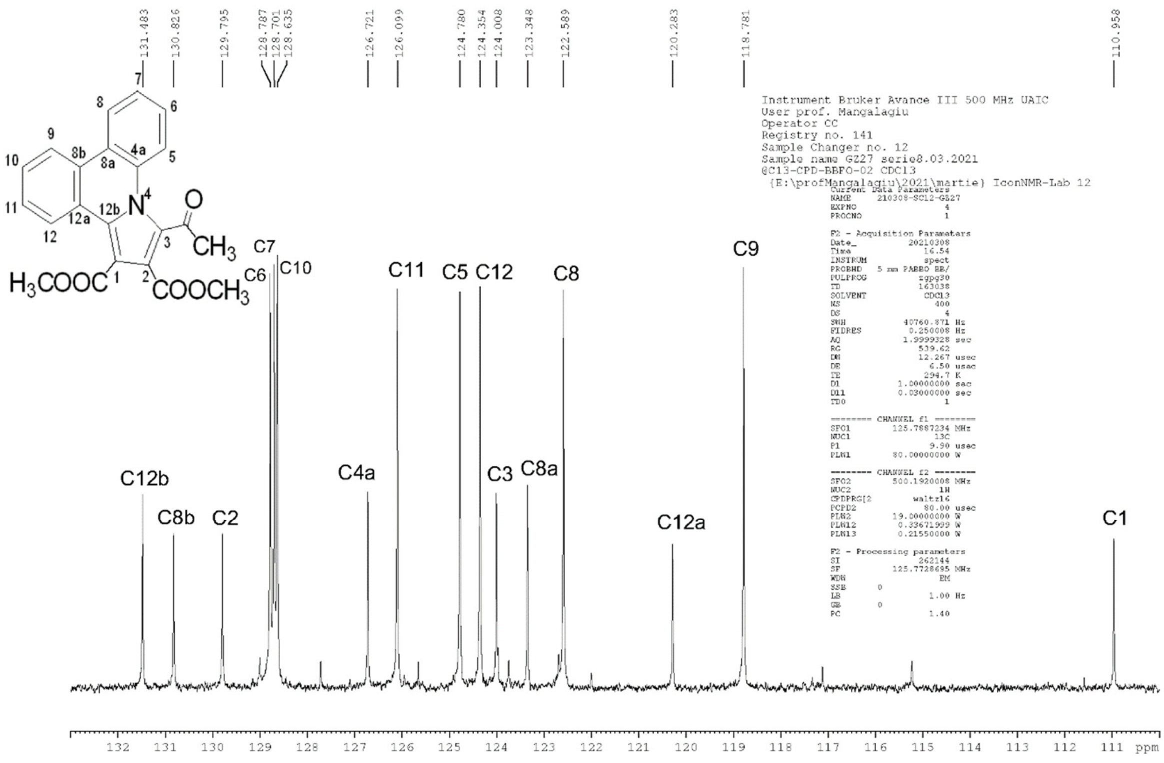

S7c Fig. Detail in the aromatic area of the ^{13}C NMR spectrum of the compound **6a** in $CDCl_3$ at 125 MHz.

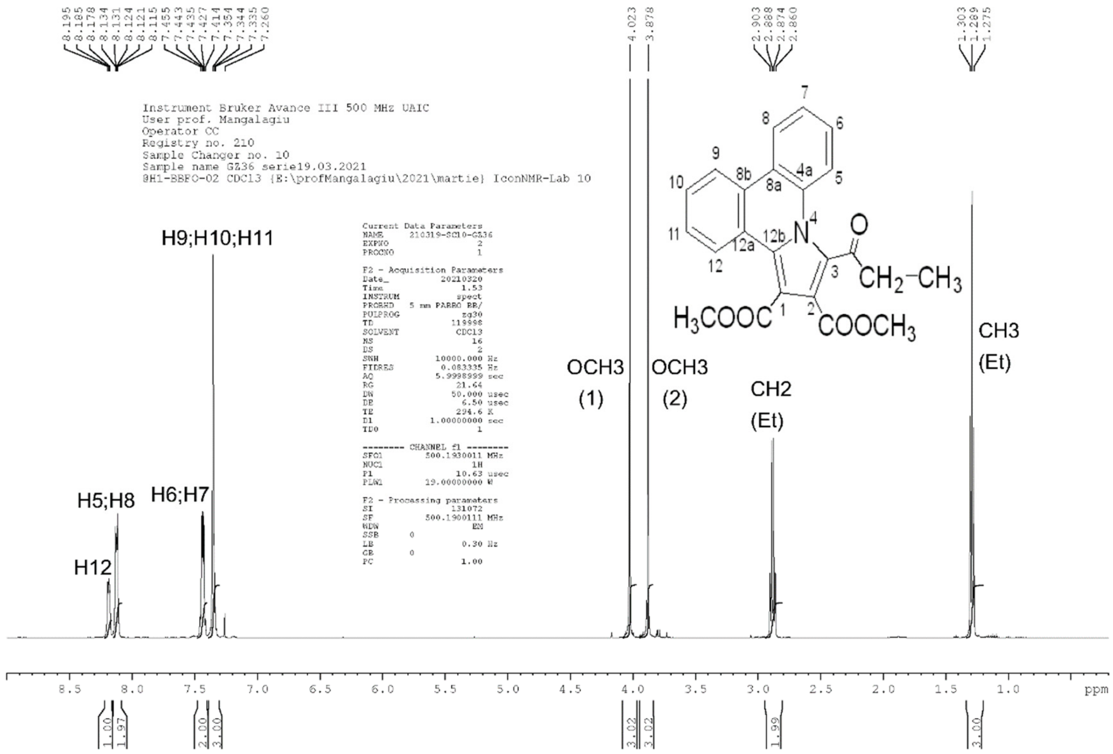

S8a Fig. ^{1}H NMR spectrum of the compound **6b** in $CDCl_3$ at 500 MHz.

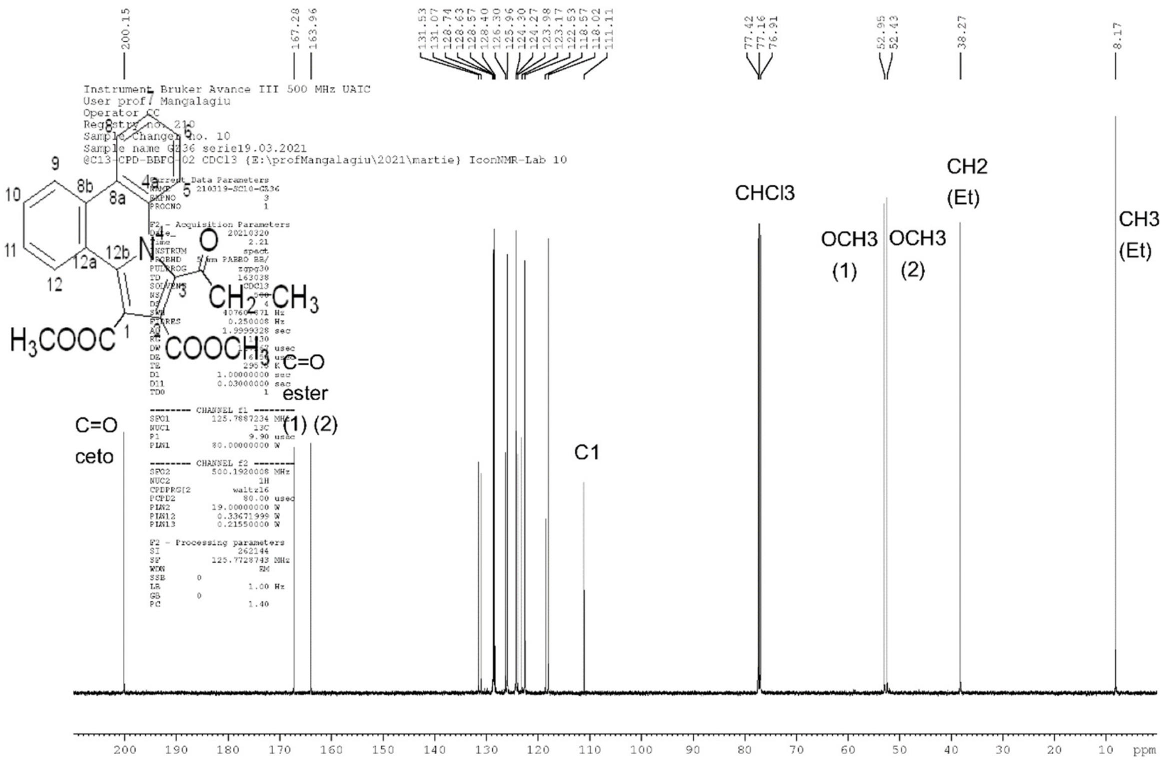

S8b Fig. ^{13}C NMR spectrum of the compound **6b** in $CDCl_3$ at 125 MHz.

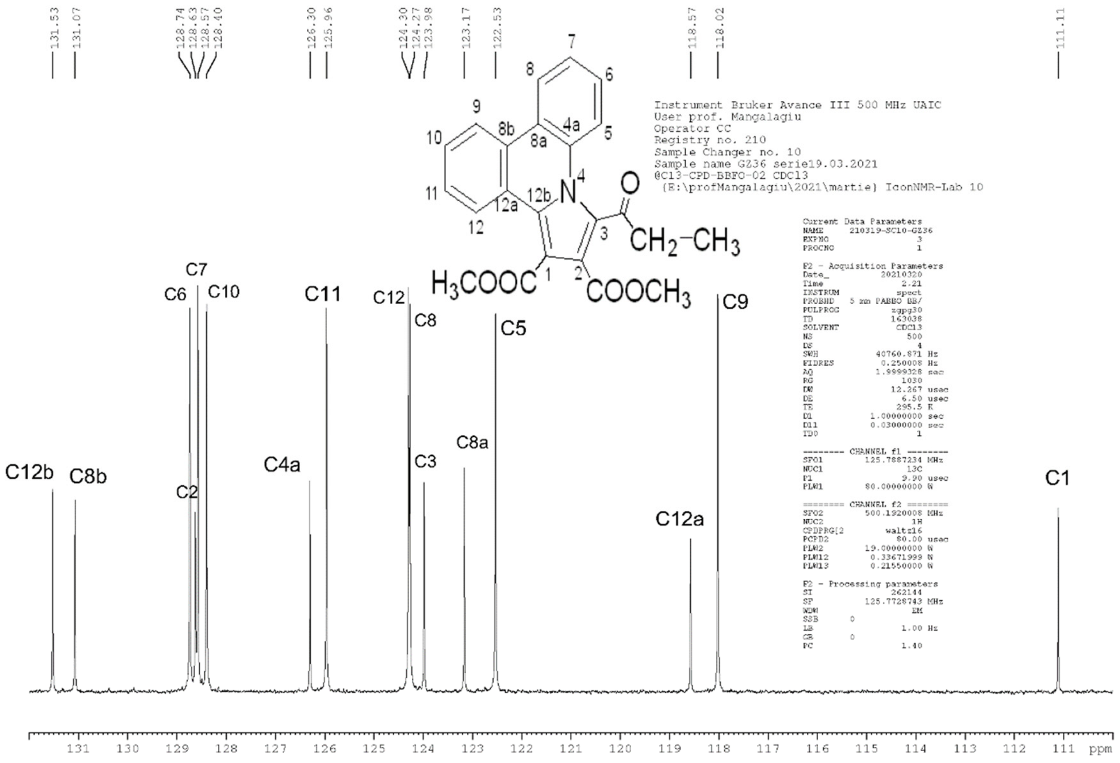

S8c Fig. Detail in the aromatic area of the ^{13}C NMR spectrum of the compound **6b** in $CDCl_3$ at 125 MHz.

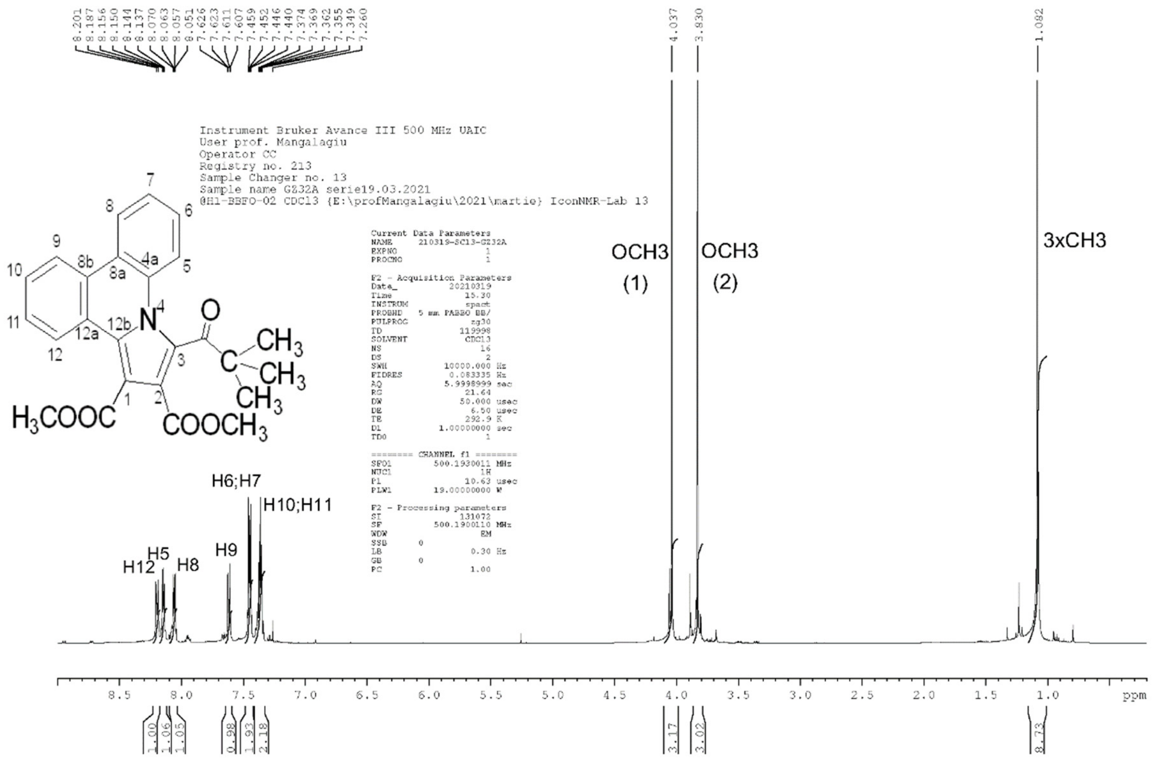

S9a Fig. ^{1}H NMR spectrum of the compound **6c** in $CDCl_3$ at 500 MHz.

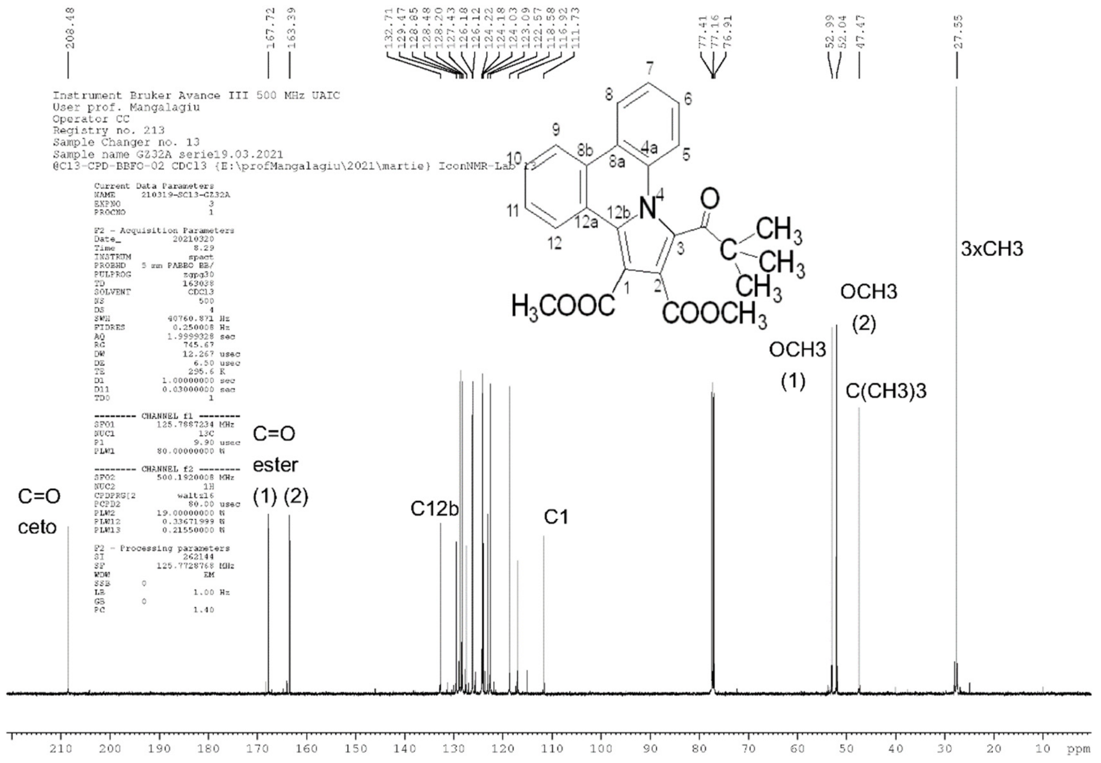

S9b Fig. ^{13}C NMR spectrum of the compound **6c** in $CDCl_3$ at 125 MHz.

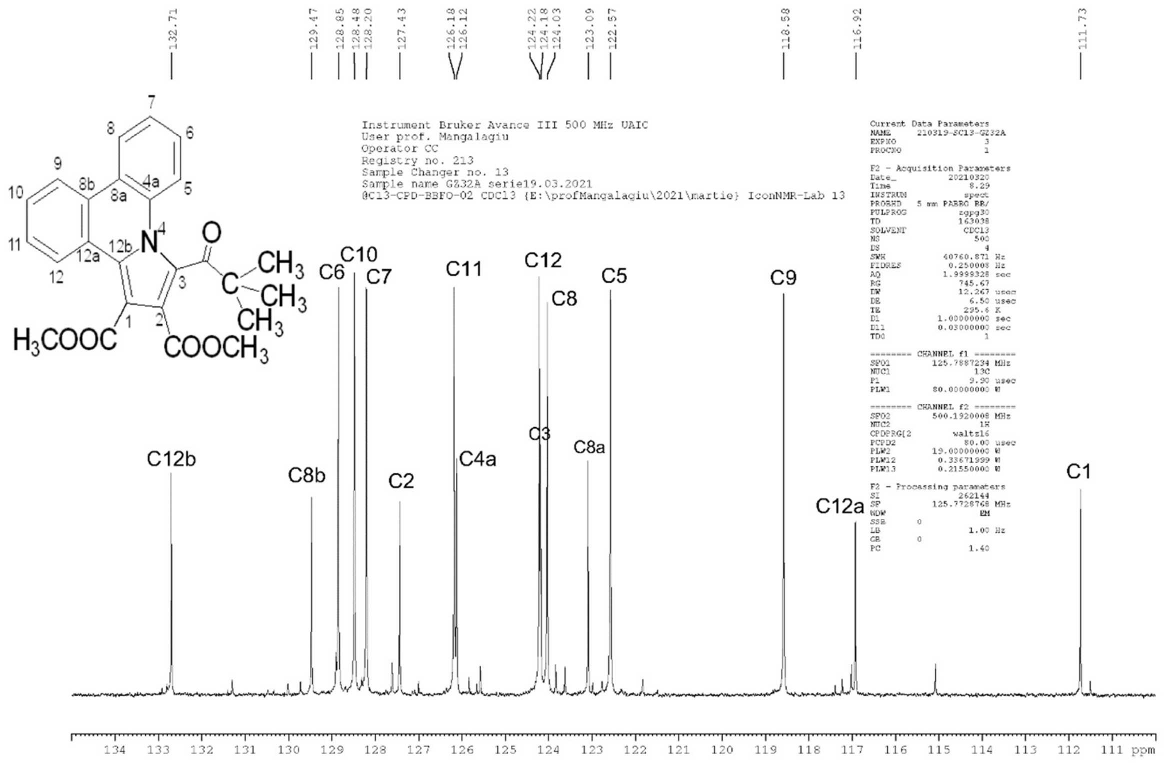

S9c Fig. Detail in the aromatic area of the ^{13}C NMR spectrum of the compound **6c** in $CDCl_3$ at 125 MHz.

3. IR Spectra of the Obtained Compounds

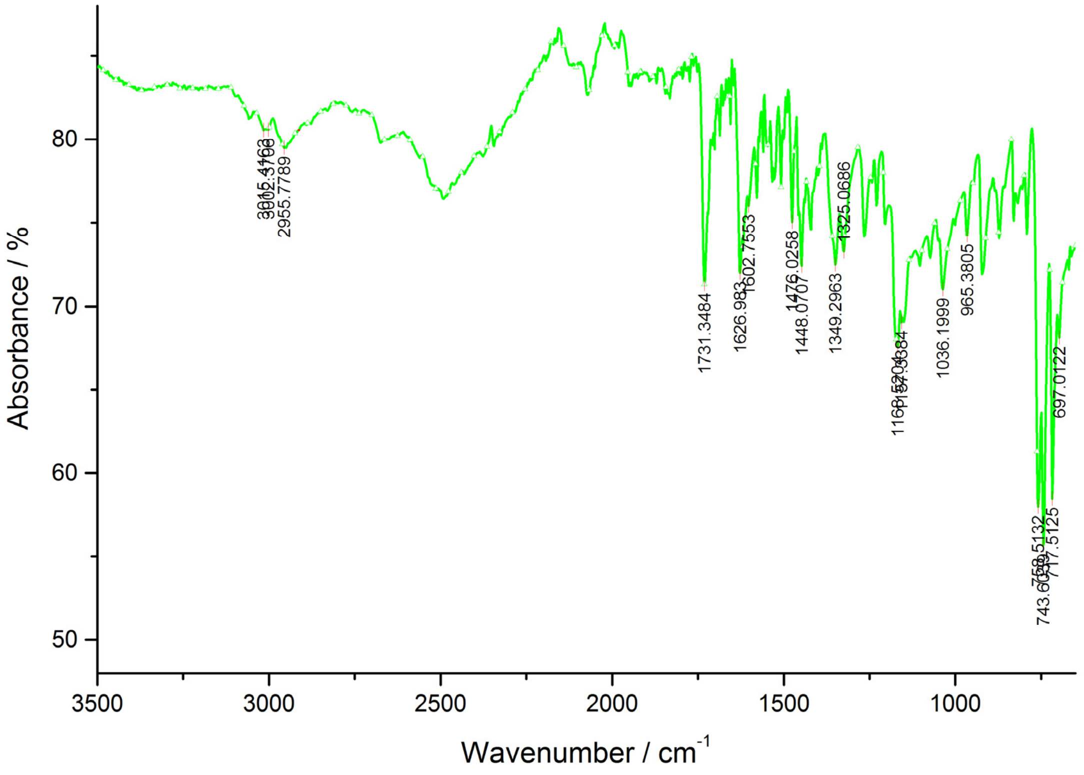

S10 Fig. IR spectrum of the compound **3a** in powder on the diamond crystal ATR mode.

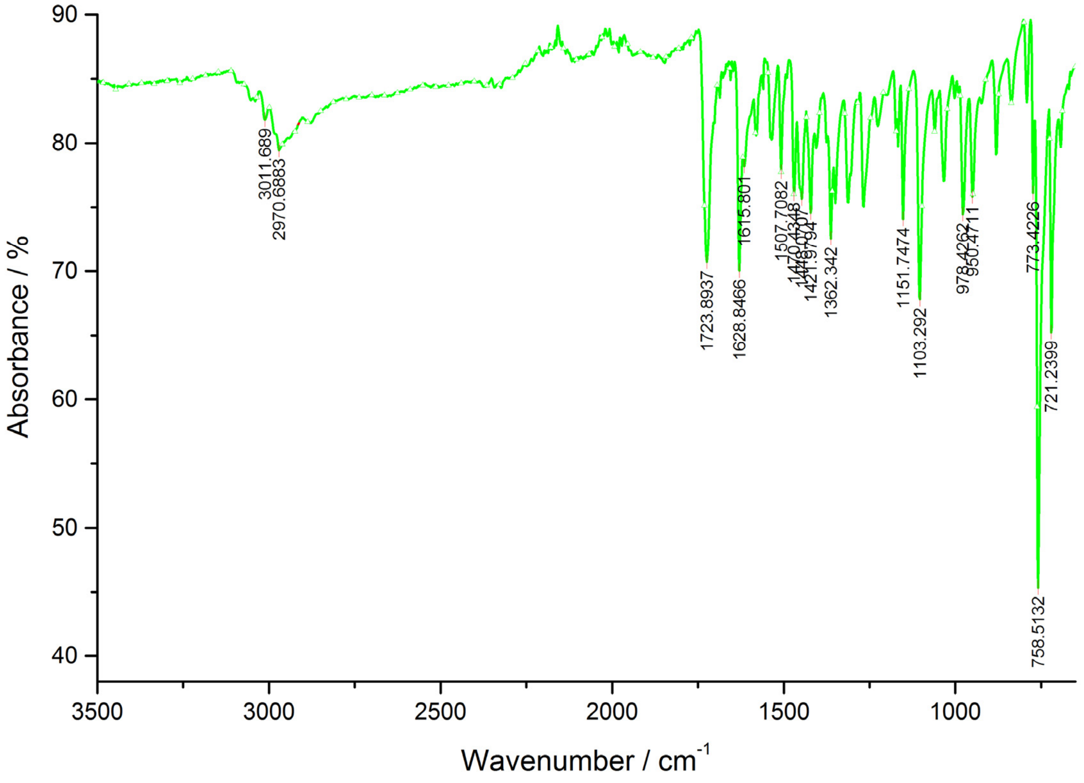

S11 Fig. IR spectrum of the compound **3b** in powder on the diamond crystal ATR mode.

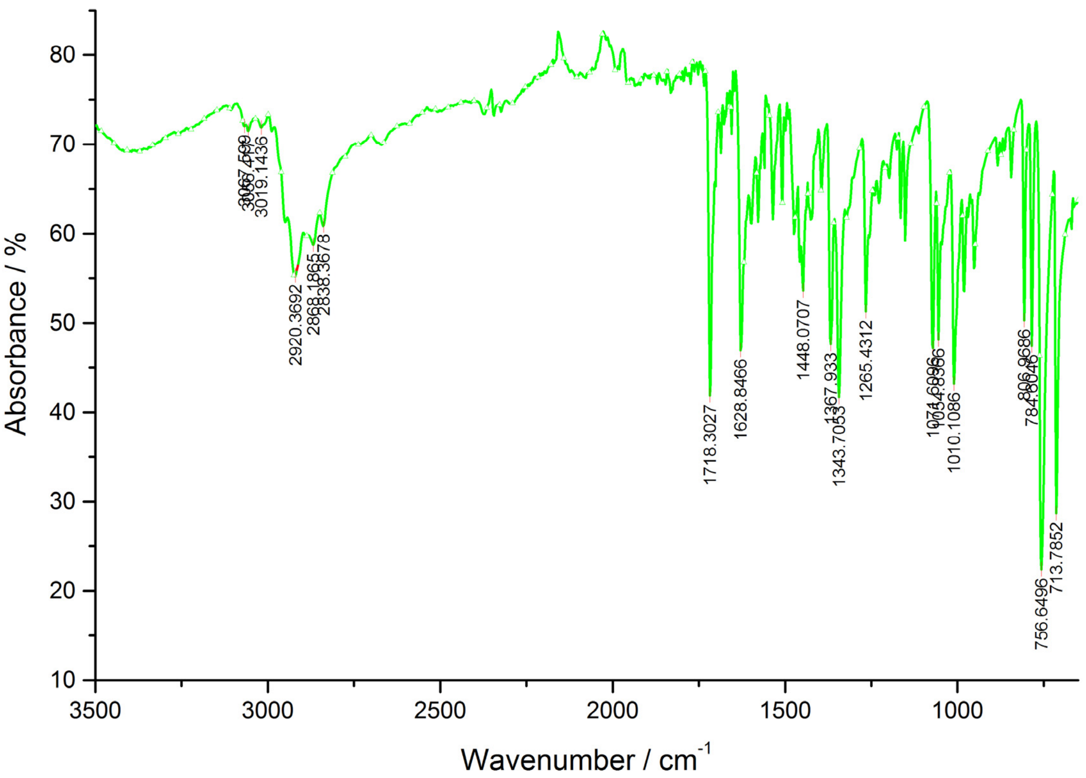

S12 Fig. IR spectrum of the compound **3c** in powder on the diamond crystal ATR mode.

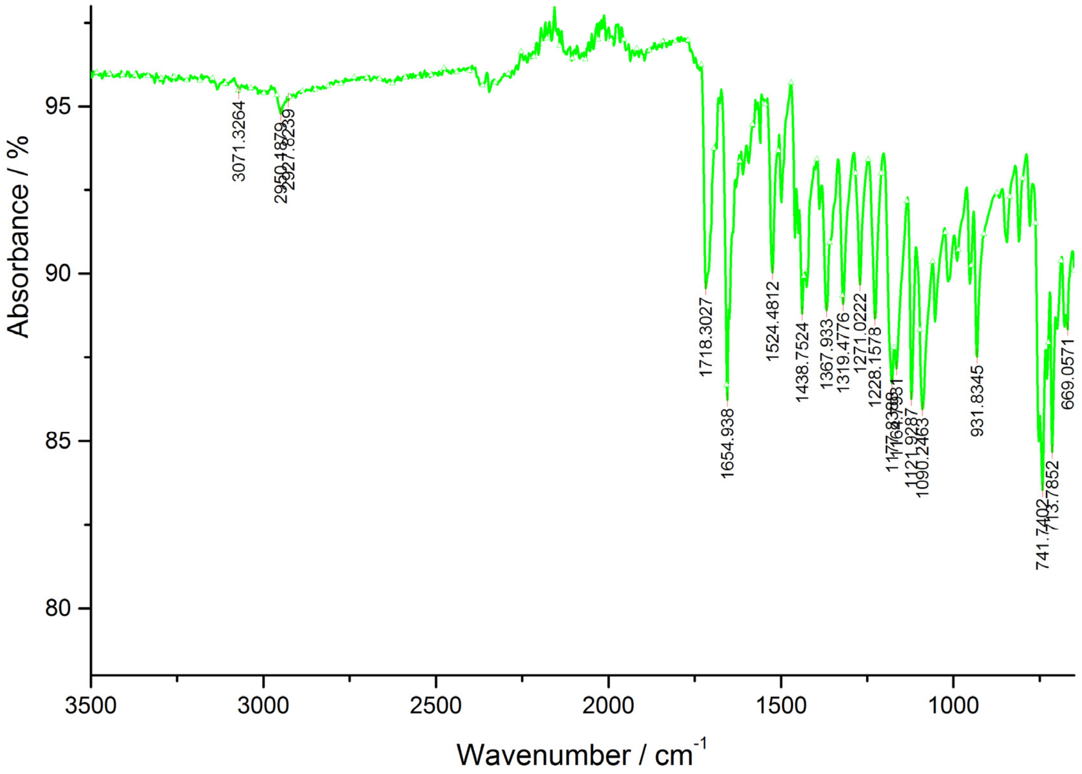

S13 Fig. IR spectrum of the compound **5a** in powder on the diamond crystal ATR mode.

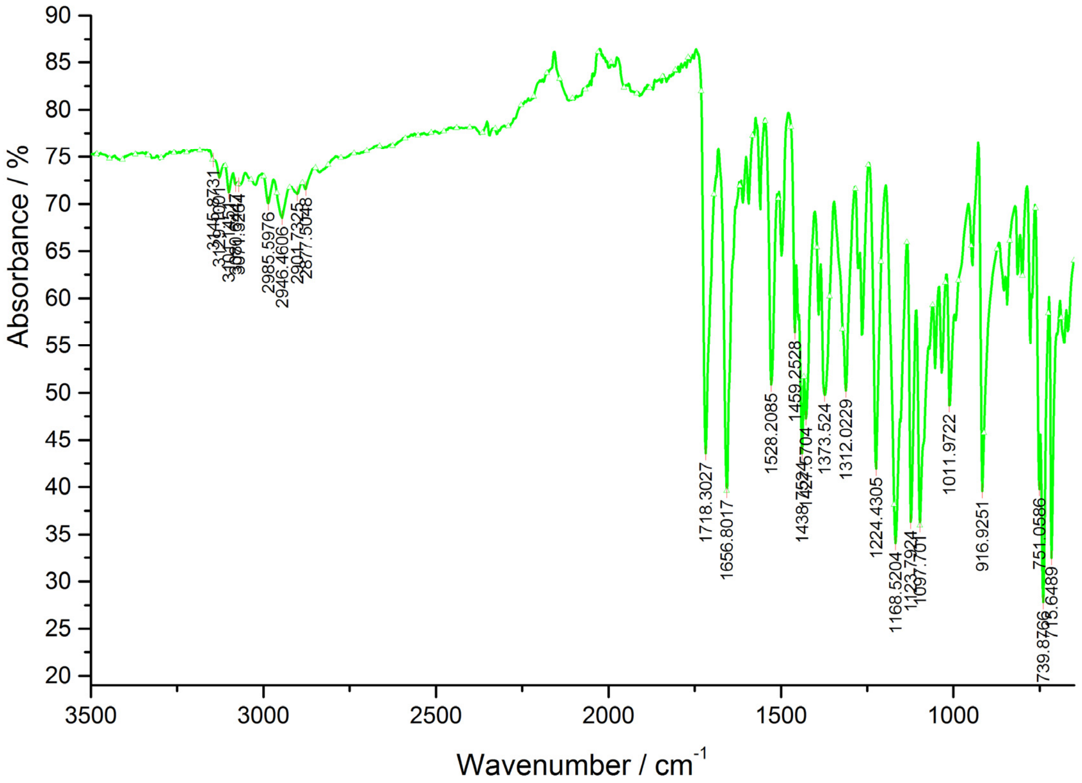

S14 Fig. IR spectrum of the compound **5b** in powder on the diamond crystal ATR mode.

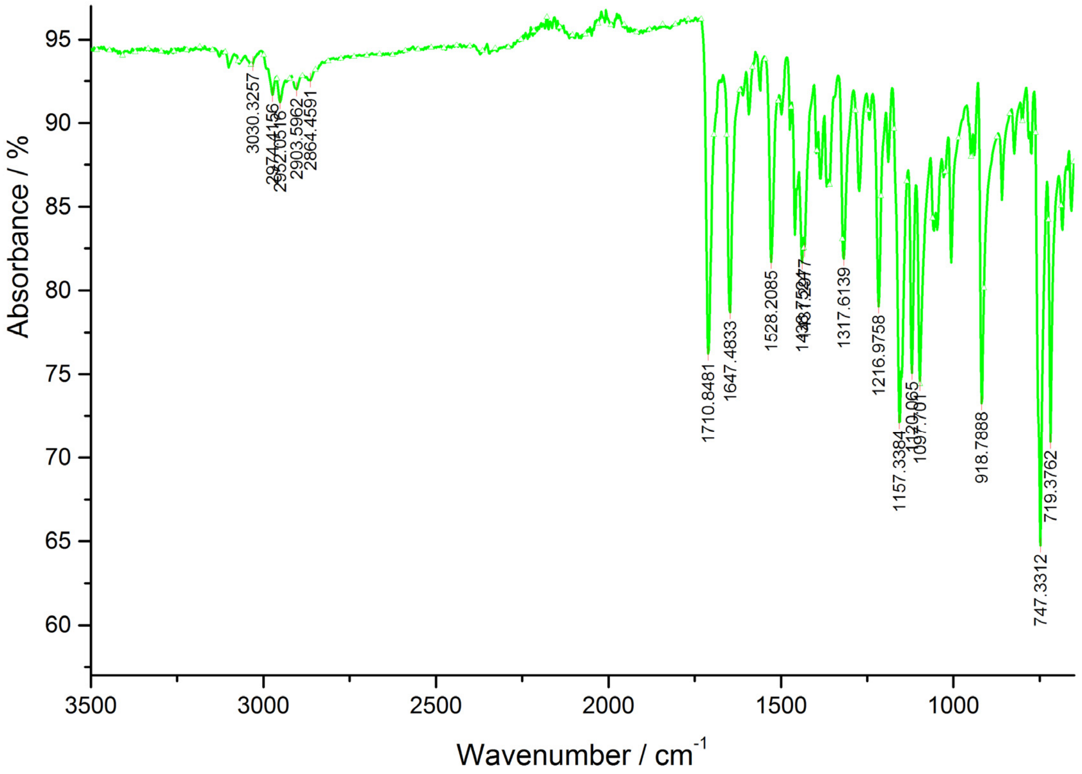

S15 Fig. IR spectrum of the compound **5c** in powder on the diamond crystal ATR mode.

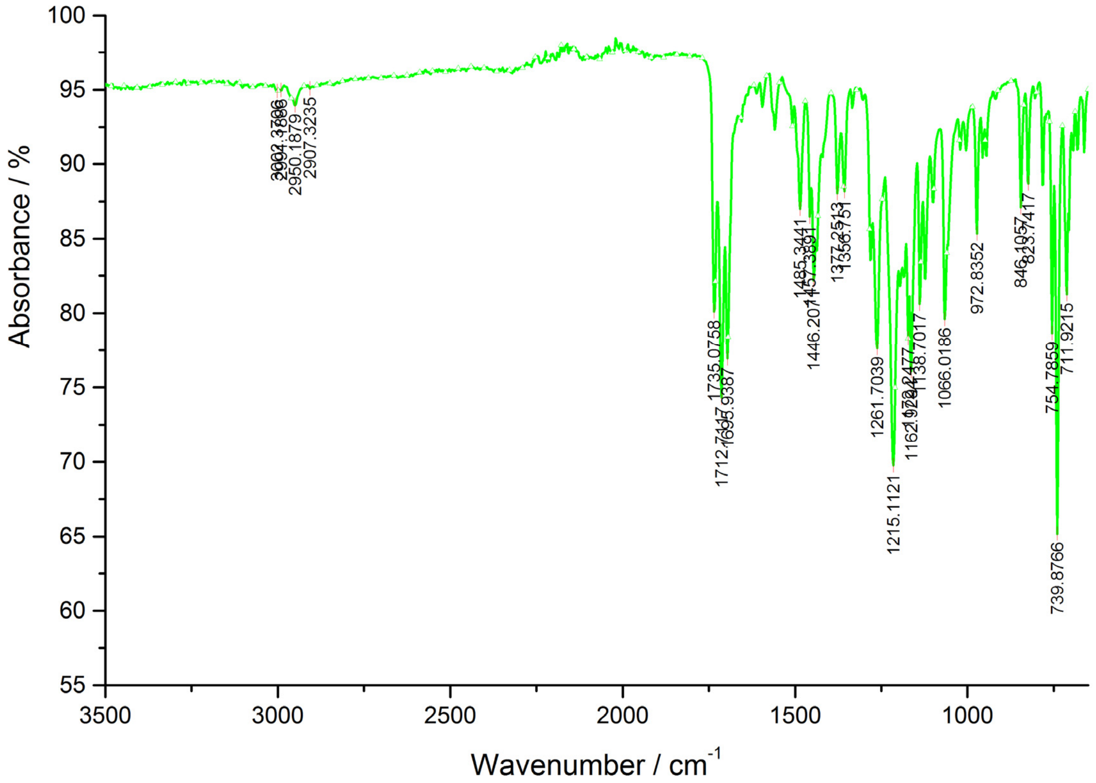

S16 Fig. IR spectrum of the compound **6a** in powder on the diamond crystal ATR mode**.**

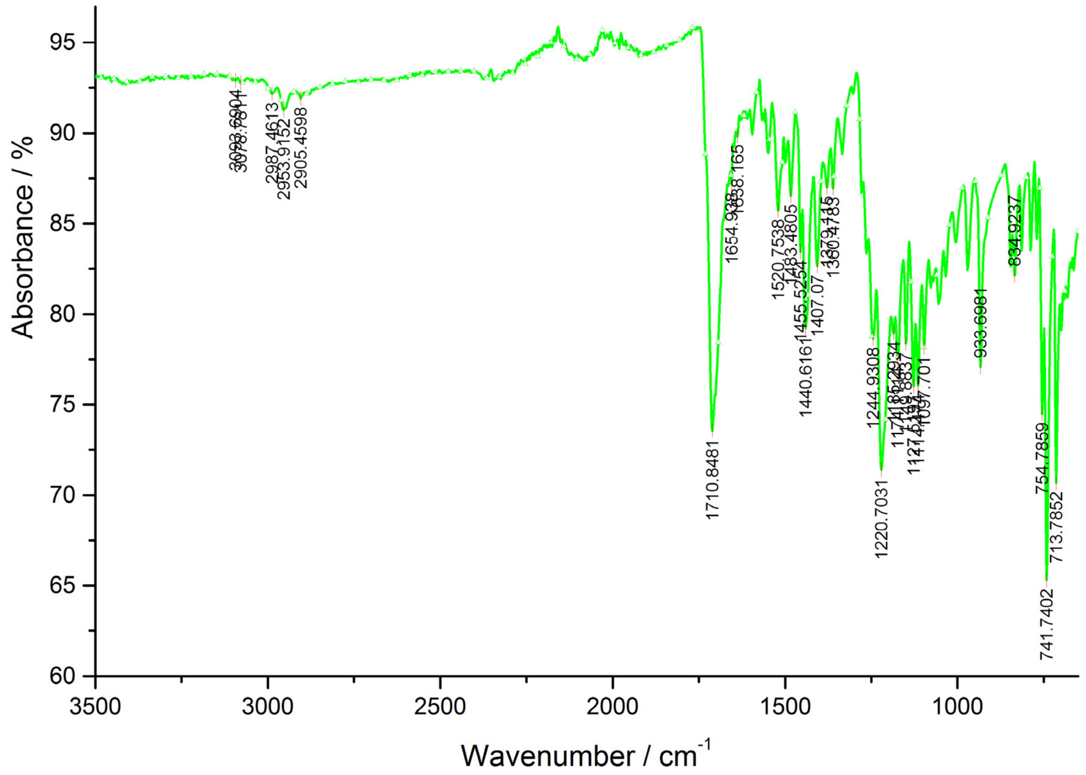

S17 Fig. IR spectrum of the compound **6b** in powder on the diamond crystal ATR mode**.**

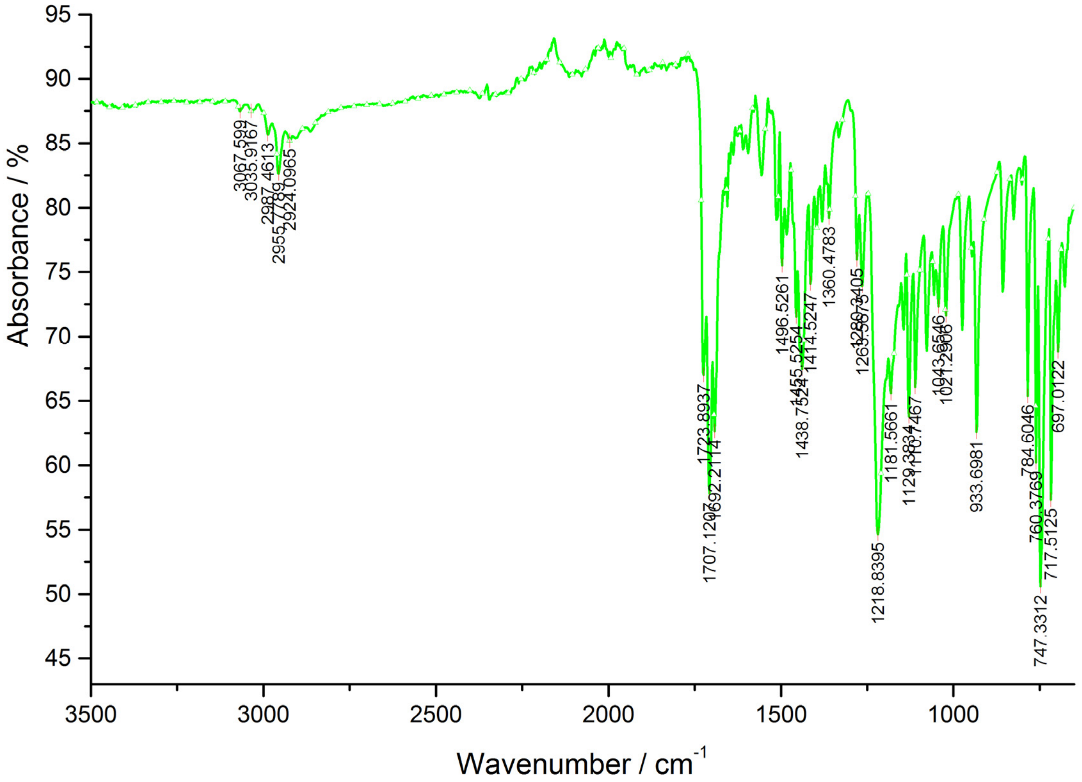

S18 Fig. IR spectrum of the compound **6c** in powder on the diamond crystal ATR mode.

4. HR-MS Spectra of the Obtained Compounds

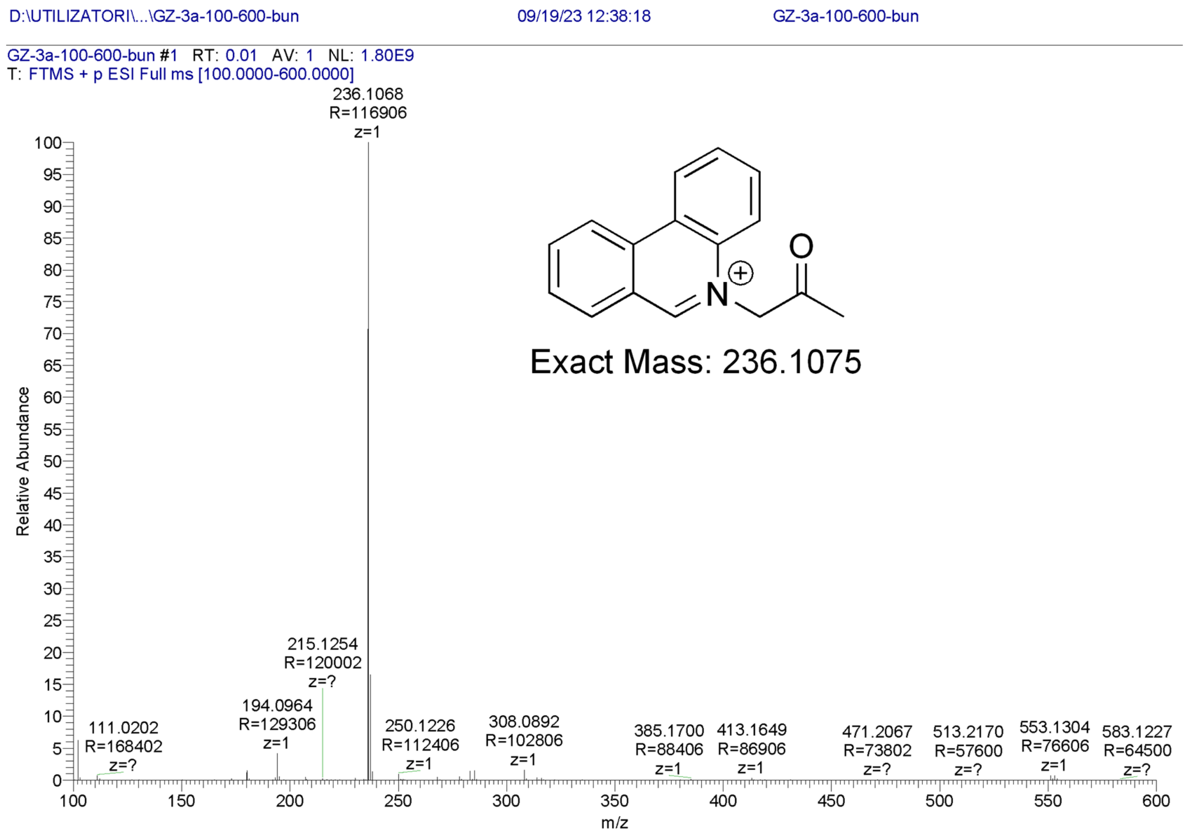

S19 Fig. HR-MS spectra of the compound **3a** in positive mode.

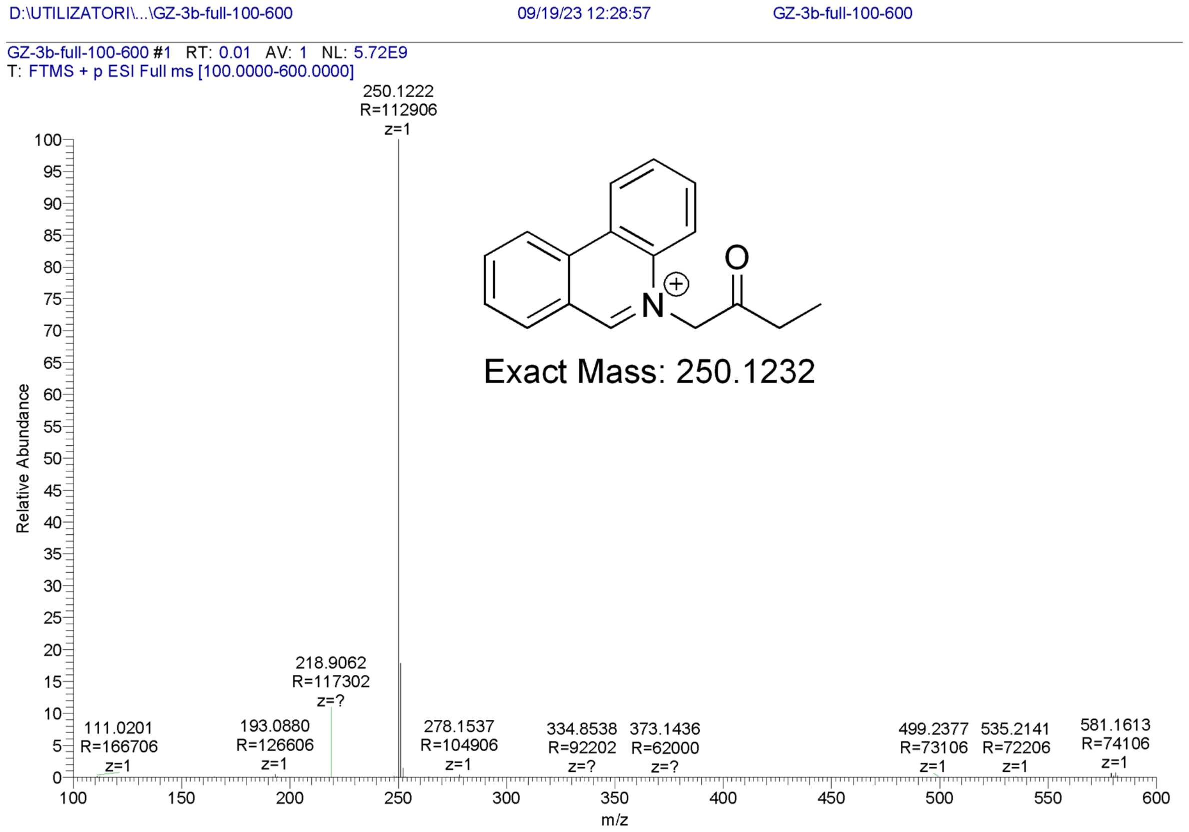

S20 Fig. HR-MS spectra of the compound **3b** in positive mode.

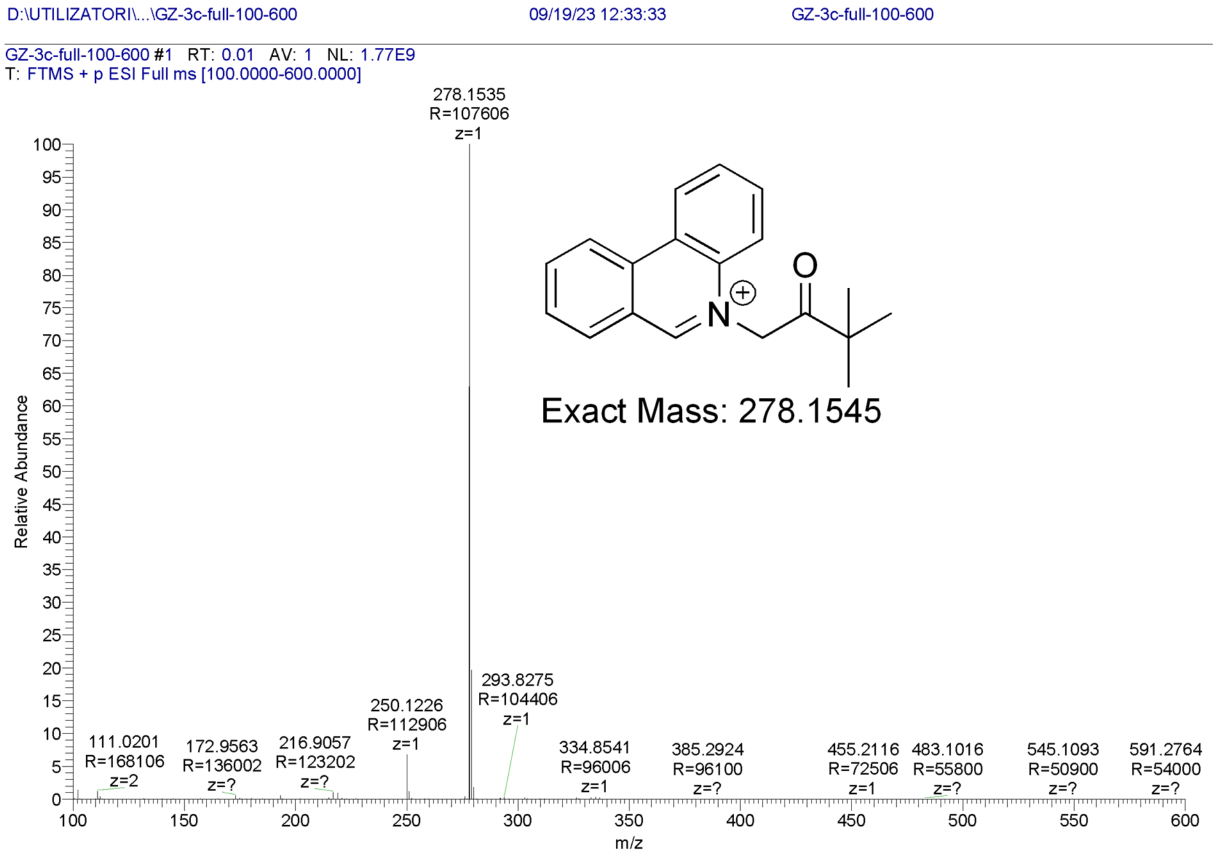

S21 Fig. HR-MS spectra of the compound **3c** in positive mode.

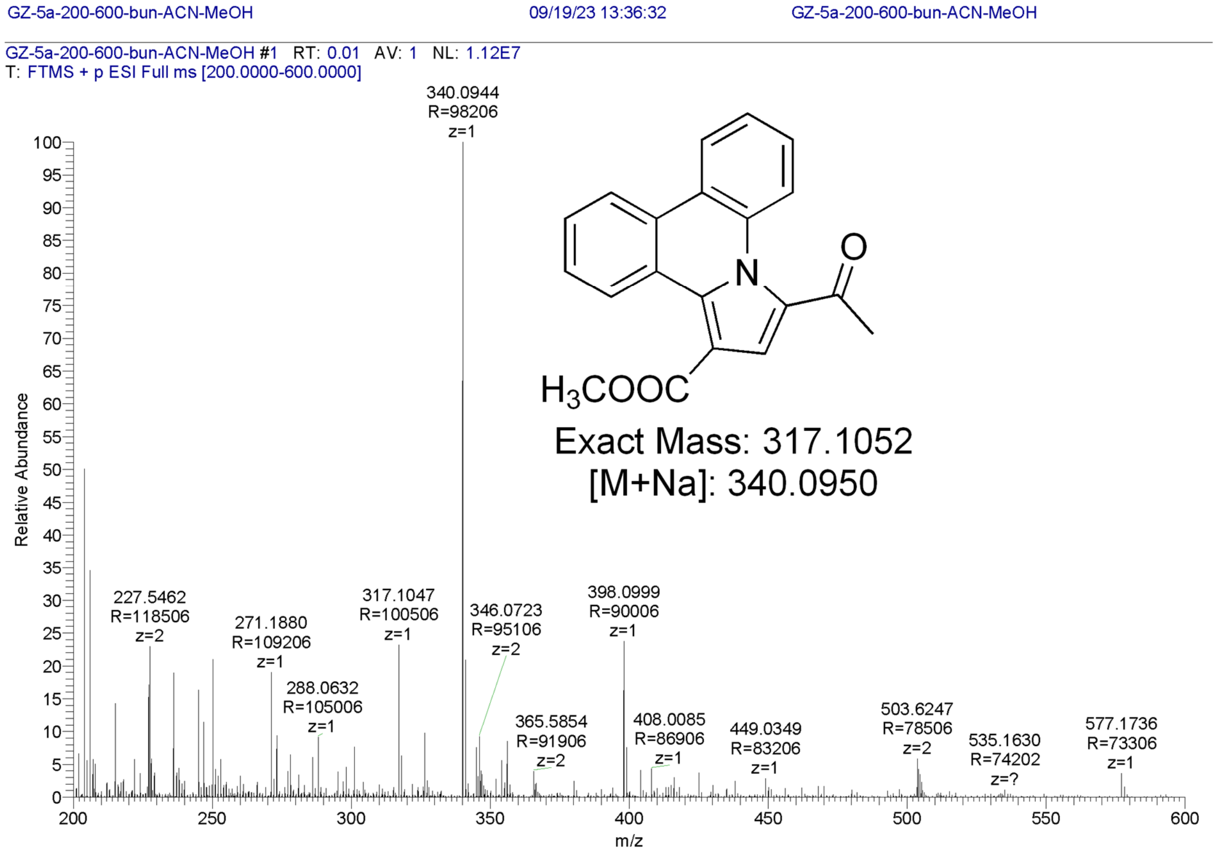

S22 Fig. HR-MS spectra of the compound **5a** in positive mode.

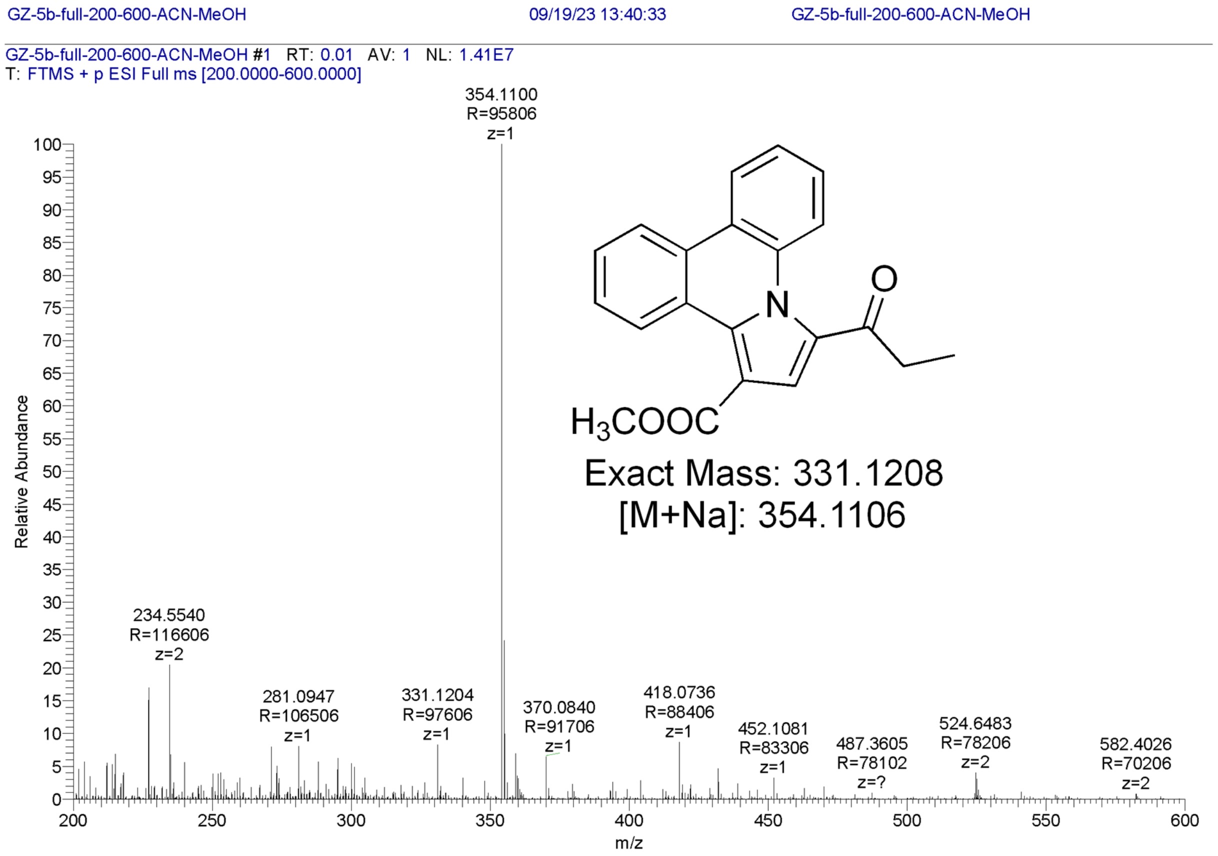

S23 Fig. HR-MS spectra of the compound **5b** in positive mode.

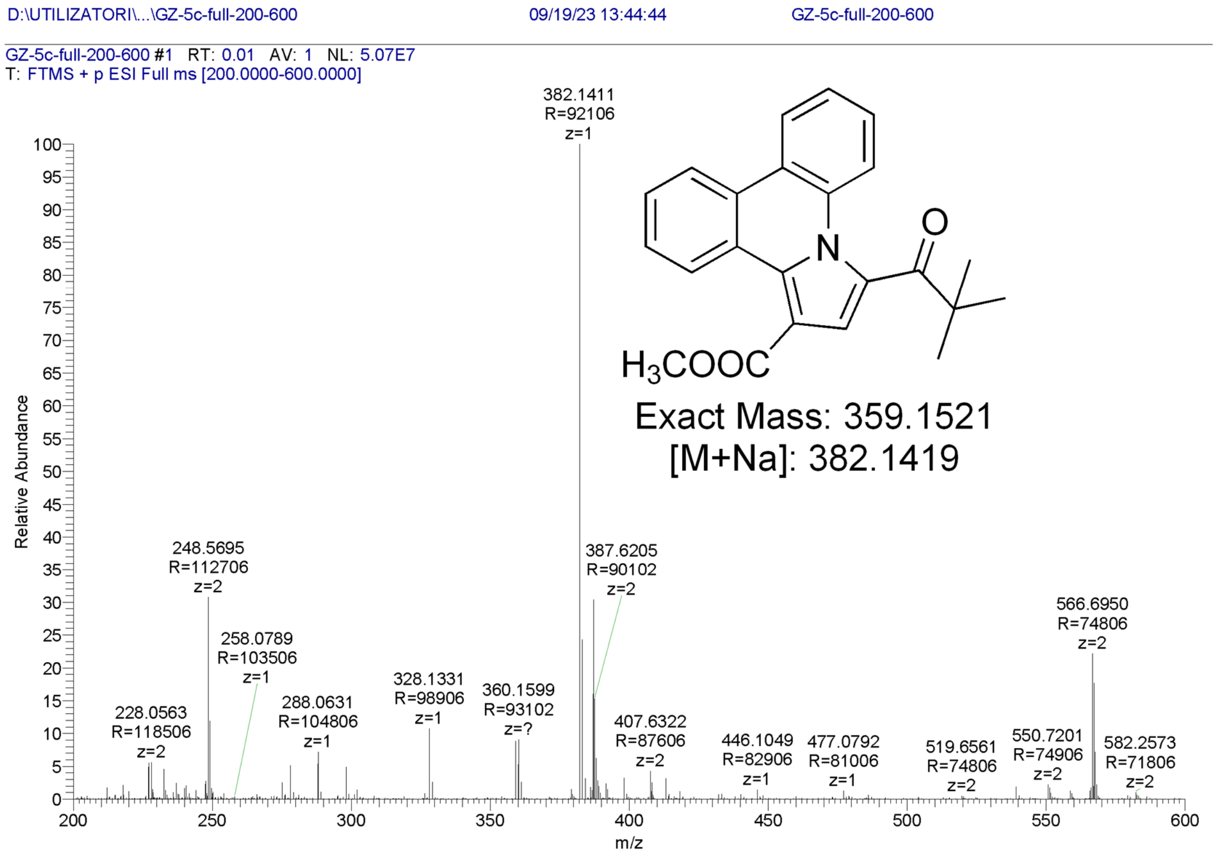

S24 Fig. HR-MS spectra of the compound **5c** in positive mode.

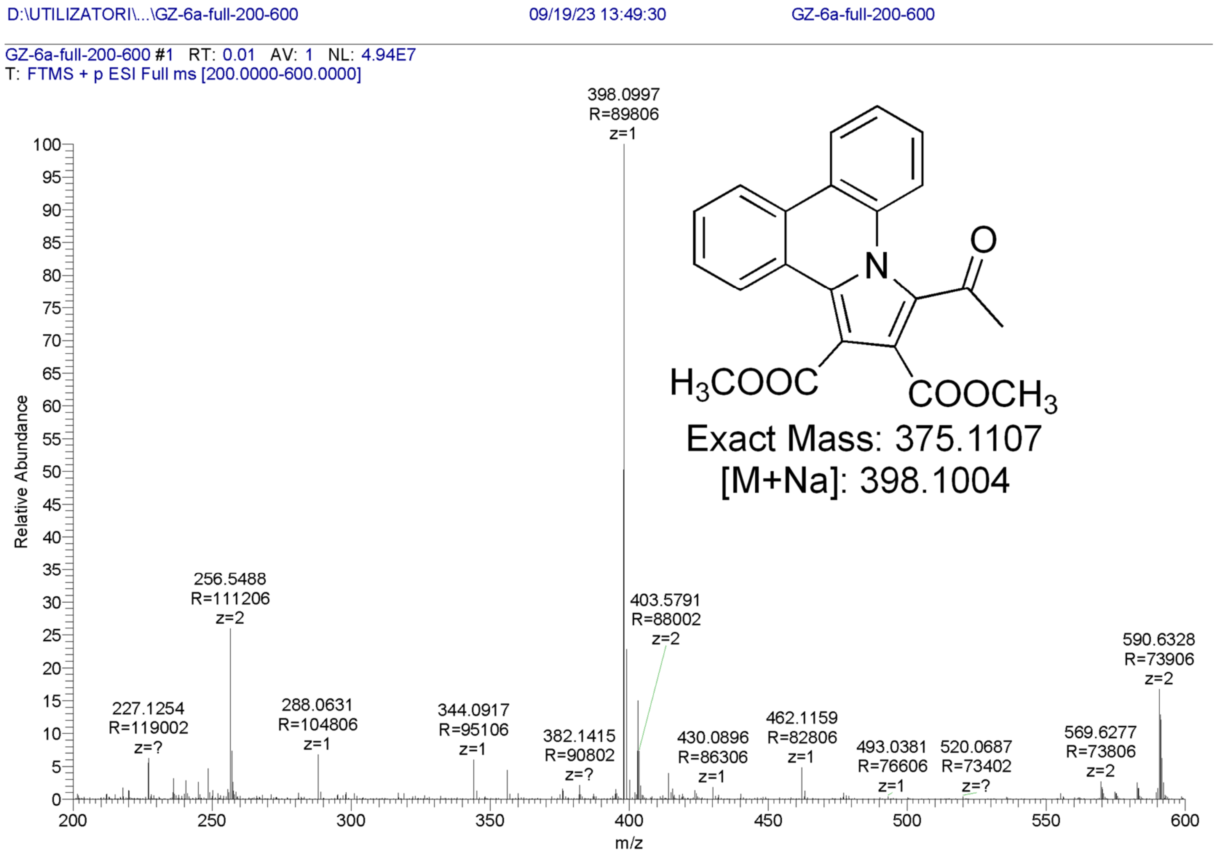

S25 Fig. HR-MS spectra of the compound **6a** in positive mode.

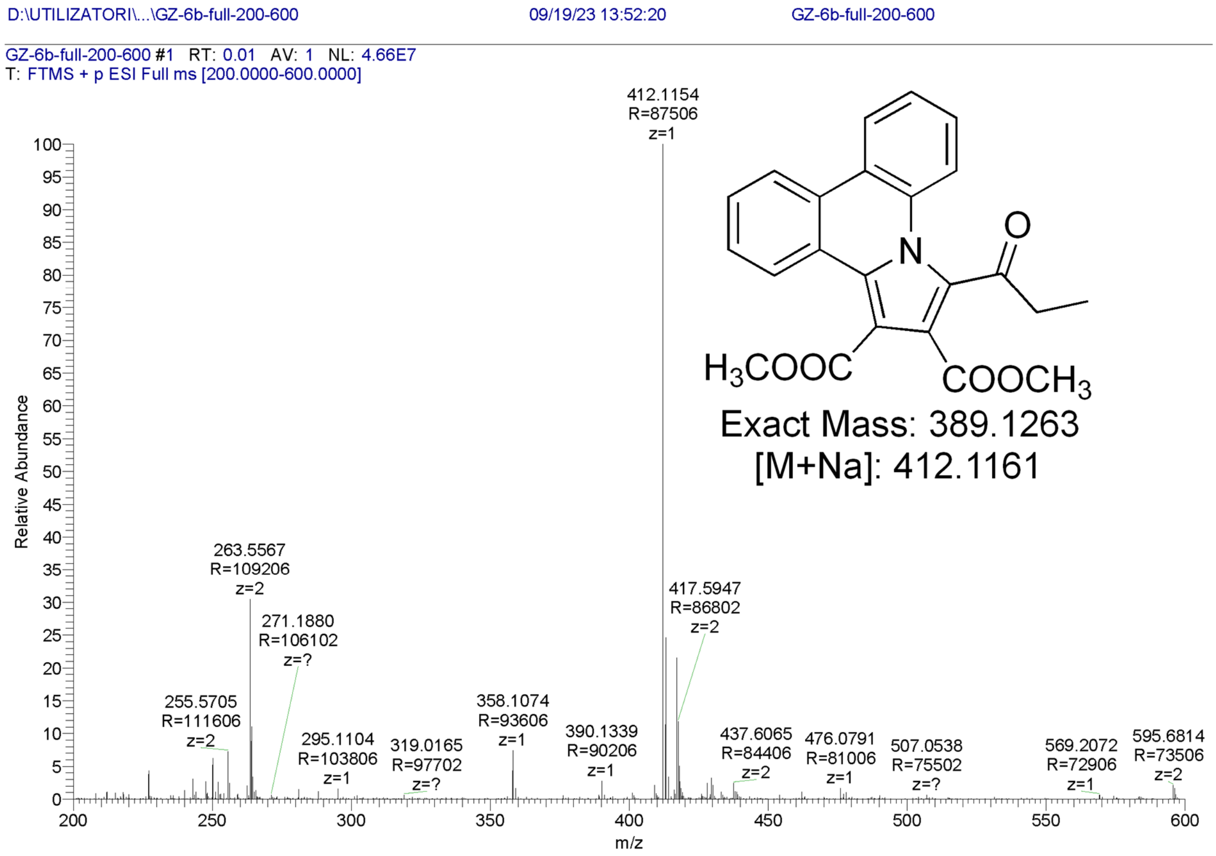

S26 Fig. HR-MS spectra of the compound **6b** in positive mode.

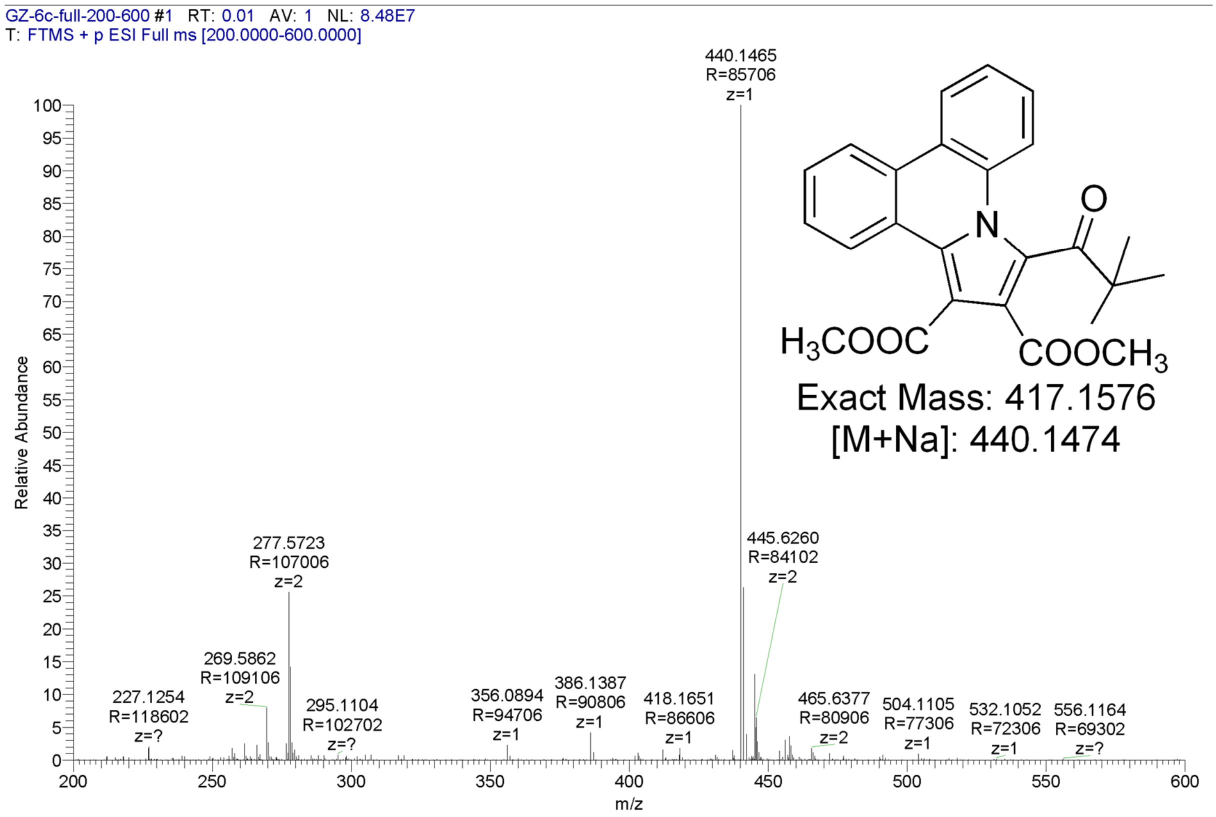

S27 Fig. HR-MS spectra of the compound **6c** in positive mode.

5. Graphical Representation for Anticancer Activity of the Obtained Compounds against Different Types of Cancer Cell Line

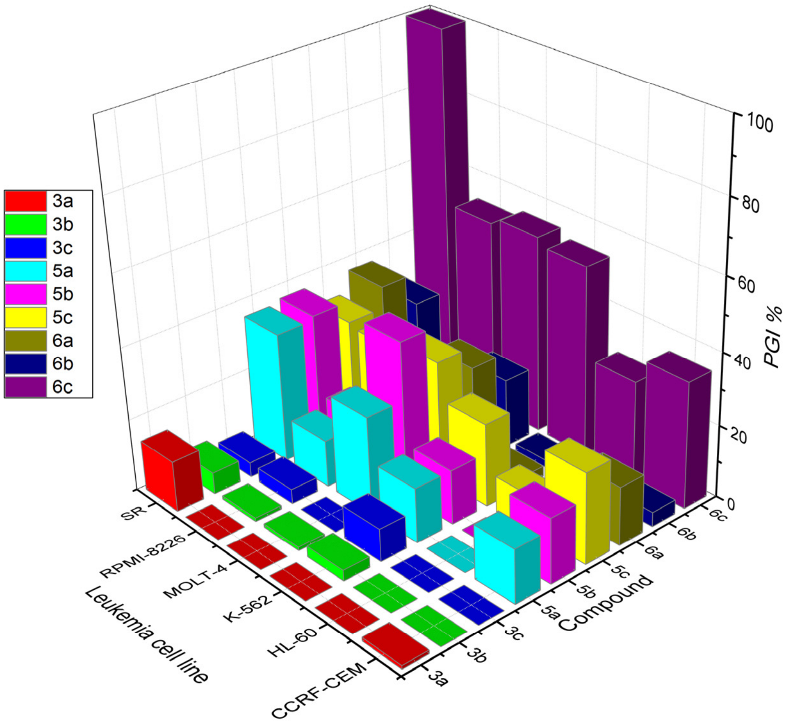

S28 Fig. Anticancer activity of the benzo[*c*]quinolinium salts **3a-c** and pyrrolobenzo[c]quinoline cycloadducts **5a-c** and **6a-c** against 6 NCI human *Leukemia* cell lines, expressed as the percentage growth inhibition.

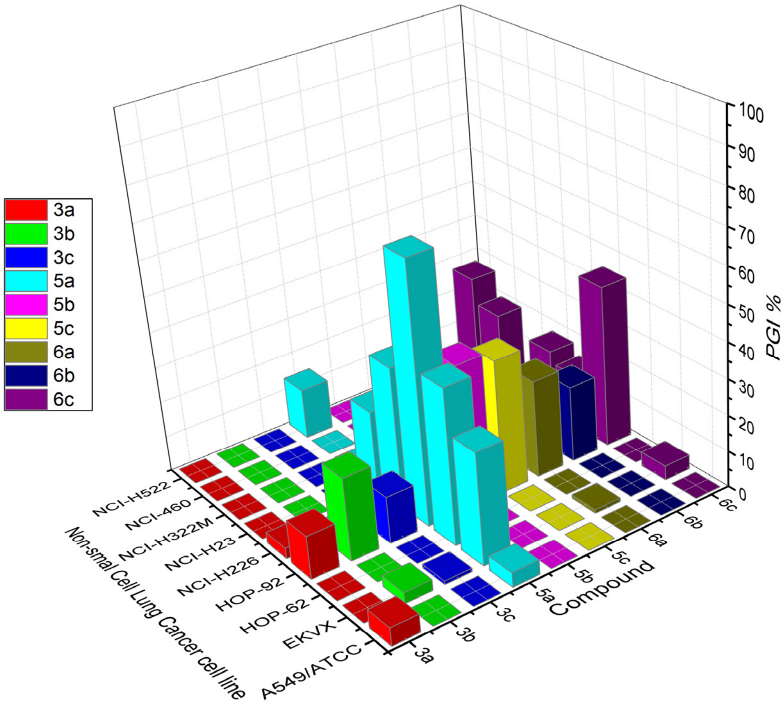

S29 Fig. Anticancer activity of the benzo[*c*]quinolinium salts **3a-c** and pyrrolobenzo[c]quinoline cycloadducts **5a-c** and **6a-c** against 9 NCI human *Non-small Cell Lung Cancer* cell lines, expressed as the percentage growth inhibition.

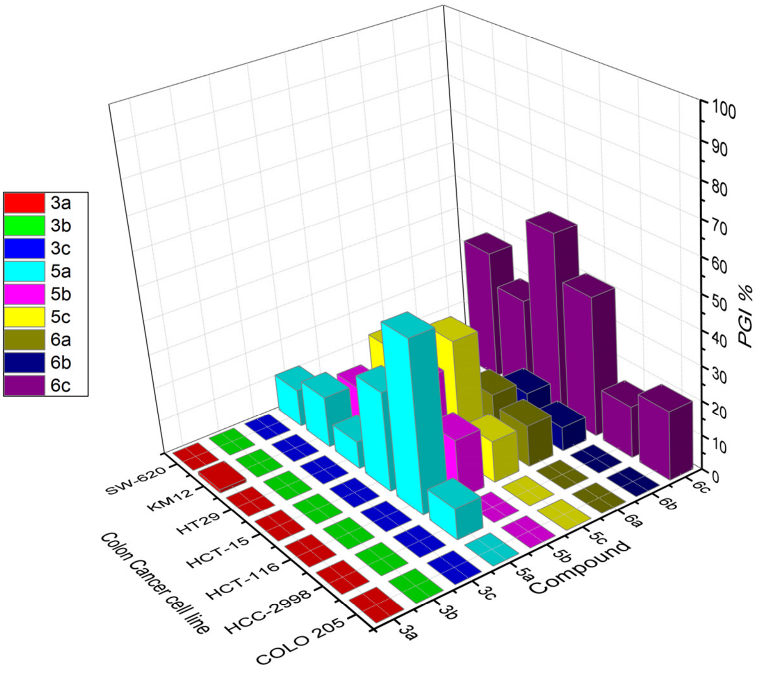

S30 Fig. Anticancer activity of the benzo[*c*]quinolinium salts **3a-c** and pyrrolobenzo[c]quinoline cycloadducts **5a-c** and **6a-c** against 7 NCI human *Colon Cancer* cell lines, expressed as the percentage growth inhibition.

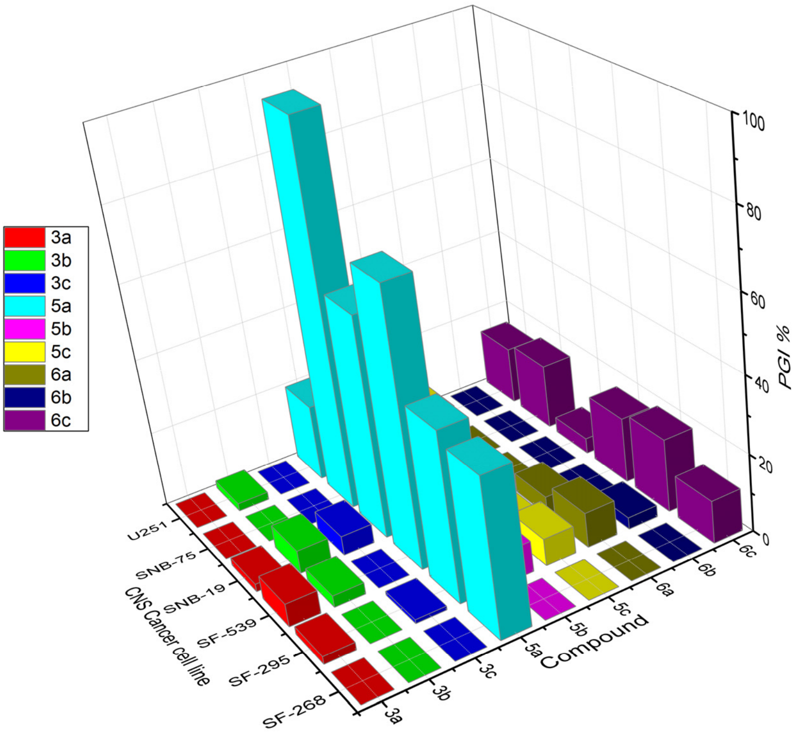

S31 Fig. Anticancer activity of the benzo[*c*]quinolinium salts **3a-c** and pyrrolobenzo[c]quinoline cycloadducts **5a-c** and **6a-c** against 6 NCI human *CNS Cancer* cell lines, expressed as the percentage growth inhibition.

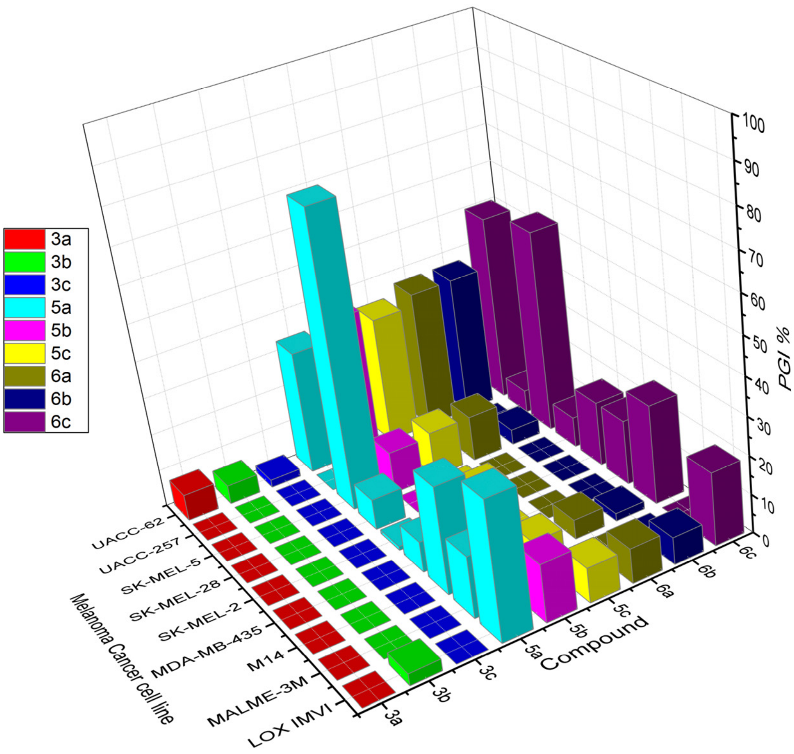

S32 Fig. Anticancer activity of the benzo[*c*]quinolinium salts **3a-c** and pyrrolobenzo[c]quinoline cycloadducts **5a-c** and **6a-c** against 9 NCI human *Melanoma* cell lines, expressed as the percentage growth inhibition.

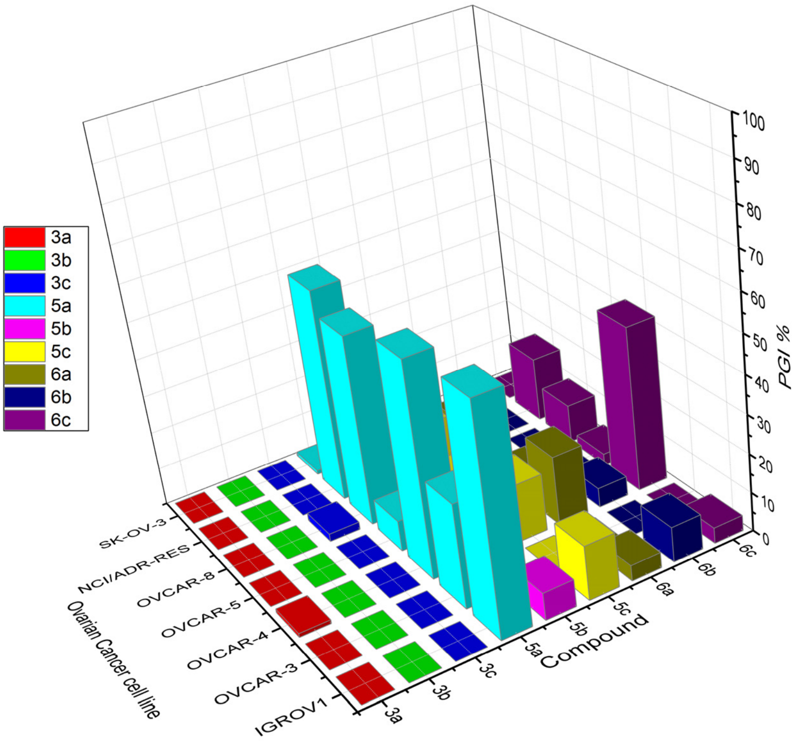

S33 Fig. Anticancer activity of the benzo[*c*]quinolinium salts **3a-c** and pyrrolobenzo[c]quinoline cycloadducts **5a-c** and **6a-c** against 7 NCI human *Ovarian Cancer* cell lines, expressed as the percentage growth inhibition.

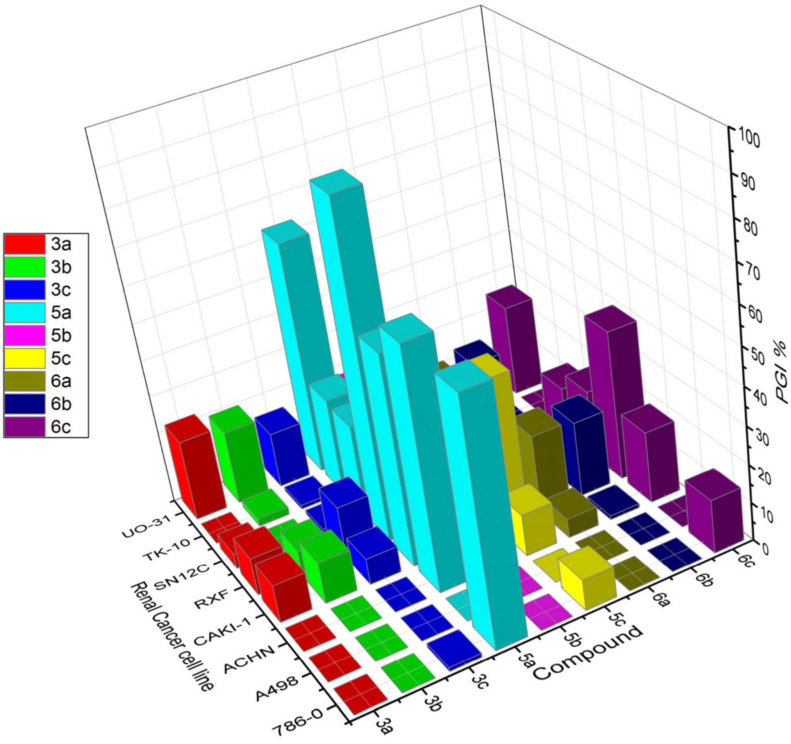

S34 Fig. Anticancer activity of the benzo[*c*]quinolinium salts **3a-c** and pyrrolobenzo[c]quinoline cycloadducts **5a-c** and **6a-c** against 8 NCI human *Renal Cancer* cell lines, expressed as the percentage growth inhibition.

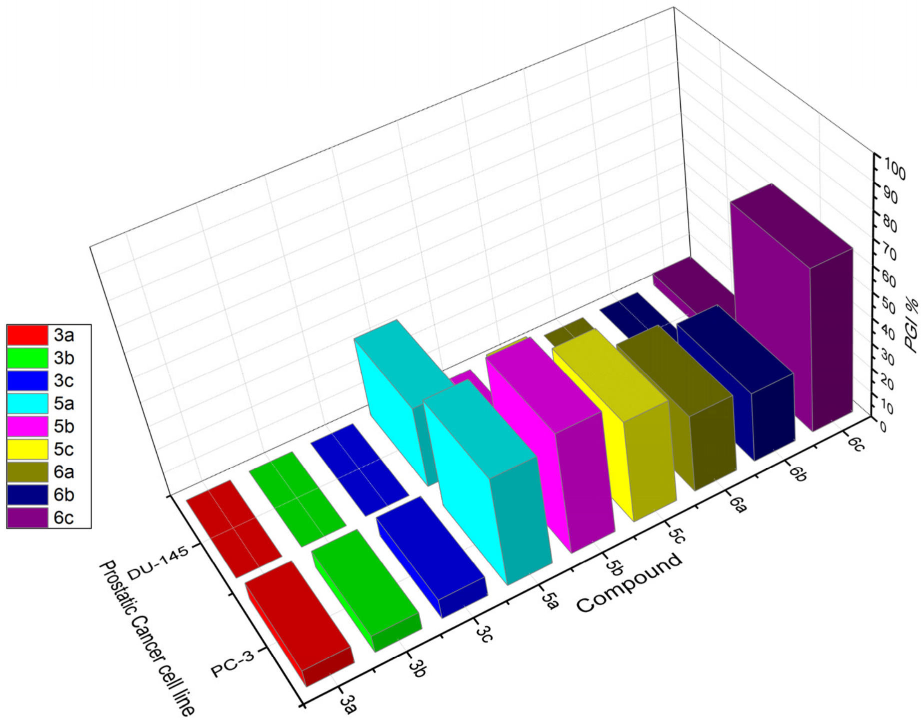

S35 Fig. Anticancer activity of the benzo[*c*]quinolinium salts **3a-c** and pyrrolobenzo[c]quinoline cycloadducts **5a-c** and **6a-c** against 2 NCI human *Prostate Cancer* cell lines, expressed as the percentage growth inhibition.

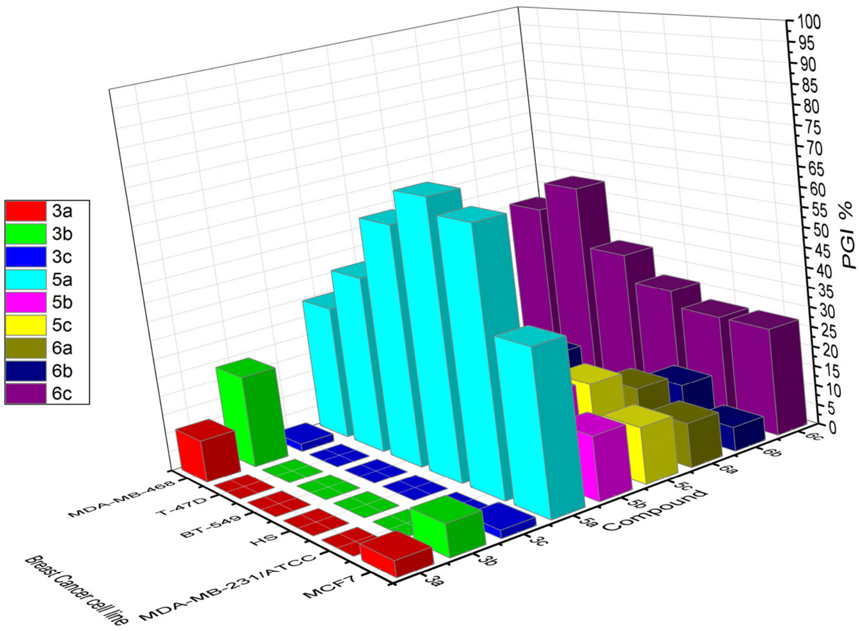

S36 Fig. Anticancer activity of the benzo[*c*]quinolinium salts **3a-c** and pyrrolobenzo[c]quinoline cycloadducts **5a-c** and **6a-c** against 6 NCI human *Breast Cancer* cell lines, expressed as the percentage growth inhibition.

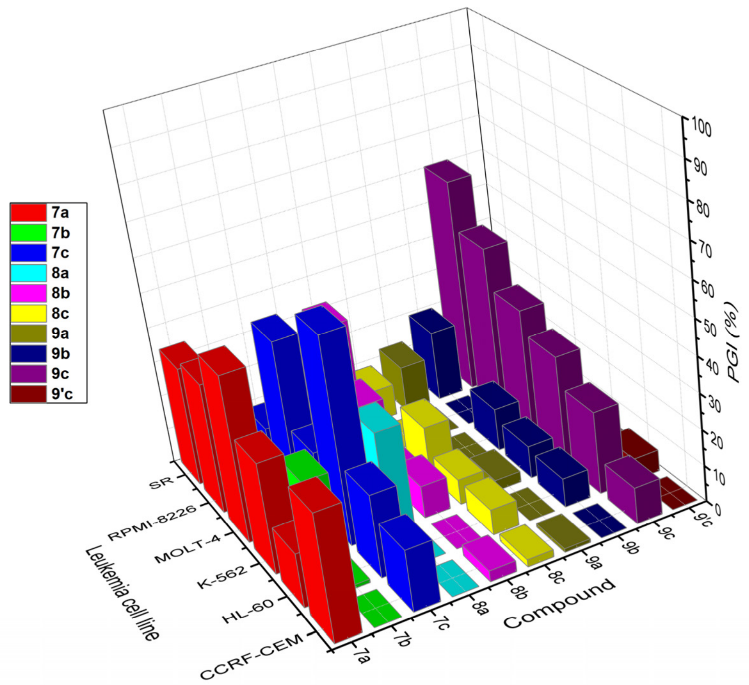

S37 Fig. Anticancer activity of the benzo[*f*]quinolinium salts **7a-c** and pyrrolobenzo[f]quinoline cycloadducts **8a-c**, **9a-c** and **9’c** against 6 NCI human *Leukemia* cell lines, expressed as the percentage growth inhibition.

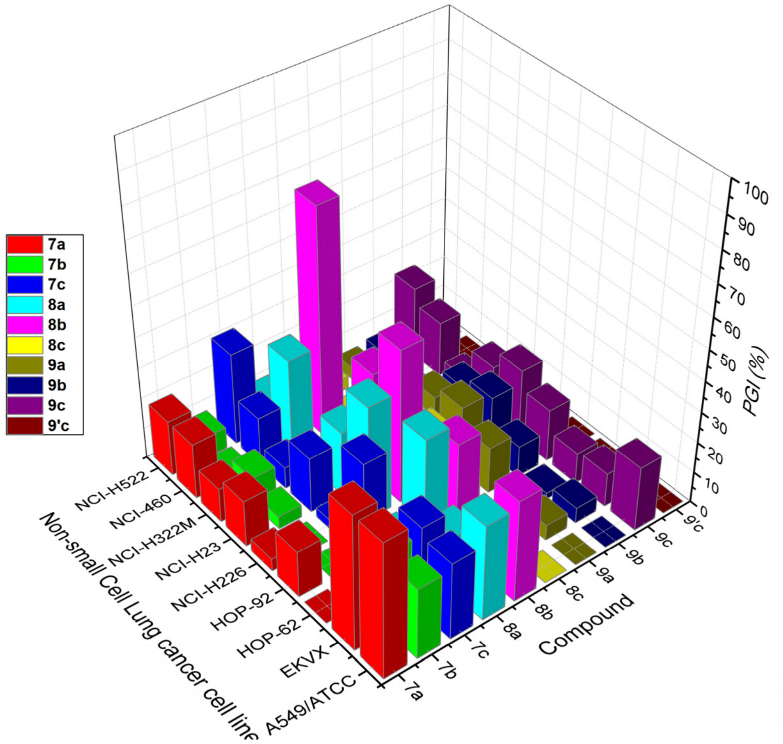

S38 Fig. Anticancer activity of the benzo[*f*]quinolinium salts **7a-c** and pyrrolobenzo[f]quinoline cycloadducts **8a-c**, **9a-c** and **9'c** against 9 NCI human *Non-small Cell Lung Cancer* cell lines, expressed as the percentage growth inhibition.

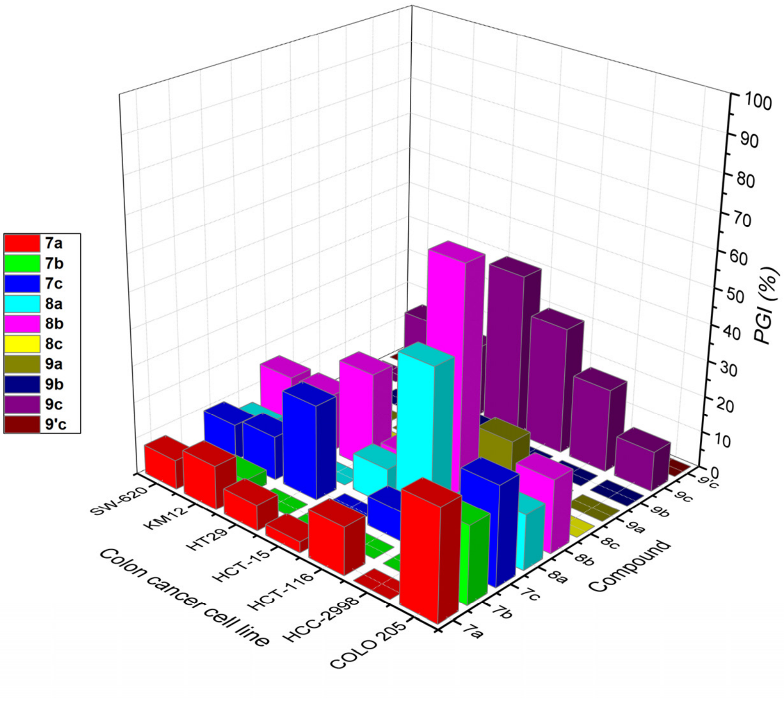

S39 Fig. Anticancer activity of the benzo[*f*]quinolinium salts **7a-c** and pyrrolobenzo[f]quinoline cycloadducts **8a-c**, **9a-c** and **9'c** against 7 NCI human *Colon Cancer* cell lines, expressed as the percentage growth inhibition.

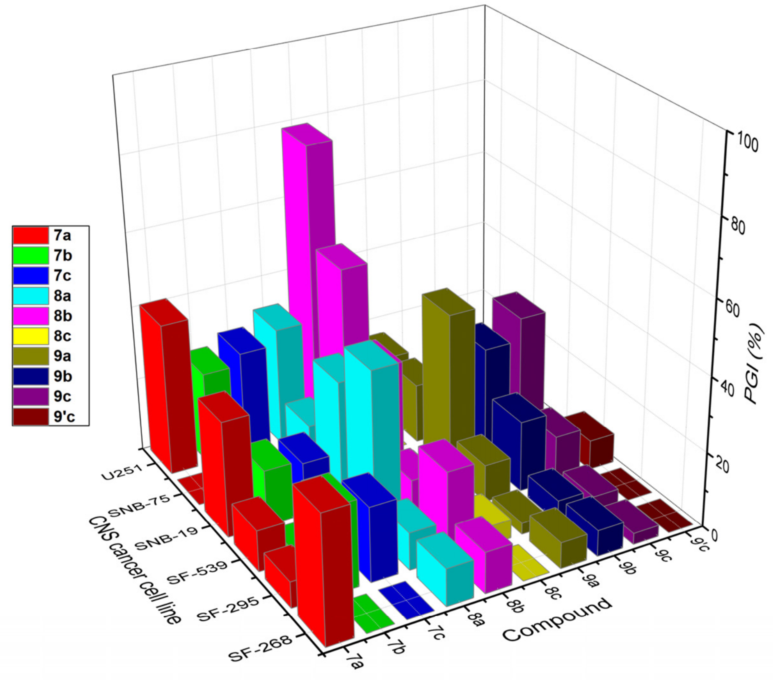

S40 Fig. Anticancer activity of the benzo[*f*]quinolinium salts **7a-c** and pyrrolobenzo[f]quinoline cycloadducts **8a-c**, **9a-c** and **9’c** against 6 NCI human *CNS Cancer* cell lines, expressed as the percentage growth inhibition.

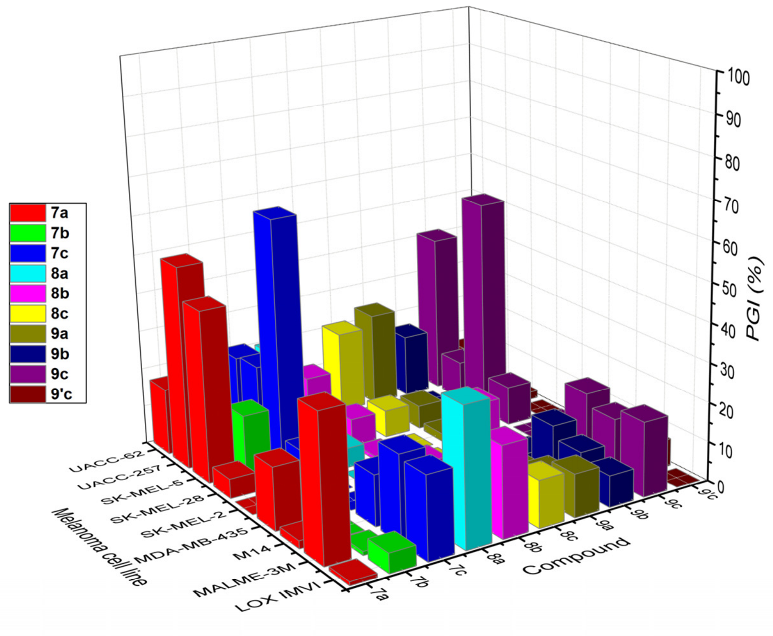

S41 Fig. Anticancer activity of the benzo[*f*]quinolinium salts **7a-c** and pyrrolobenzo[f]quinoline cycloadducts **8a-c**, **9a-c** and **9’c** against 9 NCI human *Melanoma* cell lines, expressed as the percentage growth inhibition.

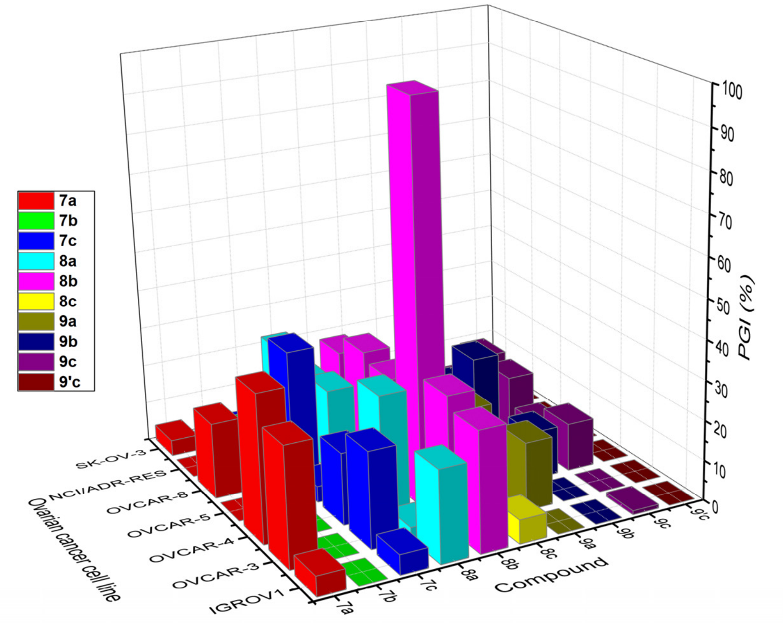

S42 Fig. Anticancer activity of the benzo[*f*]quinolinium salts **7a-c** and pyrrolobenzo[f]quinoline cycloadducts **8a-c**, **9a-c** and **9'c** against 7 NCI human *Ovarian Cancer* cell lines, expressed as the percentage growth inhibition.

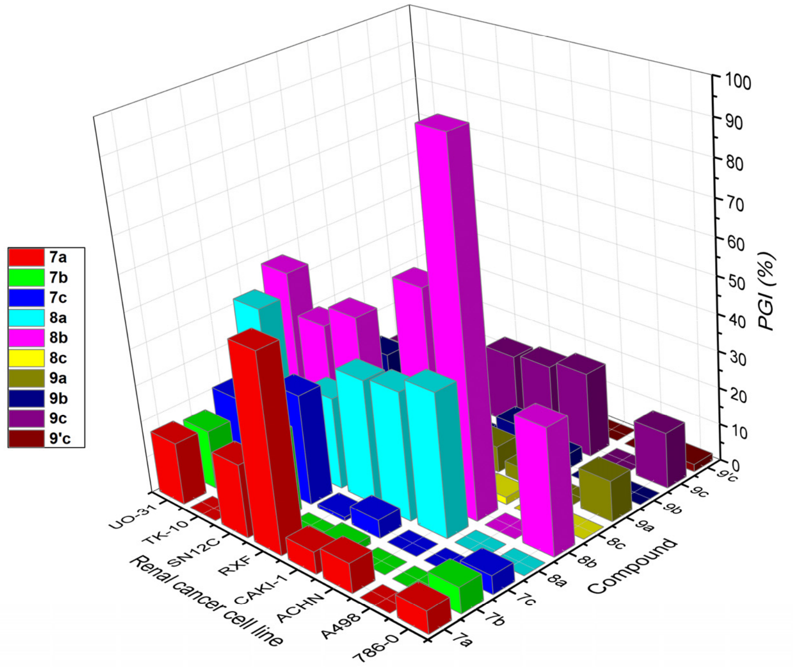

S43 Fig. Anticancer activity of the benzo[*f*]quinolinium salts **7a-c** and pyrrolobenzo[f]quinoline cycloadducts **8a-c**, **9a-c** and **9'c** against 8 NCI human *Renal Cancer* cell lines, expressed as the percentage growth inhibition.

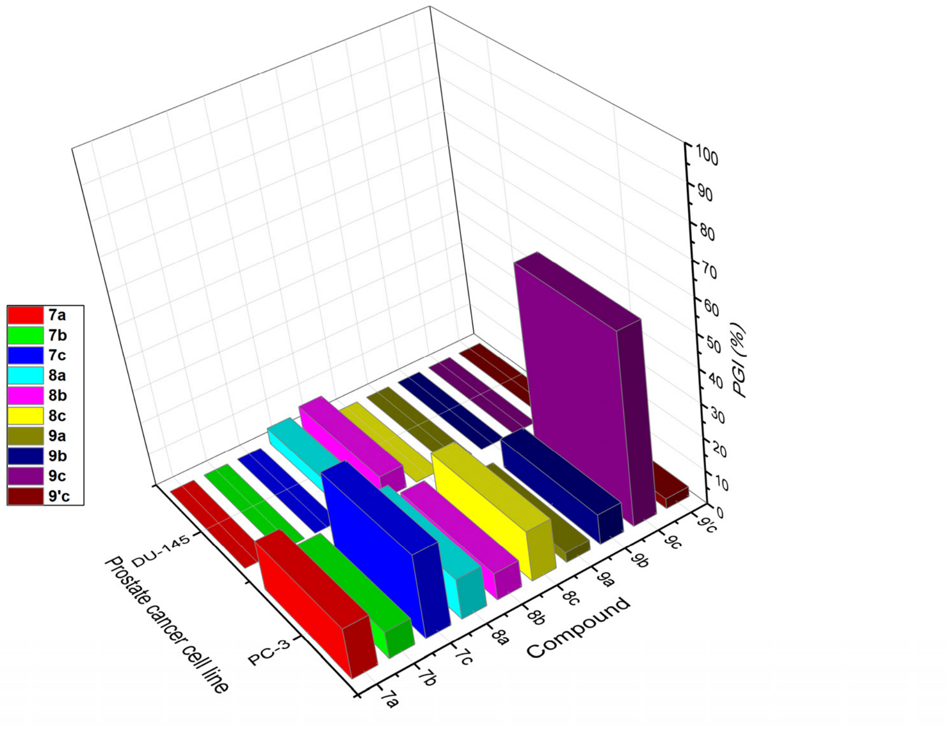

S44 Fig. Anticancer activity of the benzo[*f*]quinolinium salts **7a-c** and pyrrolobenzo[f]quinoline cycloadducts **8a-c**, **9a-c** and **9'c** against 2 NCI human *Prostate Cancer* cell lines, expressed as the percentage growth inhibition.

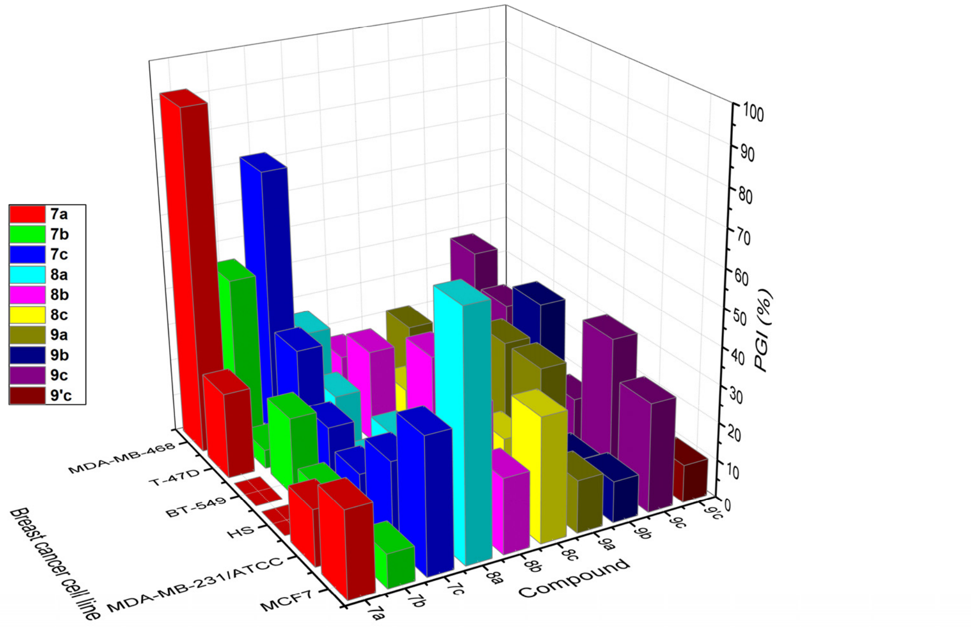

S45 Fig. Anticancer activity of the benzo[*f*]quinolinium salts **7a-c** and pyrrolobenzo[f]quinoline cycloadducts **8a-c**, **9a-c** and **9'c** against 6 NCI human *Breast Cancer* cell lines, expressed as the percentage growth inhibition.
